# Supplementary material for: Earnings among people with multiple sclerosis compared to references, in total and by educational level and type of occupation: a population-based cohort study at different points in time
Source: BMJ Open. 2019 Jul 11;9(7):e024836. doi: 10.1136/bmjopen-2018-024836 (PMC6629418; doi:10.1136/bmjopen-2018-024836)
Supplement: Supplementary data [file bmjopen-2018-024836supp001.pdf]

Supplementary Table 1: Cross-sectional Tobit regressions for earnings [a] for the year of inclusion [b] and five years later including interactions between MS and education or occupation, respectively.

	**Education** **Model 1b**		**Occupation** **Model 2b**	
	Year of inclusion (**T$_0$**) Estimated difference [c] (CI 95%)	Five years later (**T$_{+5}$**) Estimated difference [c] (CI 95%)	Year of inclusion (**T$_0$**) Estimated difference [c] (CI 95%)	Five years later (**T$_{+5}$**) Estimated difference [c] (CI 95%)
Intercept	214.7 (205.5, 223.9)*	282.8 (271.8, 293.8)*	241.8 (234.5, 249.1)*	278.2 (270.0, 286.4)*
MS (ref: No MS)[d]	-26.9 (-36.7, -17.1)*	-72.3 (-83.9, -60.7)*	-33.3 (-40.4, -26.2)*	-45.5 (-53.5, -37.5)*
Male (ref: Female)[d]	68.2 (62.5, 73.9)*	67.9 (61.2, 74.6)*	63.4 (58.9, 67.9)*	64.4 (59.1, 69.7)*
Age (ref: 30-34)[d]				
- 35-39	17.5 (9.3, 25.7)*	20.6 (11.0, 30.2)*	9.4 (2.9, 15.9)*	18.4 (11.0, 25.8)*
- 40-44	35.5 (27.5, 43.5)*	25.1 (15.7, 34.5)*	25.6 (19.3, 31.9)*	27.4 (20.0, 34.8)*
- 45-49	33.5 (25.1, 41.9)*	6.2 (-3.8, 16.2)	24.2 (17.5, 30.9)*	16.6 (8.8, 24.4)*
- 50-54	30.0 (21.6, 38.4)*	-9.5 (-19.5, 0.5)	22.2 (15.5, 28.9)*	9.7 (1.7, 17.7)*
Country of birth (ref: Sweden)[d]	-78.5 (-86.1, -70.9)*	-72.4 (-81.4, -63.4)*	-20.1 (-26.4, -13.8)*	-19.9 (-27.3, -12.5)*
Panel (ref: 2006)[d]				
-2003	-5.3 (-12.7, 2.1)	-9.5 (-18.3, -0.7)*	-6.9 (-12.8, -1.0)*	-9.4 (-16.3, -2.5)*
-2004	-5.9 (-13.5, 1.7)	-2.2 (-11.2, 6.8)	-7.3 (-13.4, -1.2)*	-2.5 (-9.6, 4.6)
-2005	-7.9 (-15.5, -0.3)*	-6.8 (-15.8, 2.2)	-6.0 (-12.1, 0.1)	-3.3 (-10.4, 3.8)
Size of living region (ref: Larger cities)[e]				
- Medium-sized municipalities	-19.7 (-25.8, -13.6)*	-23.1 (-30.4, -15.8)*	-20.8 (-25.7, -15.9)*	-21.7 (-27.4, -16.0)*
- Smaller municipalities	-26.0 (-32.7, -19.3)*	-32.7 (-40.5, -24.9)*	-29.5 (-34.8, -24.2)*	-31.5 (-37.8, -25.2)*
Education (ref: University/college)[d]				
- High school	-53.7 (-60.4, -47.0)*	-75.9 (-83.5, -68.3)*	-	-
- Elementary school	-110.3 (-119.9, -100.7)*	-139.9 (-151.3, -128.5)*	-	-
- MS* High school	**-16.9 (-29.6, -4.2)***	**-14.9 (-30.0, 0.2)**	-	-
- MS* Elementary school	**-11.6 (-32.4, 9.2)**	**-42.7 (-68.0, -17.4)***	-	-
Type of Occupation (ref: Office work)[e]				
- Managers	-	-	61.2 (48.3, 74.1)*	95.7 (83.5, 107.9)*
- Manual labour	-	-	-64.5 (-69.6, -59.4)*	-71.2 (-77.1, -65.3)*
- Unknown	-	-	-138.4 (-149.6, -127.2)*	-165.2 (-183.4, -147.0)*
- No Work	-	-	-390.5 (-402.5, -378.5)*	-523.7 (-546.4, -501.0)*
- MS*Managers	-	-	**8.9 (-16.6, 34.4)**	**-40.7 (-68.5, -12.9)***
- MS* Manual labour	-	-	**-5.0 (-15.2, 5.2)**	**-5.6 (-17.8, 6.6)**
- MS* Unknown	-	-	**8.9 (-12.7, 30.5)**	**-24.7 (-60.2, 10.8)**
- MS* No Work	-	-	**27.0 (3.7, 50.3)***	**14.8 (-26.4, 56.0)**
Sigma	131.1 (129.1, 133.1)*	152.3 (149.9, 154.7)*	100.6 (99.0, 102.2)*	113.7 (111.9, 115.5)*
N	10,137	9,928	10,138	9,928
N censored at zero	1,260	1,505	1,260	1,505
Bayesian information criterion (BIC)	114,739	111,979	107,898	104,333

[a] Earnings: pre-tax earnings including student allowances. Presented in 2005 values, in SEK 1000.
[b] Year of inclusion (T$_0$) for people with multiple sclerosis (PwMS): year of first MS diagnosis. Year of inclusion (T$_0$) for references: year of inclusion to one of four panels. Estimates from Tobit models with lower bound set at zero (i.e., no earnings).
[c] Beta estimate
[d] Time-invariant variables: measured at T$_0$.
[e] Time-variant variables: measured at T$_0$ or T$_{+5}$.
* Differences significantly different from zero ($p<0.05$).
