# Supplementary material for: Earnings among people with multiple sclerosis compared to references, in total and by educational level and type of occupation: a population-based cohort study at different points in time
Source: BMJ Open. 2019 Jul 11;9(7):e024836. doi: 10.1136/bmjopen-2018-024836 (PMC6629418; doi:10.1136/bmjopen-2018-024836)
Supplement: Author's manuscript [file bmjopen-2018-024836.draft_revisions.pdf]

BMJ Open

BMJ Open is committed to open peer review. As part of this commitment we make the peer review history of every article we publish publicly available.

When an article is published we post the peer reviewers’ comments and the authors’ responses online. We also post the versions of the paper that were used during peer review. These are the versions that the peer review comments apply to.

The versions of the paper that follow are the versions that were submitted during the peer review process. They are not the versions of record or the final published versions. They should not be cited or distributed as the published version of this manuscript.

BMJ Open is an open access journal and the full, final, typeset and author-corrected version of record of the manuscript is available on our site with no access controls, subscription charges or pay-per-view fees (http://bmjopen.bmj.com).

If you have any questions on BMJ Open’s open peer review process please email info.bmjopen@bmj.com

BMJ Open

Earnings among people with multiple sclerosis compared to references, in total and by educational level and type of occupation: a population-based cohort study at different points in time

Journal:	*BMJ Open*
Manuscript ID	bmjopen-2018-024836
Article Type:	Research
Date Submitted by the Author:	15-Jun-2018

Complete List of Authors:	Wiberg, Michael; Karolinska Institutet Department of Clinical Neuroscience, Division of Insurance Medicine; Swedish Social Insurance Agency, Department of Analysis and Forecast<br>Murley, Chantelle; Karolinska Institutet Department of Clinical Neuroscience, Division of Insurance Medicine<br>Tinghög, Petter; Karolinska Institutet Department of Clinical Neuroscience, Division of Insurance Medicine; Red Cross University College<br>Alexanderson, Kristina; Karolinska Institutet Department of Clinical Neuroscience, Division of Insurance Medicine<br>Palmer, Edward; Uppsala University , Department of Economics, Uppsala Center for Labor Studies<br>Hillert, Jan; Karolinska Institutet Department of Clinical Neuroscience, Division of Neurology<br>Stenbeck, Magnus; Karolinska Institutet Department of Clinical Neuroscience, Division of Insurance Medicine<br>Friberg, Emilie; Karolinska Institutet Department of Clinical Neuroscience, Division of Insurance Medicine

Keywords:	Multiple sclerosis < NEUROLOGY, Earnings, Sick leave, Disability pension, Work, Social Insurance

SCHOLARONE™
Manuscripts

Earnings among people with multiple sclerosis compared to references, in total and by educational level and type of occupation: a population-based cohort study at different points in time.

Michael Wiberg[1,2], Chantelle Murley[1], Petter Tinghög[1,3], Kristina Alexanderson[1], Edward Palmer[4], Jan Hillert[5], Magnus Stenbeck[1] and Emilie Friberg[1].

[1] Department of Clinical Neuroscience, Division of Insurance Medicine, Karolinska Institutet, SE 171 77 Stockholm, Sweden.

[2] Department of Analysis and Forecast, Swedish Social Insurance Agency, 103 51 Stockholm, Sweden.

[3] Department of Health Sciences, Swedish Red Cross University College, 141 21 Huddinge, Sweden.

[4] Uppsala Department of Economics and Center for Labor Studies, 753 13 Uppsala, Sweden.

[5] Department of Clinical Neuroscience, Division of Neurology, Karolinska Institutet, SE 171 77 Stockholm, Sweden.

Corresponding author: Chantelle Murley

Phone +46(0)8524 832 24

Fax +46(0)8524 832 05

wiberg.michael@gmail.com, chantelle.murley@ki.se, Petter.Tinghog@rkh.se, Kristina.Alexanderson@ki.se, Edward.E.Palmer@gmail.com, Jan.Hillert@ki.se, Magnus.Stenbeck@ki.se, Emilie.Friberg@ki.se.

Keywords (6): MS, Earnings, Sick leave, Disability pension, Work, Social insurance.

Word counts:

Abstract: 298 (max 300)

Main text excluding title page, abstract, references, figures and tables: 4554

Number of references: 62.

Number of tables: 4.

Number of figures: 2.

For peer review only

For peer review only - http://bmjopen.bmj.com/site/about/guidelines.xhtml

ABSTRACT

Objectives

To investigate the mean levels and distributions of earnings among people with multiple sclerosis (PwMS) before and after MS diagnosis compared to people without MS, and if they were associated with different educational levels and types of occupations. Furthermore, to assess the prevalence of sickness absence (SA) and disability pension (DP) in the two groups.

Design

Population-based longitudinal cohort study (10 before until 5 years after MS diagnosis).

Setting

Working-age population using microdata linked from nationwide Swedish registers.

Participants

Residents in Sweden in 2004 aged 30-54 with MS diagnosed in 2003-2006 (n=2553), and references without MS (n=7584) randomly selected by stratified matching (sex and age).

Outcome measures

Quartiles of earnings were calculated for each year prior to and following the MS diagnosis. Mean earnings, by educational level and type of occupation, before and after diagnosis were compared using t-tests. Tobit regressions investigated the associations of earnings with individual characteristics.

Results

Differences in earnings between PwMS and the references were observed beginning one year prior to diagnosis, with differences increasing with time. PwMS had lower mean earnings for

For peer review only

For peer review only - http://bmjopen.bmj.com/site/about/guidelines.xhtml

the diagnosis year (difference= SEK 28,000, 95% CI: -33,720, -11,280), and five years after diagnosis the difference had more than doubled (95% CI: -69,680, -56,320). These differences remained after including educational level and type of occupation. Overall, the earnings of PwMS with university education and/or more qualified occupations were most alike their respective reference peers. The prevalence of SA and DP were higher for PwMS than for the references.

Conclusions

The results suggest that the earnings among working-aged PwMS are lower than the references' from shortly before diagnosis, with this gap increasing thereafter. Besides full- and part-time SA and DP, the results indicate that educational level and type of occupation are influential factors behind the large heterogeneity in PwMS' earnings.

For peer review only

ARTICLE SUMMARY

Strengths and limitations:

- The main strengths of this study include the population-based longitudinal cohort design, use of high-quality nationwide registers and large cohort of people with newly registered MS diagnoses which enabled analyses of earnings on a nationwide scale covering 16 years per individual and comparisons with a matched reference group.
- That information from all resident in Sweden with both the PwMS and reference group limited potential selection bias, a feature not often the case for these types of studies.
- Extensive annual data on socio-demographics, occupation, and sickness absence and disability pension could be included for ten consecutive years before the year of MS diagnosis and five years after, to further understand work attachment of PwMS.
- A weakness was that earnings included the sick pay for employees with short-term sickness absence spells (≤14 days) which might underestimate the effect of MS on earnings if PwMS have a higher frequency of such spells than the reference group, as lost earnings due to sickness absence are not fully compensated.
- Another weakness was that we only had information on the diagnosis date rather than on the onset date, however, it is unknown whether there are systematic differences within the MS cohort regarding the time between onset and diagnosis.

For peer review only - http://bmjopen.bmj.com/site/about/guidelines.xhtml

For peer review only

BACKGROUND

Multiple sclerosis (MS) is a chronic, often progressive, disease that usually onsets in working ages.[1] The combination of the relatively early onset age and chronic nature may affect the individual's economic welfare for a substantial part of their working life, through reduced productivity (and wage) per unit of time worked and/or the number of hours worked. Furthermore, MS poses a high cost to society in terms of both direct and indirect costs (from the productivity losses).[2-8] Previous studies have predominantly focused on societal cost burdens, whereas the effects of MS on the development of individuals' earnings, including possible differences by educational level and type of occupation have not been extensively researched.

Several studies have found that people with MS (hereafter "PwMS") to a greater extent are not in paid work compared to individuals without MS.[9-14] However, sorting individuals into conventional labour market categories (e.g., employed/unemployed) obscures more nuanced information about the gradual effect of the disease on the individuals' labour market attachment.

An alternative measure for labour market attachment is the development of individuals' earnings. There are a growing number of studies (including MS-specific[11 13-18]) showing negative associations between chronic diseases and individuals' earnings.[10 11 13-25]

The progression of MS is heterogeneous.[26 27] Accordingly, it is difficult to predict disease progression at the individual level.[28] Age at onset of symptoms is one of the few socio-demographic factors that have been shown to be predictive of disease course.[29 30] Further, younger age at onset has also been associated with current employment.[12 31-34] In addition, educational level has been shown to influence labour market participation among PwMS.[34-38]

For peer review only - http://bmjopen.bmj.com/site/about/guidelines.xhtml

Therefore, educational level may also suggest the degree of physical labour and/or flexibility at the workplace.[34 36-38]

In working-aged individuals, educational level and type of occupation are highly correlated (e.g., university education increases possibilities for more qualified occupations). However, this does not necessarily hold for individuals with chronic diseases, as the disease might hinder them from performing the work corresponding to their educational level. Furthermore, one's educational level may remain unchanged above a certain age whereas an individual's occupation can vary over time, both due to individual and macro-economic factors. For this reason, in order to analyse the effects of a progressively disabling disease on work, the actual work performed (i.e., earnings), may be more informative than the individual's educational level.

Furthermore, PwMS to a high extent work part-time.[8 37 39 40] This is especially relevant for Sweden, where compensation for sickness absence (SA) and disability pension (DP) can be for either part- or full-time absences from ordinary working hours. Increasing utilisation of these compensation systems, which do not fully compensate the lost earnings, would over time result in increasing differences in annual earnings between PwMS and other wage earners, with all other factors equal.[15] Studies of such associations comparing different time points are still very few and the possible differences by the individuals' educational level and the type of work remain largely unexplored. The present study aims at improving our knowledge in this area.

The aims of this study were three-fold:

1. To examine levels and distributions of earnings of PwMS compared to matched references from the general population, during the years prior to and after the year of diagnosis with MS;

For peer review only - http://bmjopen.bmj.com/site/about/guidelines.xhtml

2. To explore if the level of education and the type of occupation correlate with the within group differences in earnings among PwMS; and
3. To compare the rates of part- or full-time SA and DP benefits between PwMS and the matched references.

METHODS

A population-based longitudinal cohort study was conducted using information from Statistics Sweden on all individuals living in Sweden on 31 December 2004. The study population was attained in two steps.

Firstly, we identified all individuals aged 30-54 years, who in 2003-2006 had their first registered MS diagnosis (ICD-9: 340, ICD-10: G35). Information on MS diagnoses was obtained from the following nationwide registers: inpatient hospital care (data from 1987-2006), specialised outpatient care (2001-2006), and outpatient surgery (1997-2000) from the National Patient Register maintained by the National Board of Health and Welfare, and days compensated by the Social Insurance Agency of SA (2005-2006), and DP (1994-2006). Based on the year of their first registered MS code, the PwMS were categorised into four panels: 2003, 2004, 2005, and 2006. Linkage across registers was conducted by using the unique identity number assigned to all residents in Sweden

Secondly, a reference group was obtained from the same source population as the PwMS. Group-based stratified matching on the joint distribution of sex and age categories was used to form a comparable reference group for each panel in light of the observed differences in the age and sex distribution of the PwMS. Three references were obtained for each MS patient, and drawn without replacement. The reference individuals had no registered MS diagnosis in 1987-2006.

For peer review only - http://bmjopen.bmj.com/site/about/guidelines.xhtml

In total, 2656 PwMS and 7950 reference individuals were included. The year a panel was constituted with newly diagnosed PwMS and reference individuals is, hereafter, referred to as "the year of inclusion" (T_0). The years the four panels were constituted were 2003, 2004, 2005, and 2006, respectively. Observation spanned from ten years prior (T_{-10}) to the year of inclusion (T_0) until five years after inclusion (T_{+5}).

Socio-demographic and income variables were obtained from the Longitudinal Integration Database for Health Insurance and Labour Market Studies (LISA), maintained by Statistics Sweden, for each of the 16 years studied (T_{-10} until T_{+5}) given residence in Sweden at the end of the respective year.

Outcome variable

Levels of earnings before tax were measured over 16 years (T_{-10} to T_{+5}). Student allowances were added to earnings to limit the risk of underestimating younger individuals' income-generating ability in earlier years. In the Swedish sickness absence insurance system, the employer usually pays for day 2-14 of a SA spell.[41] This amount could not be disentangled, and was thus included in our definition of earnings.[42]

All analyses were performed using the annual earnings in Swedish Krona (SEK), inflated to 2005 monetary values by Statistics Sweden's Consumer Price Index.

Explanatory variables

The explanatory variables were: sex (*female, male*), age (categorised in *five-year intervals*), country of birth (*Sweden, outside of Sweden*), educational level (*elementary school*, *high school, university/college*), and type of living area (based on population density: s*maller municipalities, medium-sized municipalities, larger cities*). Further, using a definition set by Statistics Sweden (based on annual income and verifications from workplace), individuals were categorised as being in work during the year in question, work status (*yes*, *no*).[42 43]

For peer review only - http://bmjopen.bmj.com/site/about/guidelines.xhtml

Values for variables pertained to the 31 December of the year of inclusion, except for the type of living area and work status which were obtained annually.

Annual information on type of occupation was also included. Usually, measures of occupation categorise occupations into a hierarchical order, based on the level of specialised skill.[44] Such categorisation is used by Statistics Sweden with ten mutually exclusive groups.[42] However, we also aimed to differentiate between types of occupations by how physically demanding they were, and to what extent the individual was able to have control over his/her working hours (i.e., flexibility at the workplace). Therefore, we collapsed the ten categories into the following five:

1. **Manager**: Individuals classified as managing a group of workers. Managers performed either office work or manual labour. Therefore, this group was analysed separately.
2. **Office work**: Non-physical ("white collar") work.
3. **Manual labour**: Physically demanding ("blue collar") work.
4. **Workplace unknown**: Registered as employed but no information on workplace.
5. **No work**: Not categorised as being in work (i.e., work status: no).

Individuals identified as employed by the armed forces were excluded from the study population (n=18; <0.1%) due to being only a few observations and insufficient information to classify occupation.

Study population

Individuals with missing values for at least one of the socio-demographic variables in the year of inclusion were excluded using list-wise deletion. An exception to this was type of occupation (due to individuals without work). Individuals who had earnings higher than the 99th percentile (of this study) in at least one year were also excluded to control for extreme

For peer review only - http://bmjopen.bmj.com/site/about/guidelines.xhtml

outliers (around 64% of those excluded in this step had earnings above the 99th percentile for more than one year). In total, 112 PwMS and 430 references were excluded from the study, due to employment in armed forces, missing values, or having earnings higher than the 99th percentile in a single year. Accordingly, due to these exclusions, the total number of references does not sum to three times the number of PwMS.

The maximum number of years with information for each of the individuals in the study was 16. Due to migration before/after the year of inclusion or death, not all individuals were in the study population for the entire study period. Nonetheless, 94.4% of PwMS and 93.1% matched references were in the study population for all 16 years. Stratified analyses by country of birth showed that 97.2% of PwMS and 97.5% of the references born in Sweden were included for all 16 years. Corresponding proportions for individuals not born in Sweden were 70.7% and 70.5%, respectively. At the end of follow-up (T_{+5}), 2.0% of all PwMS and 2.1% of the references were no longer in the study population.

Patient involvement and ethical approval

The project was approved by the Regional Ethical Review Board of Stockholm, Sweden. Due to the use of anonymous data from total population registers, informed consent was not applicable.

Insurance system

The universal Swedish sickness absence insurance system involves two main benefits: sickness absence (SA) and disability pension (DP). From the age of 16, people with income from work or unemployment benefits can be granted SA benefits if disease or injury leads to work incapacity. People aged 19-64 can be granted DP if they have long-term or permanently reduced work capacity due to disease or injury. Both benefits can be granted at four levels

For peer review only - http://bmjopen.bmj.com/site/about/guidelines.xhtml

(25, 50, 75, or 100%) of ordinary working hours, but at different replacement rates (SA: approximately 80%, DP: approximately 64% of lost income, up to a ceiling).

Regarding SA, the employer pays the first 14 days of a SA spell, with one uncompensated ("waiting") day at the onset. A physician's certificate is required by day eight. Absence from day 15 onwards is covered by the national Social Insurance Agency, and from day 2 among unemployed.[41]

Statistical methods

The levels and distributions of earnings were analysed for PwMS and references, stratified by time before and after the year of inclusion (T_0). The four panels were pooled in all analyses.

Firstly, annual mean and median earnings were used to describe the central tendencies among PwMS and references. The annual "interquartile range" (IQR); the distance between the 25th and 75th percentile was used to describe potential converging/diverging trends within, and between, the two groups.

Secondly, mean earnings before and after T_0, stratified by educational level and type of occupation at T_0 were calculated. Two-sided t-tests tested the differences in mean earnings between PwMS and the references.

Thirdly, the proportions of individuals on part- or full-time SA and DP during follow-up, stratified by educational level and type of occupation, were examined. For individuals with multiple spells of SA or DP in a single year, the spell with the highest grade (25, 50, 75, or 100%) was selected.

Finally, Tobit regression models were used to control for potential socio-demographic confounding and to estimate differences in levels of earnings between PwMS and references. Earnings are a non-negative outcome and often exhibit a skewed distribution with clustering

For peer review only - http://bmjopen.bmj.com/site/about/guidelines.xhtml

at zero (i.e., distinct differences in distributions between those in paid work compared to those not in paid work). Earnings were thus used as an observable proxy for individuals' ability to support themselves through their own work (i.e., earnings above zero). Tobit models avoid the potential bias of analysing outcomes with "semi-continuous" distributions by ordinary least squares (OLS), which may produce negative predicted values due to assumptions of unlimited linearity in the outcome variable.[45] A Tobit model jointly estimates, using a set of explanatory variables, the probability that the individuals' outcome variable is within a pre-determined bound and the linear relationship between the explanatory variables and the outcome for individuals within the bound.[46] We used the lower bound zero (i.e., no income from work), to observe the actual outcomes, rather than unknown outcomes based on set bound(s), which also means that the observed zeroes were "genuine zeroes".[47]

Separate Tobit regressions were estimated at the time points T_0 and T_{+5} for two different models. The distributions of earnings in these two time periods are shown in Figure 1. The two models used the same set of explanatory variables (MS (*yes/no*), panel membership, sex, age at inclusion, country of birth, and type of living area), with the exception of level of education and type of occupation, which due to high correlation were analysed separately. Model 1 included the level of education at year of inclusion whereas Model 2 included a time-varying variable for the type of occupation. Statistical tests of whether the estimates deviate enough from zero to reject the assumption of no association were performed at the 95% significance level ($p<0.05$) and presented as beta estimates with 95% confidence intervals (95% CI).

RESULTS

We identified 2553 PwMS (Panel: 2003: 724; 2004: 648; 2005: 636; and 2006: 545) and included 7584 matched references. The distributions of the socio-demographics for the

For peer review only - http://bmjopen.bmj.com/site/about/guidelines.xhtml

pooled panels at the year of inclusion (T_0) are presented in Table 1 The majority of the PwMS were women (70%) and aged 30-45 when diagnosed (62%). In the year of inclusion, the majority had at least some high school education, were born in Sweden (89% among the PwMS compared to 84% in the reference group), and lived in larger cities or medium-sized municipalities. Also, in both groups, most individuals had “office work” or “manual labour” as occupations.

For peer review only

For peer review only - http://bmjopen.bmj.com/site/about/guidelines.xhtml

Table 1: Descriptive statistics for the people with multiple sclerosis (PwMS) group and stratified matched references at the year of inclusion (T_0).[a]

		PwMS N=2553[c]	%	References N=7584[c]	%
**Sex** [b]					
	Female	1796	70	5380	71
	Male	757	30	2204	29
**Age** [b]					
	30-34	494	19	1461	19
	35-39	531	21	1576	21
	40-44	574	22	1701	22
	45-49	492	19	1446	19
	50-54	462	18	1400	18
**Educational level**					
	University/college	945	37	2685	35
	High school	1345	53	3801	50
	Elementary school	263	10	1098	14
**Type of occupation**					
	Managers	86	3	259	3
	Office	1082	42	3020	40
	Manual	916	36	2976	39
	Unknown	130	5	352	5
	No work	339	13	977	13
**Type of living area**					
	Larger cities	960	38	2789	37
	Medium-sized municipalities	902	35	2681	35
	Smaller municipalities	691	27	2114	28
**Country of birth**					
	Sweden	2280	89	6356	84
	Outside of Sweden	273	11	1228	16
**Year of inclusion (T_0)**					
	2003	724	28	2150	28
	2004	648	25	1927	25
	2005	636	25	1890	25
	2006	545	21	1617	21

[a] Year of inclusion (T_0) for PwMS: year of first MS diagnosis. Year of inclusion (T_0) for references: year of inclusion to one of the four panels.
[b] Variables used for stratified matching (1_{MS}->3_{REF}).

For peer review only

For peer review only - http://bmjopen.bmj.com/site/about/guidelines.xhtml

Figure 2 shows the development and intra-year distribution of earnings T_{-10} to T_{+5}, for the PwMS and the reference group. The reference group experienced a steady increase in earnings and a predominantly even IQR across the entire period. Whereas, the PwMS' trajectory can be divided into three periods: T_{-10} to T_{-2}; T_{-1} to T_{+1}; and T_{+2} to T_{+5}. The first period (T_{-10} to T_{-2}) was characterised by increasing earnings for PwMS with an even intra-year distribution (measured by IQR). In the second period (T_{-1} to T_{+1}), PwMS' earnings were decreasing and the IQR was diverging. In the third period (T_{+2} to T_{+5}), the level of earnings was stable but IQR diverging.

Table 2 describes mean levels of earnings among PwMS and references, stratified by educational level, and type of occupation at T_0. Differences between subgroups of PwMS were compared to the corresponding reference subgroup at: T_{-5}; T_{-2}; T_{-1}; T_0; and T_{+5}. In total, PwMS had lower earnings at T_{-1}, T_0 and T_{+5} compared to the references. These differences increased with time. In T_0, PwMS' mean earnings were 85% of the references' mean earnings, whereas the corresponding proportion for T_{+5} was 73%.

PwMS with at most elementary or high school education, experienced a decrease in mean earnings from T_{-1} and onwards. For PwMS with university/college education, there was a decrease in mean earnings from T_{-1} to T_0, but an increase from T_0 to T_{+5} (Table 2). The reference group had increasing trends in mean earnings for all levels of education; which resulted in increasing differences in mean earnings between PwMS and the references. At the end of follow-up (T_{+5}) the largest differences in earnings were found among those with at most elementary school education; where the PwMS' mean earnings were SEK 84,000, corresponding to 53% of the mean earnings of their peers in the reference group. Further, PwMS with high school and university/college education also had lower earnings than their peers in the reference group at T_{+5}. The corresponding differences and proportions for high

For peer review only - http://bmjopen.bmj.com/site/about/guidelines.xhtml

school and university/college education were SEK 68,000 (68%) and SEK 63,000 (78%), respectively.

In all time periods, for both PwMS and references, the mean earnings were highest among the managers (Table 2). Further, office workers had higher mean earnings compared to those with manual work. These findings reflected those which we obtained for level of education. PwMS in manual work had lower earnings compared to their reference counterparts at T_{+5} (67%). The corresponding figure for office workers was 78%. However, the largest difference in T_{+5} was observed among managers, with SEK 113,000 less among PwMS than their peers, which corresponded to 70% of the mean earnings of the reference group managers. Finally, those with no work at T_0 had much lower levels of earnings compared to those with known occupations both prior to and after this time point.

For peer review only

For peer review only - http://bmjopen.bmj.com/site/about/guidelines.xhtml

321 **Table 2**: Mean earnings[a] of people with multiple sclerosis (PwMS) and differences in mean earnings
322 between the PwMS group and reference group at different lengths in time from the year of inclusion
323 (T_0), stratified by educational level and type of occupation at T_0.

	T_{-5} PwMS[b] (diff)[c]	T_{-2} PwMS[b] (diff)[c]	T_{-1} PwMS[b] (diff)[c]	T_0 PwMS[b] (diff)[c]	T_{+5} PwMS[b] (diff)[c]
**All**	169 (4)	182 (1)	181 (-6)*	163 (-28)*	166 (-63)*
**Educational level**					
University/college	192 (5)	214 (1)	214 (-6)	203 (-24)*	218 (-63)*
High school	159 (0)	168 (-6)	166 (-12)*	146 (-37)*	145 (-68)*
Elementary school	135 (9)	142 (12)	135 (3)	111 (-25)*	84 (-74)*
**Type of occupation**					
Managers	251 (-12)	308 (-5)	308 (-24)	306 (-27)	258 (-113)*
Office	207 (2)	230 (-5)	233 (-11)*	219 (-33)*	226 (-65)*
Manual	163 (6)	179 (4)	178 (-4)	152 (-36)*	146 (-71)*
Unknown	96 (6)	90 (-5)	99 (-7)	102 (-18)	88 (-69)*
No work	65 (13)*	39 (10)*	20 (6)*	4 (0)	25 (-27)*

324 [a] Earnings: income from work and student allowances, presented in 2005 values as 1000 Swedish Krona (SEK).
325 [b] PwMS (n=2553). References (n=7584).
326 [c] Differences in mean earnings (SEK 1000) between PwMS and references in parentheses. Calculated with two-sided t-tests with unequal
327 variance and unequal numbers of observations.
328 * Differences significantly different from zero (p<0.05).
329

330

For peer review only

Table 3 describes the proportion of individuals with at least one incident or prevalent spell of part- or full-time SA or DP in the period T_0 to T_{+5}, stratified by MS, educational level, and type of occupation at T_0. The proportion of individuals with part- and full-time SA or DP was higher among PwMS than in corresponding group among the references at both time points. At T_0, 46.7% of the PwMS had at least one full-time SA spell and 10.0% had part-time SA. Among PwMS, the proportion of part- and full-time SA decreased with time, whereas the proportion of DP increased substantially. At the end of follow-up, 21.0% of the PwMS had at least one part-time DP spell, and the proportion of full-time DP was 25.1%. The patterns were similar for the reference group, but at lower levels at both time points than for the PwMS.

For PwMS, the proportions of part- and full-time SA at T_{+5} were somewhat similar for all levels of education (Table 3). Proportions by type of occupation varied more: managers and individuals with no work had lower proportions of part- and full-time SA than people in the other types of occupations. For PwMS at T_{+5}, the prevalence of individuals with part-time DP was higher than for full-time DP among those with university/college education and with more qualified types of occupations.

For peer review only

For peer review only - http://bmjopen.bmj.com/site/about/guidelines.xhtml

Table 3: Percentage of people with multiple sclerosis (PwMS) who had sickness absence[a] and disability pension [a] in the year of inclusion (T_0) and five years later (T_5), stratified by educational level and type of occupation at T_0.

Year	Factor	Category	Sickness absence				Disability pension			
			PwMS		Ref [c]		PwMS [c]		Ref [c]	
			Part-time	Full-time	Part-time	Full-time	Part-time	Full-time	Part-time	Full-time
**T_0**	**Education**	University/college	11.0	43.0	2.6	10.0	3.6	3.8	1.5	2.0
		High school	9.1	49.5	2.1	15.6	5.7	10.0	2.5	6.4
		Elementary school	11.0	45.6	1.3	16.3	8.4	22.8	3.5	16.9
**T_{+5}**		University/college	7.2	17.7	1.6	8.4	21.6	15.4	2.1	2.8
		High school	5.3	17.1	0.8	11.9	20.9	27.3	3.0	8.1
		Elementary school	6.6	13.2	0.8	10.0	19.7	48.3	4.9	19.6
**T_0**	**Occupation**	Managers	16.3	31.4	0.8	5.8	1.2	0.0	1.2	0.4
		Office	13.3	43.6	2.9	11.0	5.4	1.9	2.0	0.7
		Manual	8.5	55.7	2.1	17.2	5.1	3.0	2.7	1.4
		Unknown	10.0	43.9	2.3	17.1	12.3	13.1	2.3	7.7
		No Work[b]	2.1	37.2	0.3	15.0	3.2	49.3	2.4	39.8
**T_{+5}**		Managers	4.7	8.2	0.4	4.7	24.7	15.3	1.2	0.8
		Office	8.0	18.7	1.5	8.7	25.5	12.7	2.5	1.5
		Manual	5.4	19.1	0.8	13.6	20.2	23.7	3.5	2.9
		Unknown	7.9	13.4	1.7	11.3	24.2	33.6	2.9	10.4
		No Work[b]	1.9	8.5	0.8	6.8	6.3	69.7	3.2	45.5
**T_0**	**All**	-	**10.0**	**46.7**	**2.1**	**13.7**	**5.2**	**9.1**	**2.3**	**6.3**
**T_{+5}**		-	**6.2**	**16.9**	**1.1**	**10.4**	**21.0**	**25.1**	**2.9**	**7.9**

[a] Individuals with spell of sickness absence (SA) or disability pension (DP) with the highest grade (25, 50, 75 or 100%) at T_0 and T_{+5} (i.e., if an individual had both part- and full-time SA in a single year, the individual was categorised as full-time SA. The same procedure was employed for persons with DP).

[b] Individuals with unemployment benefits can be granted SA if the disease/injury is severe enough to prevent them from seeking employment. DP can be granted until the age of 65. In most cases, SA refers to compensation for a spell longer than the first 14 days, which are covered by the employer.

[c] Numbers at year T_0: PwMS (n=2553) and references (n=7584).

Row percent of all individuals (including those without SA or DP) in each sub-group.

For peer review only - http://bmjopen.bmj.com/site/about/guidelines.xhtml

Estimates from the Models 1 and 2 are presented in Table 4. In T_0, the estimates for MS (yes/no) showed similar negative relationships with earnings when comparing the regression that included educational level (T_0: -36.9, p=0.001) and that which included type of occupation (T_0: -33.3, p=0.001). The corresponding estimates for T_{+5} were also both negative; but with a larger difference between the estimates when including educational level (T_{+5}: -84.4, p=0.001) compared to type of occupation (T_{+5}: -49.9, p=0.001).

A hierarchic pattern in the estimates for educational level and type of occupation was observed. Higher levels of education (i.e., university/college) and more qualified occupations (i.e., managers and office workers) were associated with relatively higher levels of earnings compared to those with lower levels of education (i.e., high school and elementary school) or less qualified types of occupations.

For peer review only - http://bmjopen.bmj.com/site/about/guidelines.xhtml

Table 4: Cross-sectional Tobit regressions for earnings [a] at the year of inclusion [b] and five years later.

	**Education**		**Occupation**	
	**Model 1**		**Model 2**	
	**T_0** Beta estimate (95% CI)	**T_{+5}** Beta estimate (95% CI)	**T_0** Beta estimate (95% CI)	**T_{+5}** Beta estimate (95% CI)
Intercept	217.3 (208.3, 226.3)	285.8 (275.2, 296.4)	241.7 (234.6, 248.8)	279.4 (271.2, 287.6)
MS (ref: No MS) [c]	-36.9 (-43.0, -30.8)	-84.4 (-91.5, -77.3)	-33.3 (-38.0, -28.6)	-49.9 (-55.6, -44.2)
Male (ref: Female) [c]	68.1 (62.4, 73.8)	67.8 (61.1, 74.5)	63.4 (58.9, 67.9)	64.2 (58.9, 69.5)
Age (ref: 30-34 years) [c]				
- 35-39	17.5 (9.3, 25.7)	20.6 (11.0, 30.2)	9.6 (3.1, 16.1)	18.2 (10.8, 25.6)
- 40-44	35.5 (27.5, 43.5)	25.1 (15.7, 34.5)	25.7 (19.2, 32.2)	27.4 (20.0, 34.8)
- 45-49	33.4 (25.0, 41.8)	6.1 (-3.9, 16.1)	24.3 (17.6, 31.0)	16.6 (8.8, 24.4)
- 50-54	30.0 (21.6, 38.4)	-9.4 (-19.4, 0.6)	22.3 (15.6, 29.0)	9.6 (1.6, 17.6)
Country of birth (ref: Sweden) [c]	-78.4 (-86.0, -70.8)	-71.7 (-80.7, -62.7)	-20.3 (-26.6, -14.0)	-19.9 (-27.3, -12.5)
Panel (ref: 2006) [c]				
-2003	-5.1 (-12.5, 2.3)	-9.4 (-18.2, -0.6)	-6.9 (-12.8, -1.0)	-9.3 (-16.2, -2.4)
-2004	-5.7 (-13.3, 1.9)	-2.1 (-11.1, 6.9)	-7.3 (-13.4, -1.2)	-2.5 (-9.6, 4.6)
-2005	-7.8 (-15.4, -0.2)	-6.7 (-15.7, 2.3)	-6.0 (-12.1, 0.1)	-3.1 (-10.2, 4.0)
Type of living area (ref: Larger cities) [d]				
- Medium-sized municipalities	-19.9 (-26.0, -13.8)	-23.3 (-30.6, -16.0)	-20.8 (-25.7, -15.9)	-21.9 (-27.6, -16.2)
- Smaller municipalities	-26.1 (-32.8, -19.4)	-32.7 (-40.5, -24.9)	-29.5 (-34.8, -24.2)	-31.6 (-37.9, -25.3)
Educational level (ref: University/college) [c]				
- High school	-58.1 (-63.8, -52.4)	-79.7 (-86.4, -73.0)	-	-
- Elementary school	-113.2 (-121.8, -104.6)	-148.6 (-158.8, -138.4)	-	-
Type of occupation (ref: Office) [d]				
- Managers	-	-	63.5 (52.3, 74.7)	87.8 (76.8, 98.8)
- Manual	-	-	-65.6 (-70.1, -61.1)	-72.5 (-77.6, -67.4)
- Unknown	-	-	-135.9 (-145.5, -126.3)	-171.6 (-187.3, -155.9)
- No Work	-	-	-384.3 (-394.9, -373.7)	-519.7 (-538.9, -500.5)
Sigma	131.2 (129.6, 132.9)	152.4 (150.4, 154.4)	100.7 (99.4, 102.0)	113.8 (112.3, 115.3)
N	10,137	9,928	10,137	9,928
N censored at zero	1,26	1,505	1,26	1,505
Bayesian information criterion (BIC)	114,727	111,972	107,869	104,307

[a] Earnings: income from work and student allowances. Presented in 2005 values, in SEK 1000.
[b] Year of inclusion (T_0) for people with multiple sclerosis (PwMS): year of first MS diagnosis. Year of inclusion (T_0) for references: year of inclusion to one of four panels. Estimates from Tobit models with lower bound set at zero (i.e., no earnings).
[c] Time-invariant variables: measured at T_0.
[d] Time-variant variables: measured at T_0 or T_{+5}.

For peer review only

For peer review only - http://bmjopen.bmj.com/site/about/guidelines.xhtml

DISCUSSION

In this cohort study we identified 2553 PwMS of working ages and compared them with 7584 references without MS across time points for a period of 16 years. The principal findings were that both groups had similar levels of earnings and annual changes in earnings up until one year before the diagnosis year, thereafter, the growth in earnings levelled off among the PwMS for the remainder of the study period, while earnings for the references continued to increase. These differences remained when we controlled for a number of socio-demographic variables. The largest differences in absolute terms between PwMS and the references were observed for those with elementary school education and for those working as managers. Those with elementary school education also had the largest relative differences. There were also large within-group differences in the development of mean earnings for the PwMS with respect to educational level, and type of occupation. Further, the PwMS had higher rates of part- and full-time DP after the year of diagnosis compared to the references.

This study has several strengths, including identifying the PwMS from nationwide patient registers.[48] Further, the data from the other nationwide registers were also of high quality,[49] which made it possible to conduct our analyses with no loss to follow-up and without self-selection bias. One limitation is that onset of MS always precedes diagnosis, and this time lag varies considerably depending on the experienced symptoms, the individual's healthcare-seeking behaviour, healthcare practices, and timeliness to confirm diagnosis.[8 50] Therefore, the year of diagnosis used in this study can be seen as a biased proxy (although with known direction) for the year of MS onset. Without additional MS-specific data on clinical progression, we could not evaluate the degree to which this time lag affected the findings. The extent to which the time lag correlates with socio-demographic factors is, to the best of our knowledge, not known. Furthermore, educational level and type of occupation were

For peer review only

highly correlated, and therefore, analysed separately. Hence, when we analysed one of the variables, the effect of the other variable could, to some degree, be incorporated indirectly. The generalisability of the findings may be limited to settings with a similarly functioning welfare system and labour market. Finally, due to data limitations, our definition of earnings includes shorter spells of SA (days 2-14), which may conceal the initial effects of MS, and underestimate the potential effect of MS on earnings for individuals with repeated short spells of SA.

Our overall results are in line with previous studies, which have shown non-increasing gross income,[13 15] and high levels of unemployment or SA/DP among PwMS.[10 11 14 32 34 38 51-53] We found that differentiating the analyses by either educational level or type of occupation overall gave similar results. However, the combination of occupation and annual earnings provided additional information, including information on those who were working compared with those not in paid work. Further, comparing the PwMS' earnings with their peers in the occupational reference groups showed that PwMS classified as managers had larger differences in earnings than those in office work. However, it is possible that the large aggregates chosen for categorisation of occupation disguised within-group variation in terms of the physical demands of work tasks. Likewise, the lack of differentiation within the university/college educational category could have concealed strong heterogeneity.

We found considerable heterogeneity within the group of PwMS; while a large proportion appeared to participate on the labour market to a high extent, many had low or no earnings at all. There are many possible explanations of this heterogeneity, with the individuals' educational level and type of occupation as two influential factors within this study. This suggests that the PwMS' work situations are important determinants of subsequent labour market outcomes. We also observed that PwMS with university/college education or with more qualified types of occupations (i.e., office workers or managers) had higher proportions

For peer review only - http://bmjopen.bmj.com/site/about/guidelines.xhtml

of part-time DP (rather than full-time DP) than those with lower education levels or less qualified types of work (i.e, manual workers or those with no work). This could potentially reflect the nature of physical work demands among manual occupations, or reflect a lower degree of flexibility to adjust one's work situation than among non-manual occupations, which may lead to higher rates of full-time rather than part-time DP. It was not possible to capture this distinction in the present study. However, it is also possible that our variables, educational level and type of occupation, were proxies for less tangible individual factors. One such factor could be healthcare seeking behaviour, where those with a higher educational level or more qualified occupations may receive the MS diagnosis, and thus treatment, earlier in the disease trajectory. Nevertheless, MS affected an individual's work capacity regardless of whether a diagnosis had been set or not, the new generation of disease modifying treatments to slow disease progression, and thus preserve work capacity, were first introduced in 2006, but not administered to the majority of PwMS until years later.[54] Therefore, it is possible that this could partly explain the observed differences in earnings between the groups. Similarly, the wide distribution of earnings among PwMS could reflect the known heterogeneous progression of the disease - irrespective of educational level or type of occupation.

A possible explanation of the observed increasing differences in earnings between PwMS and individuals without MS, in addition to working less than full-time, and situations of pay for performance, could be due to different opportunities for mobility in the labour market. An individual's wage development is generally associated with possibilities for changing employers. This is especially so in Sweden, with largely centrally negotiated wage increases, with often limited individual performance variation.[55 56] Thus, labour market mobility provides an opportunity for substantial wage improvement, and a MS diagnosis may deter changing employment.[57] More studies of PwMS' or more generally, on chronically ill

For peer review only - http://bmjopen.bmj.com/site/about/guidelines.xhtml

individuals' wages and labour market mobility are needed to increase our knowledge about such mechanisms, e.g., by studying transitions between different occupations, types of employment, and unemployment.[58 59]

Furthermore, the large differences in earnings among individuals with educational levels less than university/college following MS diagnosis compared to the reference group, provides support for the conclusion that the degree of physical labour and/or flexibility at the work place is of importance for remaining in paid work once diagnosed with MS. However, further research is required to explain the observed differences between PwMS and the references in managerial positions.

Lastly, health outcomes research is limited by a lack of measures reflecting the multitude of MS consequences.[60] The traditional MS-specific health outcome measurement tool, Expanded Disability Systems Score (EDSS), typically overemphasises the importance of motor function and poorly reflects cognitive functions and fatigue,[60] two factors strongly influencing work capacity.[18 60-62] Our results suggest that earnings could potentially be a promising outcome measure assessing a composite of functions.[18] The underlying assumption is that the reduction of earnings among PwMS to a great extent reflects MS-related disability. The observed impact of MS on earnings among PwMS across occupational types and educational levels indicates strongly that motor function, which is known to deteriorate slowly, at least in younger patients, is not the sole reason for the decrease in earnings. Thus, indicating the possible influence of fatigue and cognition.[18 61] Furthermore, our findings of earnings development reflect the same trends as the pattern of disability development, as assessed by EDSS, suggesting that earnings may serve as a surrogate outcome measure reflecting the broader consequences of MS.[18] Further studies are, therefore, required with data sets containing both earnings and EDSS scores to compare the two in terms of resolution and validity. Earnings, when available from public registers, such as from tax authorities,

For peer review only - http://bmjopen.bmj.com/site/about/guidelines.xhtml

have the additional benefits of minimal data loss and reflecting comprehensive time periods, whereas EDSS outcomes reflect single points in time.[18]

CONCLUSIONS

From one year prior to the MS diagnosis and onwards, we observed increasingly higher levels of mean earnings in the reference group compared to in the PwMS. Further, we observed within-group heterogeneity among the PwMS; after five years, a large percentage still participated in the labour market, nevertheless, many had low or no earnings at all. We also found that those with at most elementary or secondary school levels of education or less qualified types of occupations were less alike their respective peers in the reference group than those with university/college levels of education and more qualified types of occupations. Moreover, part- or full-time usage of the sickness insurance was more common among the PwMS – a result that could potentially be used to describe the observed heterogeneity of earnings among the PwMS.

For peer review only

List of abbreviations used

CI: Confidence intervals.

DP: Disability pension.

EDSS: The expanded disability systems score.

IQR: Interquartile range.

MS: Multiple sclerosis.

OLS: Ordinary least squares.

PwMS: People with multiple sclerosis.

SA: Sickness absence.

SEK: Swedish Krona.

For peer review only

For peer review only - http://bmjopen.bmj.com/site/about/guidelines.xhtml

Declarations

Ethics approval

The project was approved by the Regional Ethical Review Board, Stockholm, Sweden.

Acknowledgements

Not applicable

Authors' contributions

All authors participated in the design of the study. MW performed the data analyses. All authors contributed to the interpretations of the results and participated in the writing of the manuscript.

Competing interests statement

MW, CM, PT and EF were partly funded by Biogen. ED and MS have no competing interests to declare. KA has received unrestricted grants from Biogen. JH received honoraria for serving on advisory boards for Biogen and Novartis and speaker's fees from Biogen, Merck-Serono, Bayer-Schering, Teva and Sanofi-Aventis. He has served as P.I. for projects sponsored by, or received unrestricted research support from, Biogen, Merck-Serono, TEVA, Novartis, and Bayer-Schering. His MS research is funded by the Swedish Research Council.

Funding

The project was funded by unrestricted grants from Biogen and the Swedish Research Council for Health, Working Life and Welfare. The design of the study, data collection, analyses, interpretations of data, and writing of manuscript was performed without involvement of the funding bodies. Biogen was given the opportunity to comment on the manuscript before submission.

Data statement

Not applicable. The data cannot be made publically available due to privacy regulations. According to the General Data Protection Regulation, the Swedish, the Swedish Data Protection Act, the Swedish Ethical Review Act, and the Public Access to Information and Secrecy Act, data can only be made available for specific purposes, including research that meets the criteria for access to this type of sensitive and confidential data as determined by a legal review. Readers may contact Professor Kristina Alexanderson (kristina.alexanderson@ki.se) regarding the data.

For peer review only

For peer review only - http://bmjopen.bmj.com/site/about/guidelines.xhtml

LEGENDS

Figure One

Figure 1: Distribution of earnings in the total study population, at year of inclusion (n=10,137) and five years later (n=9928), in 1000 Swedish Krona (SEK).

Earnings: Combined income from work and student allowance, calculated in 2005 monetary values. The years of inclusion (T_0) vary between 2003-2006. They are for PwMS the year of the first MS diagnosis and for references the year of inclusion into one of the four panels.

Figure Two

Figure 2: Distribution (box and whisker plots) of earnings by time, for PwMS (n=2553) and matched references (n=7584), in 1000 Swedish Krona (SEK).

Earnings: Combined income from work and student allowance, calculated in 2005 monetary values. The years of inclusion (T_0) vary between 2003-2006. They are for PwMS the year of the first MS diagnosis and for references the year of inclusion into one of the four panels. Observations that were larger than 1.5*IQR (Interquartile Range) are circled. Symbols in the box plot: bar = median earnings, diamond = mean earnings. Line across box-plots = median earnings for the reference group at T_0 (SEK 204,850).

For peer review only

For peer review only - http://bmjopen.bmj.com/site/about/guidelines.xhtml

References

1. Tullman MJ. A review of current and emerging therapeutic strategies in multiple sclerosis. *Am J Manag Care* 2013;19(2 Suppl):S21-7.
2. Naci H, Fleurence R, Birt J, et al. Economic burden of multiple sclerosis. *Pharmacoeconomics* 2010;28(5):363-79.
3. Pugliatti M, Rosati G, Carton H, et al. The epidemiology of multiple sclerosis in Europe. *Eur J Neurol* 2006;13(7):700-22.
4. Chruzander C, Tinghög P, Ytterberg C, et al. Longitudinal changes in sickness absence and disability pension, and associations between disability pension and disease-specific and contextual factors and functioning, in people with multiple sclerosis. *J Neurol Sci* 2016
5. Ernstsson O, Gyllensten H, Alexanderson K, et al. Cost of Illness of Multiple Sclerosis-A Systematic Review. *PLoS One* 2016;11(7):e0159129.
6. Gyllensten H, Wiberg M, Alexanderson K, et al. Costs of illness of multiple sclerosis in Sweden: a population-based register study of people of working age. *Eur J Health Econ* 2018;19(3):435-46.
7. Gyllensten H, Wiberg M, Alexanderson K, et al. Comparing costs of illness of multiple sclerosis in three different years: A population-based study. *Mult Scler* 2018;24(4):520-28.
8. Brundin L, Kobelt G, Berg J, et al. New insights into the burden and costs of multiple sclerosis in Europe: Results for Sweden. *Mult Scler* 2017;23:179-91.
9. Green G, Todd J, Pevalin D. Biographical disruption associated with multiple sclerosis: using propensity scoring to assess the impact. *Soc Sci Med* 2007;65(3):524-35.
10. Wiberg M, Friberg E, Stenbeck M, et al. Sources and level of income among individuals with multiple sclerosis compared to the general population: A nationwide population-based study. *Multiple Sclerosis Journal* 2015;21(13):1730-41.
11. Jennum P, Wanscher B, Frederiksen J, et al. The socioeconomic consequences of multiple sclerosis: a controlled national study. *Eur Neuropsychopharmacol* 2012;22(1):36-43.
12. Krause I, Kern S, Horntrich A, et al. Employment status in multiple sclerosis: impact of disease-specific and non-disease-specific factors. *Mult Scler* 2013;19(13):1792–99.
13. Pfleger CCH, Flachs EM, Koch-Henriksen N. Social consequences of multiple sclerosis (1): early pension and temporary unemployment—a historical prospective cohort study. *Mult Scler* 2010;16(1):121-26.
14. Pearson J, Alla S, Clarke S, et al. Multiple Sclerosis impact on employment and income in New Zealand. *Acta Neurol Scand* 2017;136(3):223–32.
15. Landfeldt E, Castelo-Branco A, Svedbom A, et al. Personal Income Before and After Diagnosis of Multiple Sclerosis. *Value Health* 2018;21(5):590-95.
16. Murley C, Mogard O, Wiberg M, et al. Trajectories of disposable income among people of working ages diagnosed with multiple sclerosis: a nationwide register-based cohort study in Sweden 7 years before to 4 years after diagnosis with a population-based reference group. *BMJ Open* 2018;8(5):e020392.
17. Thormann A, Sørensen P, Koch-Henriksen N, et al. Chronic comorbidity in multiple sclerosis is associated with lower incomes and dissolved intimate relationships. *Eur J Neurol* 2017;24(6):825-34. doi: 10.1111/ene.13297
18. Kavaliunas A, Wiberg M, Tinghog P, et al. Earnings and Financial Compensation from Social Security Systems Correlate Strongly with Disability for Multiple Sclerosis Patients. *PLoS One* 2015;10(12):e0145435.
19. Abegunde DO, Stanciole AE. The economic impact of chronic diseases: how do households respond to shocks? Evidence from Russia. *Soc Sci Med* 2008;66(11):2296-307.
20. Carlsson KS, Landin-Olsson M, Nyström L, et al. Long-term detrimental consequences of the onset of type 1 diabetes on annual earnings—evidence from annual registry data in 1990–2005. *Diabetologia* 2010;53(6):1084-92.
21. Løkke A, Hilberg O, Kjellberg J, et al. Economic and Health Consequences of COPD Patients and Their Spouses in Denmark-1998-2010. *COPD* 2014;11(3):237-46.

For peer review only - http://bmjopen.bmj.com/site/about/guidelines.xhtml

22. Mitchell JM, Burkhauser RV, Pincus T. The importance of age, education, and comorbidity in the substantial earnings losses of individuals with symmetric polyarthritis. *Arthritis Rheum* 1988;31(3):348-57.
23. Shanahan EM, Smith MD, Roberts-Thomson L, et al. The effect of rheumatoid arthritis on personal income in Australia. *Intern Med J* 2008;38(7):575-79.
24. Ward MM, Javitz HS, Smith WM, et al. Lost income and work limitations in persons with chronic respiratory disorders. *J Clin Epidemiol* 2002;55(3):260-68.
25. Wolfe F, Michaud K, Choi HK, et al. Household income and earnings losses among 6,396 persons with rheumatoid arthritis. *J Rheumatol* 2005;32(10):1875-83.
26. Confavreux C, Vukusic S, Adeleine P. Early clinical predictors and progression of irreversible disability in multiple sclerosis: an amnesic process. *Brain* 2003;126(Pt 4):770-82.
27. Ruggieri R, Palermo R, Vitello G, et al. Cognitive impairment in patients suffering from relapsing-remitting multiple sclerosis with EDSS≤ 3.5. *Acta Neurol Scand* 2003;108(5):323-26.
28. Degenhardt A, Ramagopalan SV, Scalfari A, et al. Clinical prognostic factors in multiple sclerosis: a natural history review. *Nat Rev Neurol* 2009;5(12):672-82.
29. Langer-Gould A, Popat RA, Huang SM, et al. Clinical and demographic predictors of long-term disability in patients with relapsing-remitting multiple sclerosis: a systematic review. *Arch Neurol* 2006;63(12):1686-91.
30. Confavreux C, Vukusic S. Age at disability milestones in multiple sclerosis. *Brain* 2006;129(Pt 3):595-605. doi: 10.1093/brain/awh714
31. Beatty WW, Blanco CR, Wilbanks SL, et al. Demographic, clinical, and cognitive characteristics of multiple sclerosis patients who continue to work. *Neurorehabil Neural Repair* 1995;9(3):167-73.
32. Busche K, Fisk J, Murray T, et al. Short Term Predictors of Unemployment in Multiple Sclerosis Patients. *Can J Neurol Sci* 2003;30(2):137-42. doi: doi.org/10.1017/S0317167100053403
33. Grønning M, Hannisdal E, Mellgren SI. Multivariate analyses of factors associated with unemployment in people with multiple sclerosis. *J Neurol Neurosurg Psychiatry* 1990;53(5):388-90.
34. Lunde HMB, Telstad W, Grytten N, et al. Employment among Patients with Multiple Sclerosis-A Population Study. *PLoS One* 2014;9(7):e103317.
35. Patti F, Pozzilli C, Montanari E, et al. Effects of education level and employment status on HRQoL in early relapsing-remitting multiple sclerosis. *Mult Scler* 2007;13(6):783-91.
36. Roessler RT, Rumrill PD, Fitzgerald SM. Predictors of employment status for people with multiple sclerosis. *Rehabil Couns Bull* 2004;47(2):96-103.
37. Smith MM, Arnett PA. Factors related to employment status changes in individuals with multiple sclerosis. *Mult Scler* 2005;11(5):602-09.
38. Glad SB, Nyland H, Aarseth JH, et al. How long can you keep working with benign multiple sclerosis? *J Neurol Neurosurg Psychiatry* 2011;82(1):78-82.
39. Moore P, Harding KE, Clarkson H, et al. Demographic and clinical factors associated with changes in employment in multiple sclerosis. *Mult Scler* 2013:1352458513481396.
40. Findling O, Baltisberger M, Jung S, et al. Variables Related to Working Capability among Swiss Patients with Multiple Sclerosis—A Cohort Study. *PLoS One* 2015
41. Social Insurance in Figures 2014. Stockholm, Sweden, 2014.
42. Background Facts, Labour and Education Statistics 2011:4, Integrated database for labour market research. Stockholm: Statistics Sweden, 2011.
43. Rikets indelningar: årsbok över regionala indelningar med koder, postadresser, telefonnummer [Country classifications: yearbook of regional classifications with codes, postal addresses, phone numbers 2003]. Stockholm: Statistics Sweden, 2003.
44. SSYK 96: Standard för svensk yrkesklassificering 1996 [SSYK 96: Standard for Swedish occupation classification 1996]. Swedish Standard Classification of Occupations. Örebro: Statistics Sweden, 1998.
45. Wooldridge JM. Corner solution outocmes and censored regression models. Econometric analysis of cross section and panel data. Cambridge, Massachusetts: MIT Press 2002.

For peer review only - http://bmjopen.bmj.com/site/about/guidelines.xhtml

46. Tobin J. Estimation of relationships for limited dependent variables. *Econometrica* 1958;26(1):24-36.
47. Humphreys BR. Dealing with zeros in economic data. University of Alberta, Alberta: Department of Economics, 2013.
48. Ludvigsson JF, Andersson E, Ekbom A, et al. External review and validation of the Swedish national inpatient register. *BMC Public Health* 2011;11(1):450.
49. Ludvigsson JF, Almqvist C, Bonamy A-KE, et al. Registers of the Swedish total population and their use in medical research. *Eur J Epidemiol* 2016;31(2):125-36.
50. Thompson AJ, Banwell BL, Barkhof F, et al. Diagnosis of multiple sclerosis: 2017 revisions of the McDonald criteria. *Lancet Neurol* 2018;17(2):162-73.
51. Glad S, Aarseth J, Nyland H, et al. Benign multiple sclerosis: a need for a consensus. *Acta Neurol Scand* 2010;122(s190):44-50.
52. Julian LJ, Vella L, Vollmer T, et al. Employment in multiple sclerosis. *J Neurol* 2008;255(9):1354-60.
53. Simmons RD, Tribe KL, McDonald EA. Living with multiple sclerosis: longitudinal changes in employment and the importance of symptom management. *J Neurol* 2010;257(6):926-36.
54. Eriksson I, Komen J, Piehl F, et al. The changing multiple sclerosis treatment landscape: impact of new drugs and treatment recommendations. *Eur J Clin Pharmacol* 2018;74(5):663-70.
55. Nyberg A, Magnusson Hanson LL, Leineweber C, et al. Do Predictors of Career Success Differ between Swedish Women and Men? Data from the Swedish Longitudinal Occupational Survey of Health (SLOSH). *PLoS One* 2015;10(10):e0140516.
56. Muffels R, Luijkx R. Labour market mobility and employment security of male employees in Europe:trade-off'orflexicurity'? *Work, employment and Society* 2008;22(2):221-42.
57. McAllister A, Nylen L, Backhans M, et al. Do 'flexicurity' Policies Work for People With Low Education and Health Problems? A Comparison of Labour Market Policies and Employment Rates in Denmark, The Netherlands, Sweden, and the United Kingdom 1990-2010. *Int J Health Serv* 2015;45(4):679-705.
58. Ekberg K, Wahlin C, Persson J, et al. Is mobility in the labor market a solution to sustainable return to work for some sick listed persons? *Journal of occupational rehabilitation* 2011;21(3):355-65.
59. Nordstrom K, Ekberg K, Hemmingsson T, et al. Sick leave and the impact of job-to-job mobility on the likelihood of remaining on the labour market--a longitudinal Swedish register study. *BMC Public Health* 2014;14:305.
60. van Munster CEP, Uitdehaag BMJ. Outcome Measures in Clinical Trials for Multiple Sclerosis. *CNS Drugs* 2017;31(3):217-36. 5
61. Kavaliunas A, Karrenbauer VD, Tinghog P, et al. Cognitive function predicts work disability among multiple sclerosis patients. *Mult Scler* 2017;23:479-80.
62. Kavaliunas A, Karrenbauer VD, Gyllensten H, et al. Cognitive function is a major determinant of income among multiple sclerosis patients in Sweden acting independently from physical disability. *Mult Scler* 2017:1352458517740212.

For peer review only - http://bmjopen.bmj.com/site/about/guidelines.xhtml

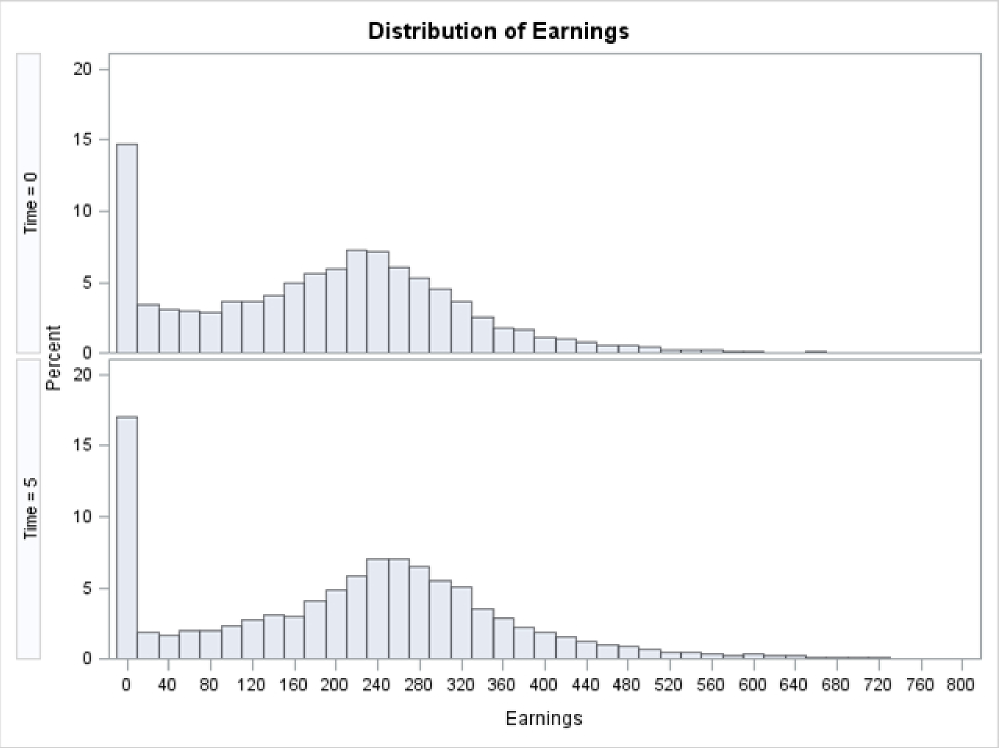

Distribution of earnings in the total study population, at year of inclusion (n=10,137) and five years later (n=9928), in 1000 Swedish Krona (SEK).Earnings: Combined income from work and student allowance, calculated in 2005 monetary values. The years of inclusion (T0) vary between 2003-2006. They are for PwMS the year of the first MS diagnosis and for references the year of inclusion into one of the four panels.

54x40mm (300 x 300 DPI)

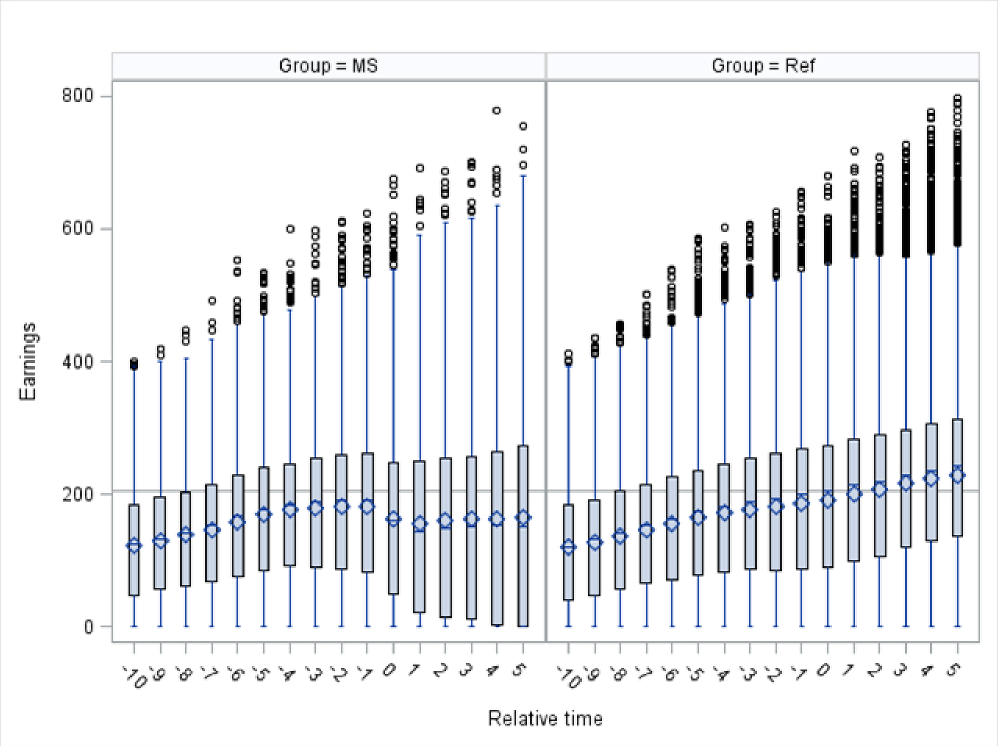

Distribution (box and whisker plots) of earnings by time, for PwMS (n=2553) and matched references (n=7584), in 1000 Swedish Krona (SEK). Earnings: Combined income from work and student allowance, calculated in 2005 monetary values. The years of inclusion (T0) vary between 2003-2006. They are for PwMS the year of the first MS diagnosis and for references the year of inclusion into one of the four panels. Observations that were larger than 1.5*IQR (Interquartile Range) are circled. Symbols in the box plot: bar = median earnings, diamond = mean earnings. Line across box-plots = median earnings for the reference group at T0 (SEK 204,850).

54x40mm (300 x 300 DPI)

STROBE 2007 (v4) checklist of items to be included in reports of observational studies in epidemiology*
Checklist for cohort, case-control, and cross-sectional studies (combined)

Section/Topic	Item #	Recommendation	Reported on page #
**Title and abstract**	1	(*a*) Indicate the study's design with a commonly used term in the title or the abstract	1 (title)<br>2 (abstract)
		(*b*) Provide in the abstract an informative and balanced summary of what was done and what was found	2-3
**Introduction**			
Background/rationale	2	Explain the scientific background and rationale for the investigation being reported	5-6
Objectives	3	State specific objectives, including any pre-specified hypotheses	6-7 (in the form of study aims)
**Methods**			
Study design	4	Present key elements of study design early in the paper	7 first paragraph of Methods
Setting	5	Describe the setting, locations, and relevant dates, including periods of recruitment, exposure, follow-up, and data collection	7-8
Participants	6	(*a*) *Cohort study*—Give the eligibility criteria, and the sources and methods of selection of participants. Describe methods of follow-up<br>*Case-control study*—Give the eligibility criteria, and the sources and methods of case ascertainment and control selection. Give the rationale for the choice of cases and controls<br>*Cross-sectional study*—Give the eligibility criteria, and the sources and methods of selection of participants	Cohort study<br>7-10.
		(*b*) *Cohort study*—For matched studies, give matching criteria and number of exposed and unexposed<br>*Case-control study*—For matched studies, give matching criteria and the number of controls per case	
Variables	7	Clearly define all outcomes, exposures, predictors, potential confounders, and effect modifiers. Give diagnostic criteria, if applicable	8- outcome variable<br>8-9 explanatory variables
Data sources/ measurement	8*	For each variable of interest, give sources of data and details of methods of assessment (measurement). Describe comparability of assessment methods if there is more than one group	8- source<br>8-9 measurement and classification of variables
Bias	9	Describe any efforts to address potential sources of bias	12
Study size	10	Explain how the study size was arrived at	7-8

For peer review only

Quantitative variables	11	Explain how quantitative variables were handled in the analyses. If applicable, describe which groupings were chosen and why	11-12
Statistical methods	12	(*a*) Describe all statistical methods, including those used to control for confounding	11-12
		(*b*) Describe any methods used to examine subgroups and interactions	12 – by education and occupation
		(*c*) Explain how missing data were addressed	10
		(*d*) *Cohort study*—If applicable, explain how loss to follow-up was addressed<br>*Case-control study*—If applicable, explain how matching of cases and controls was addressed<br>*Cross-sectional study*—If applicable, describe analytical methods taking account of sampling strategy	10
		(*e*) Describe any sensitivity analyses	NA
**Results**			
Participants	13*	(a) Report numbers of individuals at each stage of study—eg numbers potentially eligible, examined for eligibility, confirmed eligible, included in the study, completing follow-up, and analysed	10
		(b) Give reasons for non-participation at each stage	10
		(c) Consider use of a flow diagram	NA
Descriptive data	14*	(a) Give characteristics of study participants (eg demographic, clinical, social) and information on exposures and potential confounders	13 and Table 1
		(b) Indicate number of participants with missing data for each variable of interest	10/ NA
		(c) *Cohort study*—Summarise follow-up time (eg, average and total amount)	-
Outcome data	15*	*Cohort study*—Report numbers of outcome events or summary measures over time	15-19
		Case-control study—Report numbers in each exposure category, or summary measures of exposure	
		Cross-sectional study—Report numbers of outcome events or summary measures	
Main results	16	(*a*) Give unadjusted estimates and, if applicable, confounder-adjusted estimates and their precision (eg, 95% confidence interval). Make clear which confounders were adjusted for and why they were included	20-21
		(*b*) Report category boundaries when continuous variables were categorized	NA
		(*c*) If relevant, consider translating estimates of relative risk into absolute risk for a meaningful time period	
Other analyses	17	Report other analyses done—eg analyses of subgroups and interactions, and sensitivity analyses	
**Discussion**			
Key results	18	Summarise key results with reference to study objectives	22
Limitations	19	Discuss limitations of the study, taking into account sources of potential bias or imprecision. Discuss both direction and magnitude of any potential bias	22-23
Interpretation	20	Give a cautious overall interpretation of results considering objectives, limitations, multiplicity of analyses, results from similar studies, and other relevant evidence	23-25

For peer review only

Generalisability	21	Discuss the generalisability (external validity) of the study results	23
**Other information**			
Funding	22	Give the source of funding and the role of the funders for the present study and, if applicable, for the original study on which the present article is based	28

*Give information separately for cases and controls in case-control studies and, if applicable, for exposed and unexposed groups in cohort and cross-sectional studies.
Note: An Explanation and Elaboration article discusses each checklist item and gives methodological background and published examples of transparent reporting. The STROBE checklist is best used in conjunction with this article (freely available on the Web sites of PLoS Medicine at http://www.plosmedicine.org/, Annals of Internal Medicine at http://www.annals.org/, and Epidemiology at http://www.epidem.com/). Information on the STROBE Initiative is available at www.strobe-statement.org.

For peer review only

BMJ Open

Earnings among people with multiple sclerosis compared to references, in total and by educational level and type of occupation: a population-based cohort study at different points in time

Journal:	*BMJ Open*
Manuscript ID	bmjopen-2018-024836.R1
Article Type:	Research
Date Submitted by the Author:	08-Feb-2019
Complete List of Authors:	Wiberg, Michael; Karolinska Institutet Department of Clinical Neuroscience, Division of Insurance Medicine; Swedish Social Insurance Agency, Department of Analysis and Forecast<br>Murley, Chantelle; Karolinska Institutet Department of Clinical Neuroscience, Division of Insurance Medicine<br>Tinghög, Petter; Karolinska Institutet Department of Clinical Neuroscience, Division of Insurance Medicine; Red Cross University College<br>Alexanderson, Kristina; Karolinska Institutet Department of Clinical Neuroscience, Division of Insurance Medicine<br>Palmer, Edward; Uppsala University , Department of Economics, Uppsala Center for Labor Studies<br>Hillert, Jan; Karolinska Institutet Department of Clinical Neuroscience, Division of Neurology<br>Stenbeck, Magnus; Karolinska Institutet Department of Clinical Neuroscience, Division of Insurance Medicine<br>Friberg, Emilie; Karolinska Institutet Department of Clinical Neuroscience, Division of Insurance Medicine
**Primary Subject Heading**:	Neurology
Secondary Subject Heading:	Epidemiology
Keywords:	Multiple sclerosis < NEUROLOGY, Earnings, Sick leave, Disability pension, Work, Social Insurance

SCHOLARONE™
Manuscripts

Earnings among people with multiple sclerosis compared to references, in total and by educational level and type of occupation: a population-based cohort study at different points in time.

Michael Wiberg[1,2], Chantelle Murley[1], Petter Tinghög[1,3], Kristina Alexanderson[1], Edward Palmer[4], Jan Hillert[5], Magnus Stenbeck[1] and Emilie Friberg[1].

[1] Department of Clinical Neuroscience, Division of Insurance Medicine, Karolinska Institutet, SE 171 77 Stockholm, Sweden.

[2] Department of Analysis and Forecast, Swedish Social Insurance Agency, 103 51 Stockholm, Sweden.

[3] Department of Health Sciences, Swedish Red Cross University College, 141 21 Huddinge, Sweden.

[4] Uppsala Department of Economics and Center for Labor Studies, 753 13 Uppsala, Sweden.

[5] Department of Clinical Neuroscience, Division of Neurology, Karolinska Institutet, SE 171 77 Stockholm, Sweden.

Corresponding author: Chantelle Murley

Phone +46(0)8524 832 24

Fax +46(0)8524 832 05

wiberg.michael@gmail.com, chantelle.murley@ki.se, Petter.Tinghog@rkh.se, Kristina.Alexanderson@ki.se, Edward.E.Palmer@gmail.com, Jan.Hillert@ki.se, Magnus.Stenbeck@ki.se, Emilie.Friberg@ki.se.

Keywords (6): MS, Earnings, Sick leave, Disability pension, Work, Social insurance.

Word counts:

Abstract: 294 (max 300)

Main text excluding title page, abstract, references, figures and tables: 4767

Number of references: 62.

Number of tables: 4.

Number of figures: 2.

For peer review only

For peer review only - http://bmjopen.bmj.com/site/about/guidelines.xhtml

ABSTRACT

Objectives

To investigate the mean levels and distributions of earnings among people with multiple sclerosis (PwMS) before and after MS diagnosis compared to people without MS, and if they were associated with different educational levels and types of occupations. Furthermore, to assess the prevalence of sickness absence (SA) and disability pension (DP) in the two groups.

Design

Population-based longitudinal cohort study (10 before until 5 years after MS diagnosis).

Setting

Working-age population using microdata linked from nationwide Swedish registers.

Participants

Residents in Sweden in 2004 aged 30-54 with MS diagnosed in 2003-2006 (n=2553), and references without MS (n=7584) randomly selected by stratified matching (sex and age).

Outcome measures

Quartiles of earnings were calculated for each year prior to and following the MS diagnosis. Mean earnings, by educational level and type of occupation, before and after diagnosis were compared using t-tests. Tobit regressions investigated the associations of earnings with individual characteristics.

Results

Differences in earnings between PwMS and the references were observed beginning one year prior to diagnosis, with differences increasing with time. PwMS had lower mean earnings for

For peer review only - http://bmjopen.bmj.com/site/about/guidelines.xhtml

the diagnosis year (difference= SEK 28,000, p-value <0.05), and five years after diagnosis the difference had more than doubled (p-value <0.05). These differences remained after including educational level and type of occupation. Overall, the earnings of PwMS with university education and/or more qualified occupations were most like their respective reference peers. The prevalence of SA and DP were higher for PwMS than for the references.

Conclusions

The results suggest that the earnings among working-aged PwMS are lower than the references' from shortly before diagnosis, with this gap increasing thereafter. Besides full- and part-time SA and DP, the results indicate that educational level and type of occupation are influential factors behind the large heterogeneity in PwMS' earnings.

For peer review only

ARTICLE SUMMARY

Strengths and limitations:

- The main strengths of this study include the population-based longitudinal cohort design, use of high-quality nationwide registers and a large cohort of people with newly registered MS diagnoses which enabled analyses of earnings on a nationwide scale covering 16 years per individual and comparisons with a matched reference group.
- Potential selection bias was limited by using information about all resident in Sweden regarding both the PwMS and reference group, a feature not often the case for these types of studies.
- Extensive annual data on socio-demographics, occupation, and sickness absence and disability pension could be included for ten consecutive years before the year of MS diagnosis and five years after, to further understand work attachment of PwMS.
- A weakness was that earnings included the sick pay for employees with short-term sickness absence spells (≤14 days) which might underestimate the effect of MS on earnings if PwMS have a higher frequency of such spells than the reference group, as lost earnings due to sickness absence are not fully compensated in Sweden.
- Another weakness was that we only had information on the diagnosis date rather than on the onset date, however, it is unknown whether there are systematic differences within the MS cohort regarding the time between onset and diagnosis.

For peer review only

BACKGROUND

Multiple sclerosis (MS) is a chronic, often progressive, disease that usually onsets in working ages.[1] The combination of the relatively early onset age and chronic nature may affect the individual's economic welfare for a substantial part of their working life, through reduced productivity (and wage) per unit of time worked and/or the number of hours worked [2-6]. Furthermore, MS poses a high cost to society in terms of both direct and indirect costs (from the productivity losses).[2 5 7-11] Previous studies have predominantly focused on societal cost burdens, whereas the effects of MS on the development of individuals' earnings, including possible differences by educational level and type of occupation have not been extensively researched.

Several studies have found that people with MS (hereafter "PwMS") to a greater extent are not in paid work compared to individuals without MS.[3 4 6 12-14] However, sorting individuals into conventional labour market categories (e.g., employed/unemployed) obscures more nuanced information about the gradual effect of the disease on the individuals' labour market attachment.

An alternative measure for labour market attachment is the development of individuals' earnings. There are a growing number of studies (including MS-specific[3 6 14-18]) showing negative associations between chronic diseases and individuals' earnings.[3 6 13-25]

The progression of MS is heterogeneous.[26 27] Accordingly, it is difficult to predict disease progression at the individual level.[28] Age at onset of symptoms is one of the few socio-demographic factors that have been shown to be predictive of disease course.[29 30] Further, younger age at onset has also been associated with current employment.[4 31-34] In addition, educational level has been shown to influence labour market participation among PwMS.[34-38]

Therefore, educational level may also suggest the degree of physical labour and/or flexibility at the workplace.[34 36-38]

In working-aged individuals, educational level and type of occupation are highly correlated (e.g., university education increases possibilities for more qualified occupations). However, this does not necessarily hold for individuals with chronic diseases, as the disease might hinder them from performing the work corresponding to their educational level. Furthermore, one's educational level may remain unchanged above a certain age whereas an individual's occupation can vary over time, both due to individual and macro-economic factors. For this reason, in order to analyse the effects of a progressively disabling disease on work, the actual work performed (i.e., earnings), may be more informative than the individual's educational level.

Furthermore, PwMS to a high extent work part-time.[2 37 39 40] This is especially relevant for Sweden, where compensation for sickness absence (SA) and disability pension (DP) can be for either part- or full-time absences from ordinary working hours. Increasing utilisation of these compensation systems, which do not fully compensate the lost earnings, would over time result in increasing differences in annual earnings between PwMS and other wage earners, with all other factors equal.[15] Studies of such associations comparing different time points are still very few and the possible differences by the individuals' educational level and the type of work remain largely unexplored. The present study aims at improving our knowledge in this area.

The aims of this study were three-fold:

1. To examine levels and distributions of earnings of PwMS compared to matched references from the general population, during the years prior to and after the year of diagnosis with MS;

For peer review only - http://bmjopen.bmj.com/site/about/guidelines.xhtml

2. To explore if the level of education and the type of occupation correlate with the within group differences in earnings among PwMS; and
3. To compare the rates of part- or full-time SA and DP benefits between PwMS and the matched references.

METHODS

A population-based longitudinal cohort study was conducted using information from Statistics Sweden on all individuals living in Sweden on 31 December 2004. The study population was attained in two steps.

Firstly, we identified all individuals aged 30-54 years, who in 2003-2006 had their first registered MS diagnosis (ICD-9: 340, ICD-10: G35). Information on MS diagnoses was obtained from the following nationwide registers: inpatient hospital healthcare (data from 1987-2006), specialised outpatient healthcare (2001-2006), and outpatient surgery (1997-2000) from the National Patient Register maintained by the National Board of Health and Welfare, and days compensated by the Social Insurance Agency of SA (2005-2006), and DP (1994-2006). Based on the year of their first registered MS code, the PwMS were categorised into four panels: 2003, 2004, 2005, and 2006. Linkage across registers was conducted by using the unique identity number assigned to all residents in Sweden

Secondly, a reference group was obtained from the same source population as the PwMS. Group-based stratified matching on the joint distribution of sex and age categories was used to form a comparable reference group for each panel in light of the observed differences in the age and sex distribution of the PwMS. Three references were obtained for each MS patient, and drawn without replacement. The reference individuals had no registered MS diagnosis in 1987-2006.

For peer review only - http://bmjopen.bmj.com/site/about/guidelines.xhtml

In total, 2656 PwMS and 7950 reference individuals were included. The year a panel was constituted with newly diagnosed PwMS and reference individuals is, hereafter, referred to as "the year of inclusion" (T_0). The years the four panels were constituted were 2003, 2004, 2005, and 2006, respectively. Observation spanned from ten years prior (T_{-10}) to the year of inclusion (T_0) until five years after inclusion (T_{+5}).

Socio-demographic and income variables were obtained from the Longitudinal Integration Database for Health Insurance and Labour Market Studies (LISA), maintained by Statistics Sweden, for each of the 16 years studied (T_{-10} until T_{+5}) given residence in Sweden at the end of the respective year.

Outcome variable

Levels of earnings before tax were measured over 16 years (T_{-10} to T_{+5}). Student allowances were added to earnings to limit the risk of underestimating younger individuals' income-generating ability in earlier years. In the Swedish sickness absence insurance system, the employer usually pays for day 2-14 of a SA spell.[41] This amount could not be disentangled, and was thus included in our definition of earnings.[42]

All analyses were performed using the annual earnings in Swedish Krona (SEK), inflated to 2005 monetary values by Statistics Sweden's Consumer Price Index.

Explanatory variables

The explanatory variables were: sex (*female, male*), age (categorised in *five-year intervals*), country of birth (*Sweden, outside of Sweden*), educational level (*elementary school*, *high school, university/college*), and type of living area (based on population density: s*maller municipalities, medium-sized municipalities, larger cities*). Further, using a definition set by Statistics Sweden (based on annual income and verifications from workplace), individuals were categorised as being in work during the year in question, work status (*yes*, *no*).[42 43]

For peer review only - http://bmjopen.bmj.com/site/about/guidelines.xhtml

Values for variables pertained to the 31 December of the year of inclusion, except for the type of living area and work status which were obtained annually.

Annual information on type of occupation was also included. Usually, measures of occupation categorise occupations into a hierarchical order, based on the level of specialised skill.[44] Such categorisation is used by Statistics Sweden with ten mutually exclusive groups.[42] However, we also aimed to differentiate between types of occupations by how physically demanding they were, and to what extent the individual was able to have control over his/her working hours (i.e., flexibility at the workplace). Therefore, we collapsed the ten categories into the following five:

1. **Manager**: Individuals classified as managing a group of workers. Managers performed either office work or manual labour. Therefore, this group was analysed separately.
2. **Office work**: Non-physical ("white collar") work.
3. **Manual labour**: Physically demanding ("blue collar") work.
4. **Workplace unknown**: Registered as employed but no information on workplace.
5. **No work**: Not categorised as being in work (i.e., work status: no).

Individuals identified as employed by the armed forces were excluded from the study population (n=18; <0.1%) due to being only a few observations and insufficient information to classify occupation.

Study population

Individuals with missing values for at least one of the socio-demographic variables in the year of inclusion were excluded using list-wise deletion. An exception to this was type of occupation (due to individuals without work). Individuals who had earnings higher than the 99th percentile (of this study) in at least one year were also excluded to control for extreme

For peer review only - http://bmjopen.bmj.com/site/about/guidelines.xhtml

outliers (around 64% of those excluded in this step had earnings above the 99th percentile for more than one year). In total, 112 PwMS and 430 references were excluded from the study, due to employment in armed forces, missing values, or having earnings higher than the 99th percentile in a single year. Accordingly, due to these exclusions, the total number of references does not sum to three times the number of PwMS.

The maximum number of years with information for each of the individuals in the study was 16. Due to migration before/after the year of inclusion or death, not all individuals were in the study population for the entire study period. Nonetheless, 94.4% of PwMS and 93.1% matched references were in the study population for all 16 years. Stratified analyses by country of birth showed that 97.2% of PwMS and 97.5% of the references born in Sweden were included for all 16 years. Corresponding proportions for individuals not born in Sweden were 70.7% and 70.5%, respectively. At the end of follow-up (T_{+5}), 2.0% of all PwMS and 2.1% of the references were no longer in the study population.

Patient and public involvement

Due to the use of unidentifiable data from total population registers, informed consent was not applicable.

Ethical approval

The project was approved by the Regional Ethical Review Board of Stockholm, Sweden.

Insurance system

The universal Swedish sickness absence insurance system involves two main benefits: sickness absence (SA) and disability pension (DP). From the age of 16, people with income from work or unemployment benefits can be granted SA benefits if disease or injury leads to work incapacity. People aged 19-64 can be granted DP if they have long-term or permanently reduced work capacity due to disease or injury. Both benefits can be granted at four levels

For peer review only - http://bmjopen.bmj.com/site/about/guidelines.xhtml

(25, 50, 75, or 100%) of ordinary working hours, but at different replacement rates (SA: approximately 80%, DP: approximately 64% of lost income, up to a ceiling).

Regarding SA, the employer pays the first 14 days of a SA spell, with one uncompensated ("waiting") day at the onset. A physician's certificate is required by day eight. Absence from day 15 onwards is covered by the national Social Insurance Agency, and from day 2 among unemployed.[41]

Statistical methods

The levels and distributions of earnings were analysed for PwMS and references, stratified by time before and after the year of inclusion (T_0). The four panels were pooled in all analyses.

Firstly, annual mean and median earnings were used to describe the central tendencies among PwMS and references. The annual "interquartile range" (IQR); the distance between the 25th and 75th percentile was used to describe potential converging/diverging trends within, and between, the two groups.

Secondly, mean earnings before and after T_0, stratified by educational level and type of occupation at T_0 were calculated. Two-sided t-tests tested the differences in mean earnings between PwMS and the references.

Thirdly, the proportions of individuals on part- or full-time SA and DP during follow-up, stratified by educational level and type of occupation, were examined. For individuals with multiple spells of SA or DP in a single year, the spell with the highest grade (25, 50, 75, or 100%) was selected.

Finally, Tobit regression models were used to control for potential socio-demographic confounding and to estimate differences in levels of earnings between PwMS and references. Earnings are a non-negative outcome and often exhibit a skewed distribution with clustering

For peer review only

For peer review only - http://bmjopen.bmj.com/site/about/guidelines.xhtml

at zero (i.e., distinct differences in distributions between those in paid work compared to those not in paid work). Earnings were thus used as an observable proxy for individuals' ability to support themselves through their own work (i.e., earnings above zero). Tobit models avoid the potential bias of analysing outcomes with "semi-continuous" distributions by ordinary least squares (OLS), which may produce negative predicted values due to assumptions of unlimited linearity in the outcome variable.[45] A Tobit model jointly estimates, using a set of explanatory variables, the probability that the individuals' outcome variable is within a pre-determined bound and the linear relationship between the explanatory variables and the outcome for individuals within the bound.[46] We used the lower bound zero (i.e., no income from work), to observe the actual outcomes, rather than unknown outcomes based on set bound(s), which also means that the observed zeroes were "genuine zeroes".[47]

Separate Tobit regressions were estimated at the time points T_0 and T_{+5} for two different models. The distributions of earnings in these two time periods are shown in Figure 1. The two models used the same set of explanatory variables (MS (*yes/no*), panel membership, sex, age at inclusion, country of birth, and type of living area), with the exception of level of education and type of occupation, which due to high correlation were analysed separately. Model 1a included the level of education at year of inclusion whereas Model 2a included a time-varying variable for the type of occupation. Furthermore, to estimate the potential additional effects of different levels of education or types of occupation on the relationship with levels of earnings, two Tobit models were estimated including two-way interactions between MS and education (Model 1b, university/college as reference) and MS and occupation (Model 2b, Office work as reference), respectively. Statistical tests of whether the estimates deviate enough from zero to reject the assumption of no association were performed at the 95% significance level ($p<0.05$) and presented as beta estimates with 95% confidence intervals (95% CI). Data management and statistical analyses (regression models)

For peer review only - http://bmjopen.bmj.com/site/about/guidelines.xhtml

were conducted in SAS V.9.2, with the exception of the differences in mean earnings calculations, which were calculated in Microsoft Excel.

RESULTS

We identified 2553 PwMS (Panel: 2003: 724; 2004: 648; 2005: 636; and 2006: 545) and included 7584 matched references. The distributions of the socio-demographics for the pooled panels at the year of inclusion (T_0) are presented in Table 1. The majority of the PwMS were women (70%) and aged 30-45 when diagnosed (62%). In the year of inclusion, the majority had at least some high school education, were born in Sweden (89% among the PwMS compared to 84% in the reference group) and lived in larger cities or medium-sized municipalities. Also, in both groups, most individuals had "office work" or "manual labour" as occupations.

For peer review only - http://bmjopen.bmj.com/site/about/guidelines.xhtml

Table 1: Descriptive statistics for the people with multiple sclerosis (PwMS) group and stratified matched references at the year of inclusion (T_0). [a]

		PwMS N=2553[c]	%	References N=7584[c]	%
**Sex** [b]					
	Female	1796	70	5380	71
	Male	757	30	2204	29
**Age** [b]					
	30-34	494	19	1461	19
	35-39	531	21	1576	21
	40-44	574	22	1701	22
	45-49	492	19	1446	19
	50-54	462	18	1400	18
**Educational level**					
	University/college	945	37	2685	35
	High school	1345	53	3801	50
	Elementary school	263	10	1098	14
**Type of occupation**					
	Managers	86	3	259	3
	Office work	1082	42	3020	40
	Manual labour	916	36	2976	39
	Unknown	130	5	352	5
	No work	339	13	977	13
**Type of living area**					
	Larger cities	960	38	2789	37
	Medium-sized municipalities	902	35	2681	35
	Smaller municipalities	691	27	2114	28
**Country of birth**					
	Sweden	2280	89	6356	84
	Outside of Sweden	273	11	1228	16
**Year of inclusion (T_0)**					
	2003	724	28	2150	28
	2004	648	25	1927	25
	2005	636	25	1890	25
	2006	545	21	1617	21

[a] Year of inclusion (T_0) for PwMS: year of first MS diagnosis. Year of inclusion (T_0) for references: year of inclusion to one of the four panels.
[b] Variables used for stratified matching (1_{MS}->3_{REF}).

For peer review only - http://bmjopen.bmj.com/site/about/guidelines.xhtml

Figure 2 shows the development and intra-year distribution of earnings T_{-10} to T_{+5}, for the PwMS and the reference group. The reference group experienced a steady increase in earnings and a predominantly even IQR across the entire period. Whereas, the PwMS' trajectory can be divided into three periods: T_{-10} to T_{-2}; T_{-1} to T_{+1}; and T_{+2} to T_{+5}. The first period (T_{-10} to T_{-2}) was characterised by increasing earnings for PwMS with an even intra-year distribution (measured by IQR). In the second period (T_{-1} to T_{+1}), PwMS' earnings were decreasing and the IQR was diverging. In the third period (T_{+2} to T_{+5}), the level of earnings was stable but IQR diverging.

Table 2 describes mean levels of earnings among PwMS and references, stratified by educational level, and type of occupation at T_0. Differences between subgroups of PwMS were compared to the corresponding reference subgroup at: T_{-5}; T_{-2}; T_{-1}; T_0; and T_{+5}. In total, PwMS had lower earnings at T_{-1}, T_0 and T_{+5} compared to the references. These differences increased with time. In T_0, PwMS' mean earnings were 85% of the references' mean earnings, whereas the corresponding proportion for T_{+5} was 73%.

PwMS with at most elementary or high school education, experienced a decrease in mean earnings from T_{-1} and onwards. For PwMS with university/college education, there was a decrease in mean earnings from T_{-1} to T_0, but an increase from T_0 to T_{+5} (Table 2). The reference group had increasing trends in mean earnings for all levels of education; which resulted in increasing differences in mean earnings between PwMS and the references. At the end of follow-up (T_{+5}) the largest differences in earnings were found among those with at most elementary school education; where the PwMS' mean earnings were SEK 84,000, corresponding to 53% of the mean earnings of their peers in the reference group. Further, PwMS with high school and university/college education also had lower earnings than their peers in the reference group at T_{+5}. The corresponding differences and proportions for high

For peer review only - http://bmjopen.bmj.com/site/about/guidelines.xhtml

school and university/college education were SEK 68,000 (68%) and SEK 63,000 (78%), respectively.

In all time periods, for both PwMS and references, the mean earnings were highest among the managers (Table 2). Further, office workers had higher mean earnings compared to those with manual work. These findings reflected those which we obtained for level of education. PwMS in manual work had lower earnings compared to their reference counterparts at T_{+5} (67%). The corresponding figure for office workers was 78%. However, the largest difference in T_{+5} was observed among managers, with SEK 113,000 less among PwMS than their peers, which corresponded to 70% of the mean earnings of the reference group managers. Finally, those with no work at T_0 had much lower levels of earnings compared to those with known occupations both prior to and after this time point.

For peer review only

328 **Table 2**: Mean earnings [a] of people with multiple sclerosis (PwMS) and differences in mean earnings
329 between the PwMS group and reference group at different lengths in time from the year of inclusion
330 (T_0), stratified by educational level and type of occupation at T_0.

	**T_{-5} PwMS [b] (diff)[c]**	**T_{-2} PwMS [b] (diff)[c]**	**T_{-1} PwMS [b] (diff)[c]**	**T_0 PwMS [b] (diff)[c]**	**T_{+5} PwMS [b] (diff)[c]**
**All**	169 (4)	182 (1)	181 (-6)*	163 (-28)*	166 (-63)*
**Educational level**					
University/college	192 (5)	214 (1)	214 (-6)	203 (-24)*	218 (-63)*
High school	159 (0)	168 (-6)	166 (-12)*	146 (-37)*	145 (-68)*
Elementary school	135 (9)	142 (12)	135 (3)	111 (-25)*	84 (-74)*
**Type of occupation**					
Managers	251 (-12)	308 (-5)	308 (-24)	306 (-27)	258 (-113)*
Office work	207 (2)	230 (-5)	233 (-11)*	219 (-33)*	226 (-65)*
Manual labour	163 (6)	179 (4)	178 (-4)	152 (-36)*	146 (-71)*
Unknown	96 (6)	90 (-5)	99 (-7)	102 (-18)	88 (-69)*
No work	65 (13)*	39 (10)*	20 (6)*	4 (0)	25 (-27)*

331 [a] Earnings: income from work and student allowances, presented in 2005 values as 1000 Swedish Krona (SEK).
332 [b] PwMS (n=2553). References (n=7584).
333 [c] Differences in mean earnings (SEK 1000) between PwMS and references in parentheses. Calculated with two-sided t-tests with unequal
334 variance and unequal numbers of observations.
335 * Differences significantly different from zero ($p<0.05$).
336

For peer review only

338 Table 3 describes the proportion of individuals with at least one incident or prevalent spell of part- or full-time SA or DP in the period T_0 to T_{+5}, stratified by MS, educational level, and type of occupation at T_0. The proportion of individuals with part- and full-time SA or DP was higher among PwMS than in corresponding group among the references at both time points. At T_0, 46.7% of the PwMS had at least one full-time SA spell and 10.0% had part-time SA. Among PwMS, the proportion of part- and full-time SA decreased with time, whereas the proportion of DP increased substantially. At the end of follow-up, 21.0% of the PwMS had at least one part-time DP spell, and the proportion of full-time DP was 25.1%. The patterns were similar for the reference group, but at lower levels at both time points than for the PwMS.

For PwMS, the proportions of part- and full-time SA at T_{+5} were somewhat similar for all levels of education (Table 3). Proportions by type of occupation varied more: managers and individuals with no work had lower proportions of part- and full-time SA than people in the other types of occupations. For PwMS at T_{+5}, the prevalence of individuals with part-time DP was higher than for full-time DP among those with university/college education and with more qualified types of occupations.

For peer review only - http://bmjopen.bmj.com/site/about/guidelines.xhtml

Table 3: Percentage of people with multiple sclerosis (PwMS) who had sickness absence [a] and disability pension [a] in the year of inclusion (T_0) and five years later (T_{+5}), stratified by educational level and type of occupation at T_0.

Year	Factor	Category	Sickness absence PwMS Part-time	Sickness absence PwMS Full-time	Sickness absence Ref [c] Part-time	Sickness absence Ref [c] Full-time	Disability pension PwMS [c] Part-time	Disability pension PwMS [c] Full-Time	Disability pension Ref [c] Part-time	Disability pension Ref [c] Full-time
**T_0**	**Education**	University/college	11.0	43.0	2.6	10.0	3.6	3.8	1.5	2.0
		High school	9.1	49.5	2.1	15.6	5.7	10.0	2.5	6.4
		Elementary school	11.0	45.6	1.3	16.3	8.4	22.8	3.5	16.9
**T_{+5}**		University/college	7.2	17.7	1.6	8.4	21.6	15.4	2.1	2.8
		High school	5.3	17.1	0.8	11.9	20.9	27.3	3.0	8.1
		Elementary school	6.6	13.2	0.8	10.0	19.7	48.3	4.9	19.6
**T_0**	**Occupation**	Managers	16.3	31.4	0.8	5.8	1.2	0.0	1.2	0.4
		Office work	13.3	43.6	2.9	11.0	5.4	1.9	2.0	0.7
		Manual labour	8.5	55.7	2.1	17.2	5.1	3.0	2.7	1.4
		Unknown	10.0	43.9	2.3	17.1	12.3	13.1	2.3	7.7
		No Work [b]	2.1	37.2	0.3	15.0	3.2	49.3	2.4	39.8
**T_{+5}**		Managers	4.7	8.2	0.4	4.7	24.7	15.3	1.2	0.8
		Office work	8.0	18.7	1.5	8.7	25.5	12.7	2.5	1.5
		Manual labour	5.4	19.1	0.8	13.6	20.2	23.7	3.5	2.9
		Unknown	7.9	13.4	1.7	11.3	24.2	33.6	2.9	10.4
		No Work [b]	1.9	8.5	0.8	6.8	6.3	69.7	3.2	45.5
**T_0**	**All**	**-**	**10.0**	**46.7**	**2.1**	**13.7**	**5.2**	**9.1**	**2.3**	**6.3**
**T_{+5}**		**-**	**6.2**	**16.9**	**1.1**	**10.4**	**21.0**	**25.1**	**2.9**	**7.9**

[a] Individuals with spell of sickness absence (SA) or disability pension (DP) with the highest grade (25, 50, 75 or 100%) at T_0 and T_{+5} (i.e., if an individual had both part- and full-time SA in a single year, the individual was categorised as full-time SA. The same procedure was employed for persons with DP).

[b] Individuals with unemployment benefits can be granted SA if the disease/injury is severe enough to prevent them from seeking employment. DP can be granted until the age of 65. In most cases, SA refers to compensation for a spell longer than the first 14 days, which are covered by the employer.

[c] Numbers at year T_0: PwMS (n=2553) and references (n=7584).

Row percent of all individuals (including those without SA or DP) in each sub-group.

For peer review only

For peer review only - http://bmjopen.bmj.com/site/about/guidelines.xhtml

Estimates from the Models 1a and 2a are presented in Table 4. In T_0, the estimates for MS (yes/no) showed similar negative relationships with earnings when comparing the regression that included educational level (T_0: -36.9; 95% CI: -43.0, -30.8) and that which included type of occupation (T_0: -33.3; 95% CI: -38.0, -28.6). The corresponding estimates for T_{+5} were also both negative; but with a larger difference between the estimates when including educational level (T_{+5}: -84.4; 95% CI: -91.5, -77.3) compared to type of occupation (T_{+5}: -49.9; 95% CI: -55.6, -44.2).

A hierarchic pattern in the estimates for educational level and type of occupation was observed. Higher levels of education (i.e., university/college) and more qualified occupations (i.e., managers and office workers) were associated with relatively higher levels of earnings compared to those with lower levels of education (i.e., high school and elementary school) or less qualified occupations.

In the additional Tobit regressions for earnings in the year of inclusion and five years later (Models 1b and 2b), interactions between MS and education or occupation, respectively, were included. In Model 1b, we observed that the interaction between high school education and MS was negative in T_0 (-16.9; 95% CI: -29.6, -4.2). In T_{+5}, the interaction between elementary school education and MS was also negative (-42.7; 95% CI: -68.0, -17.4). Further, interactions between occupation and MS (Model 2b) found a negative association among managers in T_{+5} (-40.7; 95% CI: -68.5, -12.9). Whereas a non-significant negative association was found among manual labour in both T_0 (-5.0; 95% CI: -15.2, 5.2) and T_{+5} (-5.6; 95% CI: -17.8, 6.6).

For peer review only - http://bmjopen.bmj.com/site/about/guidelines.xhtml

Table 4: Cross-sectional Tobit regressions for earnings [a] at the year of inclusion [b] and five years later.

	Education<br>Model 1a		Occupation<br>Model 2a	
	**T_0**<br>Beta estimate<br>(95% CI)	**T_{+5}**<br>Beta estimate<br>(95% CI)	**T_0**<br>Beta estimate<br>(95% CI)	**T_{+5}**<br>Beta estimate<br>(95% CI)
Intercept	217.3 (208.3, 226.3)	285.8 (275.2, 296.4)	241.7 (234.6, 248.8)	279.4 (271.2, 287.6)
MS (ref: No MS) [c]	-36.9 (-43.0, -30.8)	-84.4 (-91.5, -77.3)	-33.3 (-38.0, -28.6)	-49.9 (-55.6, -44.2)
Male (ref: Female) [c]	68.1 (62.4, 73.8)	67.8 (61.1, 74.5)	63.4 (58.9, 67.9)	64.2 (58.9, 69.5)
Age (ref: 30-34 years) [c]				
- 35-39	17.5 (9.3, 25.7)	20.6 (11.0, 30.2)	9.6 (3.1, 16.1)	18.2 (10.8, 25.6)
- 40-44	35.5 (27.5, 43.5)	25.1 (15.7, 34.5)	25.7 (19.2, 32.2)	27.4 (20.0, 34.8)
- 45-49	33.4 (25.0, 41.8)	6.1 (-3.9, 16.1)	24.3 (17.6, 31.0)	16.6 (8.8, 24.4)
- 50-54	30.0 (21.6, 38.4)	-9.4 (-19.4, 0.6)	22.3 (15.6, 29.0)	9.6 (1.6, 17.6)
Country of birth (ref: Sweden) [c]	-78.4 (-86.0, -70.8)	-71.7 (-80.7, -62.7)	-20.3 (-26.6, -14.0)	-19.9 (-27.3, -12.5)
Panel (ref: 2006) [c]				
-2003	-5.1 (-12.5, 2.3)	-9.4 (-18.2, -0.6)	-6.9 (-12.8, -1.0)	-9.3 (-16.2, -2.4)
-2004	-5.7 (-13.3, 1.9)	-2.1 (-11.1, 6.9)	-7.3 (-13.4, -1.2)	-2.5 (-9.6, 4.6)
-2005	-7.8 (-15.4, -0.2)	-6.7 (-15.7, 2.3)	-6.0 (-12.1, 0.1)	-3.1 (-10.2, 4.0)
Type of living area (ref: Larger cities) [d]				
- Medium-sized municipalities	-19.9 (-26.0, -13.8)	-23.3 (-30.6, -16.0)	-20.8 (-25.7, -15.9)	-21.9 (-27.6, -16.2)
- Smaller municipalities	-26.1 (-32.8, -19.4)	-32.7 (-40.5, -24.9)	-29.5 (-34.8, -24.2)	-31.6 (-37.9, -25.3)
Educational level (ref: University/college) [c]				
- High school	-58.1 (-63.8, -52.4)	-79.7 (-86.4, -73.0)	-	-
- Elementary school	-113.2 (-121.8, -104.6)	-148.6 (-158.8, -138.4)	-	-
Type of occupation (ref: Office work) [d]				
- Managers	-	-	63.5 (52.3, 74.7)	87.8 (76.8, 98.8)
- Manual labour	-	-	-65.6 (-70.1, -61.1)	-72.5 (-77.6, -67.4)
- Unknown	-	-	-135.9 (-145.5, -126.3)	-171.6 (-187.3, -155.9)
- No Work	-	-	-384.3 (-394.9, -373.7)	-519.7 (-538.9, -500.5)
Sigma	131.2 (129.6, 132.9)	152.4 (150.4, 154.4)	100.7 (99.4, 102.0)	113.8 (112.3, 115.3)
N	10,137	9,928	10,137	9,928
N censored at zero	1,260	1,505	1,260	1,505
Bayesian information criterion (BIC)	114,727	111,972	107,869	104,307

[a] Earnings: income from work and student allowances. Presented in 2005 values, in SEK 1000.
[b] Year of inclusion (T_0) for people with multiple sclerosis (PwMS): year of first MS diagnosis. Year of inclusion (T_0) for references: year of inclusion to one of four panels. Estimates from Tobit models with lower bound set at zero (i.e., no earnings).
[c] Time-invariant variables: measured at T_0.
[d] Time-variant variables: measured at T_0 or T_{+5}.

For peer review only

For peer review only - http://bmjopen.bmj.com/site/about/guidelines.xhtml

DISCUSSION

In this cohort study we identified 2553 PwMS of working ages and compared them with 7584 references without MS across time points for a period of 16 years. The principal findings were that both groups had similar levels of earnings and annual changes in earnings up until one year before the diagnosis year, thereafter, the growth in earnings levelled off among the PwMS for the remainder of the study period, while earnings for the references continued to increase. These differences remained when we controlled for a number of socio-demographic variables. The largest differences in absolute terms between PwMS and the references were observed for those with elementary school education and for those working as managers. Those with elementary school education also had the largest relative differences. There were also large within-group differences in the development of mean earnings for the PwMS with respect to educational level, and type of occupation. Further, the PwMS had higher rates of part- and full-time DP after the year of diagnosis compared to the references.

This study has several strengths, including identifying the PwMS from nationwide patient registers.[48] Further, the data from the other nationwide registers were also of high quality,[49] which made it possible to conduct our analyses with no loss to follow-up and without self-selection bias. One limitation is that onset of MS always precedes diagnosis, and this time lag varies considerably depending on the experienced symptoms, the individual's healthcare-seeking behaviour, healthcare practices, and timeliness to confirm diagnosis.[2 50] Therefore, the year of diagnosis used in this study can be seen as a biased proxy (although with known direction) for the year of MS onset. Without additional MS-specific data on clinical progression, we could not evaluate the degree to which this time lag affected the findings. The extent to which the time lag correlates with socio-demographic factors is, to the best of our knowledge, not known. Furthermore, educational level and type of occupation were

For peer review only - http://bmjopen.bmj.com/site/about/guidelines.xhtml

highly correlated, and therefore, analysed separately. Hence, when we analysed one of the variables, the effect of the other variable could, to some degree, be incorporated indirectly. The generalisability of the findings may be limited to settings with a similarly functioning welfare system and labour market. Finally, due to data limitations, our definition of earnings includes shorter spells of SA (days 2-14), which may conceal the initial effects of MS, and underestimate the potential effect of MS on earnings for individuals with repeated short SA spells.

Our overall results are in line with previous studies, which have shown non-increasing gross income,[6 15] and high levels of unemployment or SA/DP among PwMS.[3 13 14 32 34 38 51-53] We found that differentiating the analyses by either educational level or type of occupation overall gave similar results. However, the combination of occupation and annual earnings provided additional information, including information on those who were working compared with those not in paid work. Further, comparing the PwMS' earnings with their peers in the occupational reference groups showed that PwMS classified as managers had larger differences in earnings than those in office work. However, it is possible that the large aggregates chosen for categorisation of occupation disguised within-group variation in terms of the physical demands of work tasks. Likewise, the lack of differentiation within the university/college educational category could have concealed strong heterogeneity.

We found considerable heterogeneity within the group of PwMS; while a large proportion appeared to participate on the labour market to a high extent, many had low or no earnings at all. There are many possible explanations of this heterogeneity, with the individuals' educational level and type of occupation as two influential factors within this study. This suggests that the PwMS' work situations are important determinants of subsequent labour market outcomes. We also observed that PwMS with university/college education or with more qualified types of occupations (i.e., office workers or managers) had higher proportions

For peer review only - http://bmjopen.bmj.com/site/about/guidelines.xhtml

of part-time DP (rather than full-time DP) than those with lower education levels or less qualified types of work (i.e, manual workers or those with no work). This could potentially reflect the nature of physical work demands among manual occupations or reflect a lower degree of flexibility to adjust one's work situation than among non-manual occupations, which may lead to higher rates of full-time rather than part-time DP. It was not possible to capture this distinction in the present study. However, it is also possible that our variables, educational level and type of occupation, were proxies for less tangible individual factors. One such factor could be healthcare seeking behaviour, where those with a higher educational level or more qualified occupations may receive the MS diagnosis, and thus treatment, earlier in the disease trajectory. Nevertheless, MS affected an individual's work capacity regardless of whether a diagnosis had been set or not. The new generation of disease modifying treatments to slow disease progression, and thus preserve work capacity, were first introduced in 2006, but not administered to the majority of PwMS until years later.[54] Therefore, it is possible that this could partly explain the observed differences in earnings between the groups. Similarly, the wide distribution of earnings among PwMS could reflect the known heterogeneous progression of the disease - irrespective of educational level or type of occupation.

A possible explanation of the observed increasing differences in earnings between PwMS and individuals without MS, in addition to working fewer hours, and situations of pay for performance, could be due to different opportunities for mobility in the labour market. An individual's wage development is generally associated with possibilities for changing employers. This is especially so in Sweden, with largely centrally negotiated wage increases, with often limited individual performance variation.[55 56] Thus, labour market mobility provides an opportunity for substantial wage improvement, and a MS diagnosis may deter changing employment.[57] More studies of PwMS' or more generally, on chronically ill

For peer review only - http://bmjopen.bmj.com/site/about/guidelines.xhtml

individuals' wages and labour market mobility are needed to increase our knowledge about such mechanisms, e.g., by studying transitions between different occupations, types of employment, and unemployment.[58 59]

Furthermore, the large differences in earnings among individuals with educational levels less than university/college following MS diagnosis compared to the reference group, provides support for the conclusion that the degree of physical labour and/or flexibility at the work place is of importance for remaining in paid work once diagnosed with MS. However, further research is required to explain the observed differences between PwMS and the references in managerial positions.

Lastly, health outcomes research is limited by a lack of measures reflecting the multitude of MS consequences.[60] The traditional MS-specific health outcome measurement tool, Expanded Disability Systems Score (EDSS), typically overemphasises the importance of motor function and poorly reflects cognitive functions and fatigue,[60] two factors strongly influencing work capacity.[18 60-62] Our results suggest that earnings could potentially be a promising outcome measure assessing a composite of functions.[18] The underlying assumption is that the reduction of earnings among PwMS to a great extent reflects MS-related disability. The observed impact of MS on earnings among PwMS across occupational types and educational levels indicates strongly that motor function, which is known to deteriorate slowly, at least in younger patients, is not the sole reason for the decrease in earnings. Thus, indicating the possible influence of fatigue and cognition.[18 61] Furthermore, our findings of earnings development reflect the same trends as the pattern of disability development, as assessed by EDSS, suggesting that earnings may serve as a surrogate outcome measure reflecting the broader consequences of MS.[18] Further studies are, therefore, required with data sets containing both earnings and EDSS scores to compare the two in terms of resolution and validity. Earnings, when available from public registers, such as from tax authorities,

For peer review only - http://bmjopen.bmj.com/site/about/guidelines.xhtml

494 have the additional benefits of minimal data loss and reflecting comprehensive time periods,
495 whereas EDSS outcomes reflect single points in time.[18]

496 CONCLUSIONS

497 From one year prior to the MS diagnosis and onwards, we observed increasingly higher
498 levels of mean earnings in the reference group compared to in the PwMS. Further, we
499 observed within-group heterogeneity among the PwMS; after five years, a large percentage
500 still participated in the labour market, nevertheless, many had low or no earnings at all. We
501 also found that those with at most elementary or high school levels of education or less
502 qualified types of occupations were less alike their respective peers in the reference group
503 than those with university/college levels of education and more qualified types of
504 occupations. Moreover, part- or full-time usage of the sickness insurance was more common
505 among the PwMS – a result that could potentially be used to describe the observed
506 heterogeneity of earnings among the PwMS.

For peer review only - http://bmjopen.bmj.com/site/about/guidelines.xhtml

List of abbreviations used

CI: Confidence intervals.

DP: Disability pension.

EDSS: The expanded disability systems score.

IQR: Interquartile range.

MS: Multiple sclerosis.

OLS: Ordinary least squares.

PwMS: People with multiple sclerosis.

SA: Sickness absence.

SEK: Swedish Krona.

For peer review only

For peer review only - http://bmjopen.bmj.com/site/about/guidelines.xhtml

Declarations

Ethics approval

The project was approved by the Regional Ethical Review Board, Stockholm, Sweden.

Acknowledgements

Not applicable

Authors' contributions

MW, PT, KA, EP, MS, and EF participated in the design of the study. MW performed the data management and performed all analyses. All authors (MW, CM, PT, KA, EP, JH, MS, and EF) contributed to the interpretations of the results. MW and CM drafted the manuscript, PT, KA, EP, JH, MS, and EF contributed by revising for important intellectual content. All authors have approved the final version of the manuscript.

Competing interests statement

MW, CM, PT and EF were partly funded by Biogen. ED and MS have no competing interests to declare. KA has received unrestricted grants from Biogen. JH received honoraria for serving on advisory boards for Biogen and Novartis and speaker's fees from Biogen, Merck-Serono, Bayer-Schering, Teva and Sanofi-Aventis. He has served as P.I. for projects sponsored by, or received unrestricted research support from, Biogen, Merck-Serono, TEVA, Novartis, and Bayer-Schering. His MS research is funded by the Swedish Research Council.

Funding

The project was funded by unrestricted grants from Biogen and the Swedish Research Council for Health, Working Life and Welfare. The design of the study, data collection, analyses, interpretations of data, and writing of manuscript was performed without involvement of the funding bodies. Biogen was given the opportunity to comment on the manuscript before submission.

Data statement

Not applicable. The data cannot be made publically available due to privacy regulations. According to the General Data Protection Regulation, the Swedish, the Swedish Data Protection Act, the Swedish Ethical Review Act, and the Public Access to Information and Secrecy Act, data can only be made available for specific purposes, including research that meets the criteria for access to this type of sensitive and confidential data as determined by a legal review. Readers may contact Professor Kristina Alexanderson (kristina.alexanderson@ki.se) regarding the data.

For peer review only

LEGENDS

Figure One

Figure 1: Distribution of earnings in the total study population, at year of inclusion (n=10,137) and five years later (n=9928), in 1000 Swedish Krona (SEK).

Earnings: Combined income from work and student allowance, calculated in 2005 monetary values. The years of inclusion (T_0) vary between 2003-2006. They are for PwMS the year of the first MS diagnosis and for references the year of inclusion into one of the four panels.

Figure Two

Figure 2: Distribution (box and whisker plots) of earnings by time, for PwMS (n=2553) and matched references (n=7584), in 1000 Swedish Krona (SEK).

Earnings: Combined income from work and student allowance, calculated in 2005 monetary values. The years of inclusion (T_0) vary between 2003-2006. They are for PwMS the year of the first MS diagnosis and for references the year of inclusion into one of the four panels. Observations that were larger than 1.5*IQR (Interquartile Range) are circled. Symbols in the box plot: bar = median earnings, diamond = mean earnings. Line across box-plots = median earnings for the reference group at T_0 (SEK 204,850).

For peer review only

References

1. Tullman MJ. A review of current and emerging therapeutic strategies in multiple sclerosis. *Am J Manag Care* 2013;19(2 Suppl):S21-7.
2. Brundin L, Kobelt G, Berg J, et al. New insights into the burden and costs of multiple sclerosis in Europe: Results for Sweden. *Mult Scler* 2017;23:179-91. doi: 10.1177/1352458517708682
3. Pearson J, Alla S, Clarke S, et al. Multiple Sclerosis impact on employment and income in New Zealand. *Acta Neurol Scand* 2017;136(3):223–32. doi: 10.1111/ane.12714
4. Krause I, Kern S, Horntrich A, et al. Employment status in multiple sclerosis: impact of disease-specific and non-disease-specific factors. *Mult Scler* 2013;19(13):1792–99. doi: 10.1177/1352458513485655
5. Naci H, Fleurence R, Birt J, et al. Economic burden of multiple sclerosis: a systematic review of the literature. *Pharmacoeconomics* 2010;28(5):363-79. doi: 10.2165/11532230-000000000-00000
6. Pfleger CCH, Flachs EM, Koch-Henriksen N. Social consequences of multiple sclerosis (1): early pension and temporary unemployment—a historical prospective cohort study. *Mult Scler* 2010;16(1):121-26.
7. Pugliatti M, Rosati G, Carton H, et al. The epidemiology of multiple sclerosis in Europe. *Eur J Neurol* 2006;13(7):700-22.
8. Chruzander C, Tinghög P, Ytterberg C, et al. Longitudinal changes in sickness absence and disability pension, and associations between disability pension and disease-specific and contextual factors and functioning, in people with multiple sclerosis. *J Neurol Sci* 2016
9. Ernstsson O, Gyllensten H, Alexanderson K, et al. Cost of Illness of Multiple Sclerosis-A Systematic Review. *PLoS One* 2016;11(7):e0159129.
10. Gyllensten H, Wiberg M, Alexanderson K, et al. Costs of illness of multiple sclerosis in Sweden: a population-based register study of people of working age. *Eur J Health Econ* 2018;19(3):435-46. doi: 10.1007/s10198-017-0894-6 [published Online First: 2017 May 9]
11. Gyllensten H, Wiberg M, Alexanderson K, et al. Comparing costs of illness of multiple sclerosis in three different years: A population-based study. *Mult Scler* 2018;24(4):520-28. doi: 10.1177/1352458517702549 [published Online First: 2017 Apr 3]
12. Green G, Todd J, Pevalin D. Biographical disruption associated with multiple sclerosis: using propensity scoring to assess the impact. *Soc Sci Med* 2007;65(3):524-35. doi: 10.1016/j.socscimed.2007.03.007
13. Wiberg M, Friberg E, Stenbeck M, et al. Sources and level of income among individuals with multiple sclerosis compared to the general population: A nationwide population-based study. *Multiple Sclerosis Journal* 2015;21(13):1730-41. doi: 10.1177/1352458515570767
14. Jennum P, Wanscher B, Frederiksen J, et al. The socioeconomic consequences of multiple sclerosis: a controlled national study. *Eur Neuropsychopharmacol* 2012;22(1):36-43. doi: 10.1016/j.euroneuro.2011.05.001
15. Landfeldt E, Castelo-Branco A, Svedbom A, et al. Personal Income Before and After Diagnosis of Multiple Sclerosis. *Value Health* 2018;21(5):590-95. doi: https://doi.org/10.1016/j.jval.2017.09.021 [published Online First: 2017 Nov 15]
16. Murley C, Mogard O, Wiberg M, et al. Trajectories of disposable income among people of working ages diagnosed with multiple sclerosis: a nationwide register-based cohort study in Sweden 7 years before to 4 years after diagnosis with a population-based reference group. *BMJ Open* 2018;8(5):e020392. doi: 10.1136/bmjopen-2017-020392 [published Online First: 2018/05/11]
17. Thormann A, Sørensen P, Koch-Henriksen N, et al. Chronic comorbidity in multiple sclerosis is associated with lower incomes and dissolved intimate relationships. *Eur J Neurol* 2017;24(6):825-34. doi: 10.1111/ene.13297

For peer review only - http://bmjopen.bmj.com/site/about/guidelines.xhtml

18. Kavaliunas A, Wiberg M, Tinghog P, et al. Earnings and Financial Compensation from Social Security Systems Correlate Strongly with Disability for Multiple Sclerosis Patients. *PLoS One* 2015;10(12):e0145435. doi: ARTN e014543510.1371/journal.pone.0145435
19. Abegunde DO, Stanciole AE. The economic impact of chronic diseases: how do households respond to shocks? Evidence from Russia. *Soc Sci Med* 2008;66(11):2296-307.
20. Carlsson KS, Landin-Olsson M, Nyström L, et al. Long-term detrimental consequences of the onset of type 1 diabetes on annual earnings—evidence from annual registry data in 1990–2005. *Diabetologia* 2010;53(6):1084-92.
21. Løkke A, Hilberg O, Kjellberg J, et al. Economic and Health Consequences of COPD Patients and Their Spouses in Denmark-1998-2010. *COPD* 2014;11(3):237-46.
22. Mitchell JM, Burkhauser RV, Pincus T. The importance of age, education, and comorbidity in the substantial earnings losses of individuals with symmetric polyarthritis. *Arthritis Rheum* 1988;31(3):348-57.
23. Shanahan EM, Smith MD, Roberts-Thomson L, et al. The effect of rheumatoid arthritis on personal income in Australia. *Intern Med J* 2008;38(7):575-79.
24. Ward MM, Javitz HS, Smith WM, et al. Lost income and work limitations in persons with chronic respiratory disorders. *J Clin Epidemiol* 2002;55(3):260-68.
25. Wolfe F, Michaud K, Choi HK, et al. Household income and earnings losses among 6,396 persons with rheumatoid arthritis. *J Rheumatol* 2005;32(10):1875-83.
26. Confavreux C, Vukusic S, Adeleine P. Early clinical predictors and progression of irreversible disability in multiple sclerosis: an amnesic process. *Brain* 2003;126(Pt 4):770-82.
27. Ruggieri R, Palermo R, Vitello G, et al. Cognitive impairment in patients suffering from relapsing-remitting multiple sclerosis with EDSS≤ 3.5. *Acta Neurol Scand* 2003;108(5):323-26.
28. Degenhardt A, Ramagopalan SV, Scalfari A, et al. Clinical prognostic factors in multiple sclerosis: a natural history review. *Nat Rev Neurol* 2009;5(12):672-82.
29. Langer-Gould A, Popat RA, Huang SM, et al. Clinical and demographic predictors of long-term disability in patients with relapsing-remitting multiple sclerosis: a systematic review. *Arch Neurol* 2006;63(12):1686-91.
30. Confavreux C, Vukusic S. Age at disability milestones in multiple sclerosis. *Brain* 2006;129(Pt 3):595-605. doi: 10.1093/brain/awh714
31. Beatty WW, Blanco CR, Wilbanks SL, et al. Demographic, clinical, and cognitive characteristics of multiple sclerosis patients who continue to work. *Neurorehabil Neural Repair* 1995;9(3):167-73.
32. Busche K, Fisk J, Murray T, et al. Short Term Predictors of Unemployment in Multiple Sclerosis Patients. *Can J Neurol Sci* 2003;30(2):137-42. doi: doi.org/10.1017/S0317167100053403
33. Grønning M, Hannisdal E, Mellgren SI. Multivariate analyses of factors associated with unemployment in people with multiple sclerosis. *J Neurol Neurosurg Psychiatry* 1990;53(5):388-90.
34. Lunde HMB, Telstad W, Grytten N, et al. Employment among Patients with Multiple Sclerosis-A Population Study. *PLoS One* 2014;9(7):e103317.
35. Patti F, Pozzilli C, Montanari E, et al. Effects of education level and employment status on HRQoL in early relapsing-remitting multiple sclerosis. *Mult Scler* 2007;13(6):783-91.
36. Roessler RT, Rumrill PD, Fitzgerald SM. Predictors of employment status for people with multiple sclerosis. *Rehabil Couns Bull* 2004;47(2):96-103.
37. Smith MM, Arnett PA. Factors related to employment status changes in individuals with multiple sclerosis. *Mult Scler* 2005;11(5):602-09.
38. Glad SB, Nyland H, Aarseth JH, et al. How long can you keep working with benign multiple sclerosis? *J Neurol Neurosurg Psychiatry* 2011;82(1):78-82. doi: 10.1136/jnnp.2010.210732 [published Online First: 2010/08/31]
39. Moore P, Harding KE, Clarkson H, et al. Demographic and clinical factors associated with changes in employment in multiple sclerosis. *Mult Scler* 2013:1352458513481396.

For peer review only - http://bmjopen.bmj.com/site/about/guidelines.xhtml

40. Findling O, Baltisberger M, Jung S, et al. Variables Related to Working Capability among Swiss Patients with Multiple Sclerosis—A Cohort Study. *PLoS One* 2015
41. Social Insurance in Figures 2014. Stockholm, Sweden, 2014.
42. Background Facts, Labour and Education Statistics 2011:4, Integrated database for labour market research. Stockholm: Statistics Sweden, 2011.
43. Rikets indelningar: årsbok över regionala indelningar med koder, postadresser, telefonnummer [Country classifications: yearbook of regional classifications with codes, postal addresses, phone numbers 2003]. Stockholm: Statistics Sweden, 2003.
44. SSYK 96: Standard för svensk yrkesklassificering 1996 [SSYK 96: Standard for Swedish occupation classification 1996]. Swedish Standard Classification of Occupations. Örebro: Statistics Sweden, 1998.
45. Wooldridge JM. Corner solution outocmes and censored regression models. Econometric analysis of cross section and panel data. Cambridge, Massachusetts: MIT Press 2002.
46. Tobin J. Estimation of relationships for limited dependent variables. *Econometrica* 1958;26(1):24-36.
47. Humphreys BR. Dealing with zeros in economic data. University of Alberta, Alberta: Department of Economics, 2013.
48. Ludvigsson JF, Andersson E, Ekbom A, et al. External review and validation of the Swedish national inpatient register. *BMC Public Health* 2011;11(1):450.
49. Ludvigsson JF, Almqvist C, Bonamy A-KE, et al. Registers of the Swedish total population and their use in medical research. *Eur J Epidemiol* 2016;31(2):125-36.
50. Thompson AJ, Banwell BL, Barkhof F, et al. Diagnosis of multiple sclerosis: 2017 revisions of the McDonald criteria. *Lancet Neurol* 2018;17(2):162-73. doi: 10.1016/S1474-4422(17)30470-2
51. Glad S, Aarseth J, Nyland H, et al. Benign multiple sclerosis: a need for a consensus. *Acta Neurol Scand* 2010;122(s190):44-50.
52. Julian LJ, Vella L, Vollmer T, et al. Employment in multiple sclerosis. *J Neurol* 2008;255(9):1354-60.
53. Simmons RD, Tribe KL, McDonald EA. Living with multiple sclerosis: longitudinal changes in employment and the importance of symptom management. *J Neurol* 2010;257(6):926-36.
54. Eriksson I, Komen J, Piehl F, et al. The changing multiple sclerosis treatment landscape: impact of new drugs and treatment recommendations. *Eur J Clin Pharmacol* 2018;74(5):663-70. doi: 10.1007/s00228-018-2429-1 [published Online First: 2018/02/13]
55. Nyberg A, Magnusson Hanson LL, Leineweber C, et al. Do Predictors of Career Success Differ between Swedish Women and Men? Data from the Swedish Longitudinal Occupational Survey of Health (SLOSH). *PLoS One* 2015;10(10):e0140516. doi: 10.1371/journal.pone.0140516 [published Online First: 2015/10/27]
56. Muffels R, Luijkx R. Labour market mobility and employment security of male employees in Europe:trade-off'orflexicurity'? *Work, employment and Society* 2008;22(2):221-42.
57. McAllister A, Nylen L, Backhans M, et al. Do 'flexicurity' Policies Work for People With Low Education and Health Problems? A Comparison of Labour Market Policies and Employment Rates in Denmark, The Netherlands, Sweden, and the United Kingdom 1990-2010. *Int J Health Serv* 2015;45(4):679-705. doi: 10.1177/0020731415600408 [published Online First: 2015/08/15]
58. Ekberg K, Wahlin C, Persson J, et al. Is mobility in the labor market a solution to sustainable return to work for some sick listed persons? *Journal of occupational rehabilitation* 2011;21(3):355-65. doi: 10.1007/s10926-011-9322-4 [published Online First: 2011/07/26]
59. Nordstrom K, Ekberg K, Hemmingsson T, et al. Sick leave and the impact of job-to-job mobility on the likelihood of remaining on the labour market--a longitudinal Swedish register study. *BMC Public Health* 2014;14:305. doi: 10.1186/1471-2458-14-305 [published Online First: 2014/04/04]

For peer review only - http://bmjopen.bmj.com/site/about/guidelines.xhtml

60. van Munster CEP, Uitdehaag BMJ. Outcome Measures in Clinical Trials for Multiple Sclerosis. *CNS drugs* 2017;31(3):217-36. doi: 10.1007/s40263-017-0412-5

61. Kavaliunas A, Karrenbauer VD, Tinghog P, et al. Cognitive function predicts work disability among multiple sclerosis patients. *Mult Scler* 2017;23:479-80.

62. Kavaliunas A, Karrenbauer VD, Gyllensten H, et al. Cognitive function is a major determinant of income among multiple sclerosis patients in Sweden acting independently from physical disability. *Mult Scler* 2017:1352458517740212. doi: 10.1177/1352458517740212

For peer review only

For peer review only - http://bmjopen.bmj.com/site/about/guidelines.xhtml

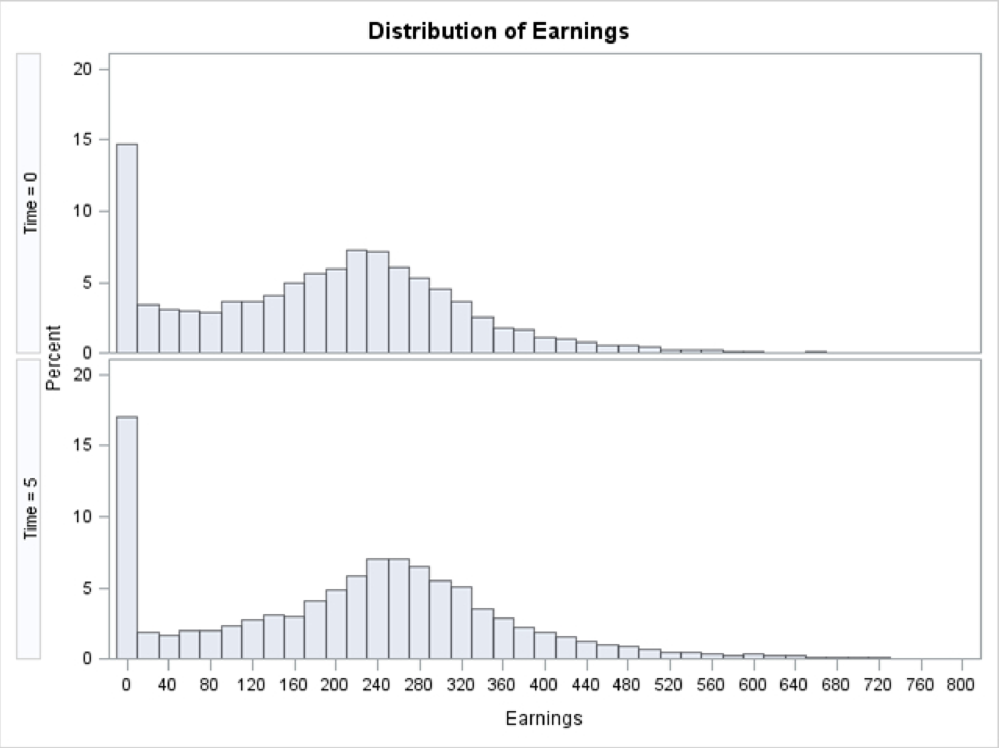

Distribution of earnings in the total study population, at year of inclusion (n=10,137) and five years later (n=9928), in 1000 Swedish Krona (SEK).Earnings: Combined income from work and student allowance, calculated in 2005 monetary values. The years of inclusion (T0) vary between 2003-2006. They are for PwMS the year of the first MS diagnosis and for references the year of inclusion into one of the four panels.

54x40mm (300 x 300 DPI)

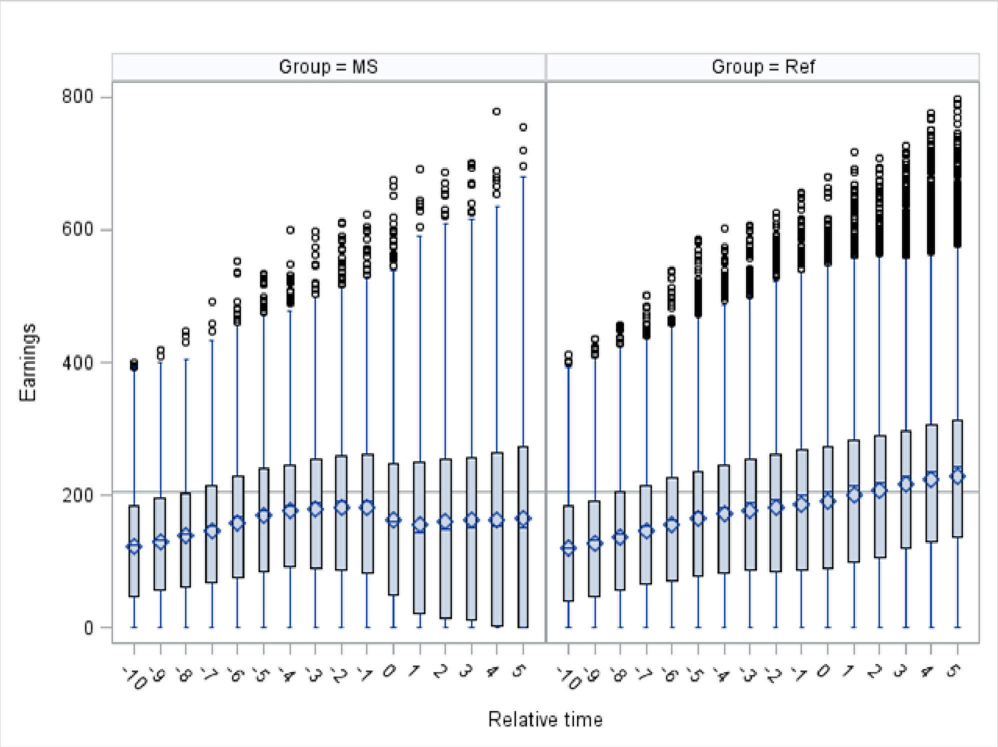

Distribution (box and whisker plots) of earnings by time, for PwMS (n=2553) and matched references (n=7584), in 1000 Swedish Krona (SEK). Earnings: Combined income from work and student allowance, calculated in 2005 monetary values. The years of inclusion (T0) vary between 2003-2006. They are for PwMS the year of the first MS diagnosis and for references the year of inclusion into one of the four panels. Observations that were larger than 1.5*IQR (Interquartile Range) are circled. Symbols in the box plot: bar = median earnings, diamond = mean earnings. Line across box-plots = median earnings for the reference group at T0 (SEK 204,850).

54x40mm (300 x 300 DPI)

STROBE 2007 (v4) checklist of items to be included in reports of observational studies in epidemiology*
Checklist for cohort, case-control, and cross-sectional studies (combined)

Section/Topic	Item #	Recommendation	Reported on page #
**Title and abstract**	1	(*a*) Indicate the study's design with a commonly used term in the title or the abstract	1 (title)<br>2 (abstract)
		(*b*) Provide in the abstract an informative and balanced summary of what was done and what was found	2-3
**Introduction**			
Background/rationale	2	Explain the scientific background and rationale for the investigation being reported	5-6
Objectives	3	State specific objectives, including any pre-specified hypotheses	6-7 (in the form of study aims)
**Methods**			
Study design	4	Present key elements of study design early in the paper	7 first paragraph of Methods
Setting	5	Describe the setting, locations, and relevant dates, including periods of recruitment, exposure, follow-up, and data collection	7-8
Participants	6	(*a*) *Cohort study*—Give the eligibility criteria, and the sources and methods of selection of participants. Describe methods of follow-up<br>*Case-control study*—Give the eligibility criteria, and the sources and methods of case ascertainment and control selection. Give the rationale for the choice of cases and controls<br>*Cross-sectional study*—Give the eligibility criteria, and the sources and methods of selection of participants	Cohort study<br>7-10.
		(*b*) *Cohort study*—For matched studies, give matching criteria and number of exposed and unexposed<br>*Case-control study*—For matched studies, give matching criteria and the number of controls per case	
Variables	7	Clearly define all outcomes, exposures, predictors, potential confounders, and effect modifiers. Give diagnostic criteria, if applicable	8- outcome variable<br>8-9 explanatory variables
Data sources/ measurement	8*	For each variable of interest, give sources of data and details of methods of assessment (measurement). Describe comparability of assessment methods if there is more than one group	8- source<br>8-9 measurement and classification of variables
Bias	9	Describe any efforts to address potential sources of bias	12
Study size	10	Explain how the study size was arrived at	7-8

For peer review only

Quantitative variables	11	Explain how quantitative variables were handled in the analyses. If applicable, describe which groupings were chosen and why	11-12
Statistical methods	12	(*a*) Describe all statistical methods, including those used to control for confounding	11-12
		(*b*) Describe any methods used to examine subgroups and interactions	12 – by education and occupation
		(*c*) Explain how missing data were addressed	10
		(*d*) *Cohort study*—If applicable, explain how loss to follow-up was addressed<br>*Case-control study*—If applicable, explain how matching of cases and controls was addressed<br>*Cross-sectional study*—If applicable, describe analytical methods taking account of sampling strategy	10
		(*e*) Describe any sensitivity analyses	NA
**Results**			
Participants	13*	(a) Report numbers of individuals at each stage of study—eg numbers potentially eligible, examined for eligibility, confirmed eligible, included in the study, completing follow-up, and analysed	10
		(b) Give reasons for non-participation at each stage	10
		(c) Consider use of a flow diagram	NA
Descriptive data	14*	(a) Give characteristics of study participants (eg demographic, clinical, social) and information on exposures and potential confounders	13 and Table 1
		(b) Indicate number of participants with missing data for each variable of interest	10/ NA
		(c) *Cohort study*—Summarise follow-up time (eg, average and total amount)	-
Outcome data	15*	*Cohort study*—Report numbers of outcome events or summary measures over time	15-19
		Case-control study—Report numbers in each exposure category, or summary measures of exposure	
		Cross-sectional study—Report numbers of outcome events or summary measures	
Main results	16	(*a*) Give unadjusted estimates and, if applicable, confounder-adjusted estimates and their precision (eg, 95% confidence interval). Make clear which confounders were adjusted for and why they were included	20-21
		(*b*) Report category boundaries when continuous variables were categorized	NA
		(*c*) If relevant, consider translating estimates of relative risk into absolute risk for a meaningful time period	
Other analyses	17	Report other analyses done—eg analyses of subgroups and interactions, and sensitivity analyses	
**Discussion**			
Key results	18	Summarise key results with reference to study objectives	22
Limitations	19	Discuss limitations of the study, taking into account sources of potential bias or imprecision. Discuss both direction and magnitude of any potential bias	22-23
Interpretation	20	Give a cautious overall interpretation of results considering objectives, limitations, multiplicity of analyses, results from similar studies, and other relevant evidence	23-25

For peer review only

Generalisability	21	Discuss the generalisability (external validity) of the study results	23
**Other information**			
Funding	22	Give the source of funding and the role of the funders for the present study and, if applicable, for the original study on which the present article is based	28

*Give information separately for cases and controls in case-control studies and, if applicable, for exposed and unexposed groups in cohort and cross-sectional studies.
Note: An Explanation and Elaboration article discusses each checklist item and gives methodological background and published examples of transparent reporting. The STROBE checklist is best used in conjunction with this article (freely available on the Web sites of PLoS Medicine at http://www.plosmedicine.org/, Annals of Internal Medicine at http://www.annals.org/, and Epidemiology at http://www.epidem.com/). Information on the STROBE Initiative is available at www.strobe-statement.org.

For peer review only

BMJ Open

Earnings among people with multiple sclerosis compared to references, in total and by educational level and type of occupation: a population-based cohort study at different points in time

Journal:	*BMJ Open*
Manuscript ID	bmjopen-2018-024836.R2
Article Type:	Research
Date Submitted by the Author:	29-Mar-2019
Complete List of Authors:	Wiberg, Michael; Karolinska Institutet Department of Clinical Neuroscience, Division of Insurance Medicine; Swedish Social Insurance Agency, Department of Analysis and Forecast<br>Murley, Chantelle; Karolinska Institutet Department of Clinical Neuroscience, Division of Insurance Medicine<br>Tinghög, Petter; Karolinska Institutet Department of Clinical Neuroscience, Division of Insurance Medicine; Red Cross University College<br>Alexanderson, Kristina; Karolinska Institutet Department of Clinical Neuroscience, Division of Insurance Medicine<br>Palmer, Edward; Uppsala University , Department of Economics, Uppsala Center for Labor Studies<br>Hillert, Jan; Karolinska Institutet Department of Clinical Neuroscience, Division of Neurology<br>Stenbeck, Magnus; Karolinska Institutet Department of Clinical Neuroscience, Division of Insurance Medicine<br>Friberg, Emilie; Karolinska Institutet Department of Clinical Neuroscience, Division of Insurance Medicine
**Primary Subject Heading**:	Neurology
Secondary Subject Heading:	Epidemiology
Keywords:	Multiple sclerosis < NEUROLOGY, Earnings, Sick leave, Disability pension, Work, Social Insurance

SCHOLARONE™
Manuscripts

Earnings among people with multiple sclerosis compared to references, in total and by educational level and type of occupation: a population-based cohort study at different points in time.

Michael Wiberg[1,2], Chantelle Murley[1], Petter Tinghög[1,3], Kristina Alexanderson[1], Edward Palmer[4], Jan Hillert[5], Magnus Stenbeck[1] and Emilie Friberg[1].

[1] Department of Clinical Neuroscience, Division of Insurance Medicine, Karolinska Institutet, SE 171 77 Stockholm, Sweden.

[2] Department of Analysis and Forecast, Swedish Social Insurance Agency, 103 51 Stockholm, Sweden.

[3] Department of Health Sciences, Swedish Red Cross University College, 141 21 Huddinge, Sweden.

[4] Uppsala Department of Economics and Center for Labor Studies, 753 13 Uppsala, Sweden.

[5] Department of Clinical Neuroscience, Division of Neurology, Karolinska Institutet, SE 171 77 Stockholm, Sweden.

Corresponding author: Chantelle Murley

Phone +46(0)8524 832 24

Fax +46(0)8524 832 05

wiberg.michael@gmail.com, chantelle.murley@ki.se, Petter.Tinghog@rkh.se, Kristina.Alexanderson@ki.se, Edward.E.Palmer@gmail.com, Jan.Hillert@ki.se, Magnus.Stenbeck@ki.se, Emilie.Friberg@ki.se.

Keywords (6): MS, Earnings, Sick leave, Disability pension, Work, Social insurance.

Word counts:

Abstract: 300 (max 300)

Main text excluding title page, abstract, references, figures and tables: 4972

Number of references: 64.

Number of tables: 4.

Number of figures: 2.

Number of supplements: 1

For peer review only

ABSTRACT

Objectives

To investigate earnings among people with multiple sclerosis (MS) before and after MS diagnosis compared to people without MS (PwMS), and if identified differences were associated with educational levels and types of occupations. Furthermore, to assess the proportions on sickness absence (SA) and disability pension (DP) in both groups.

Design

Population-based longitudinal cohort study, 10 years before until 5 years after MS diagnosis.

Setting

Working-age population using microdata linked from nationwide Swedish registers.

Participants

Residents in Sweden in 2004 aged 30-54 with MS diagnosed in 2003-2006 (n=2553), and references without MS (n=7584) randomly selected by stratified matching.

Outcome measures

Quartiles of earnings were calculated for each study year prior to and following the MS diagnosis. Mean earnings, by educational level and type of occupation, before and after diagnosis were compared using t-tests. Tobit regressions investigated the associations of earnings with individual characteristics. The proportions on SA and/or DP, by educational level and type of occupation, for the diagnosis year and 5 years later were compared.

For peer review only

Results

Differences in earnings between PwMS and references were observed beginning one year before diagnosis, and increased thereafter. PwMS had lower mean earnings for the diagnosis year (difference= SEK 28,000, $p < 0.05$), and five years after diagnosis this difference had more than doubled ($p < 0.05$). These differences remained after including educational level and type of occupation. Overall, the earnings of PwMS with university education and/or more qualified occupations were most like their reference peers. The proportions on SA and DP were higher among PwMS than the references.

Conclusions

The results suggest that the PwMS' earnings are lower than the references' beginning shortly before MS diagnosis, with this gap increasing thereafter. Besides SA and DP, the results indicate that educational level and type of occupation are influential determinants of the large heterogeneity of PwMS' earnings.

For peer review only

For peer review only - http://bmjopen.bmj.com/site/about/guidelines.xhtml

ARTICLE SUMMARY

Strengths and limitations:

- The main strengths of this study include the population-based longitudinal cohort design, use of high-quality nationwide registers and a large cohort of people with newly registered multiple sclerosis (MS) diagnoses, which enabled both analyses of earnings on a nationwide scale covering 16 years per individual with MS and comparisons with a matched reference group.
- Potential selection bias was limited by using individual-level information about all resident in Sweden encompassing both the people with MS and the reference group, which is seldom the case for these types of studies.
- Extensive annual data on socio-demographics, occupation, and sickness absence and disability pension could be included for ten consecutive years before the year of MS diagnosis and five years after, to further understand the situation before and after MS diagnosis.
- The measure of earnings included the sick pay for employees with short-term sickness absence spells (≤14 days) which might have underestimated the effect of MS on earnings if people with MS have a higher frequency of such spells than the reference group, as lost earnings due to sickness absence are not fully compensated in Sweden.
- Another potential weakness was that we only had information on the MS diagnosis date rather than MS onset date, however, it is unknown whether there are systematic differences among the people with MS regarding the time between onset and diagnosis.

For peer review only - http://bmjopen.bmj.com/site/about/guidelines.xhtml

BACKGROUND

Multiple sclerosis (MS) is a chronic, often progressive, disease that usually onsets in working ages.[1] The combination of the relatively early onset age and chronic nature may affect the individual's economic situation for a substantial part of their working life, through reduced productivity (and wage) per unit of time worked and/or the number of hours worked.[2-6] Furthermore, MS poses a high cost to society in terms of both direct and indirect costs (from productivity losses).[2 5 7-11] Previous studies have focused predominantly on societal cost burdens, whereas the effects of MS on the development of the individuals' earnings, including possible differences by educational level and type of occupation, have not been extensively researched.

Several studies have found that people with MS (hereafter "PwMS") are to a greater extent not in paid work compared to people without MS.[3 4 6 12-14] However, sorting individuals into conventional labour market categories (e.g., employed/unemployed) obscures more nuanced information about the gradual effect of the disease on the individuals' labour market attachment.

An alternative measure for labour market attachment is the development of individuals' earnings. There are a growing number of studies (including MS-specific[3 6 14-18]) showing negative associations between chronic diseases and individuals' earnings.[3 6 13-25]

The progression of MS is heterogeneous[26 27] and accordingly difficult to predict at the individual level using demographic factors,[28] beyond using age at onset.[29 30] Younger age at onset and educational level have also been shown to influence labour market outcomes among PwMS.[4 31-38] Furthermore, educational level may also suggest the degree of physical labour and/or flexibility at the workplace.[34 36-38]

For peer review only - http://bmjopen.bmj.com/site/about/guidelines.xhtml

In working-aged individuals, educational level and type of occupation are highly correlated (e.g., university education increases possibilities for more qualified occupations). However, this does not necessarily hold for individuals with chronic diseases, as the disease might hinder them from performing the work corresponding to their educational level. Furthermore, one's educational level may remain unchanged above a certain age whereas an individual's occupation can vary over time, both due to individual and macro-economic factors. For this reason, in order to analyse the effects of a progressively disabling disease on work, the actual work performed (i.e., earnings), may be more informative than the individual's educational level.

Furthermore, PwMS to a high extent work part-time.[2 37 39 40] This is especially relevant for Sweden, where compensation for sickness absence (SA) and disability pension (DP) can be for either part- or full-time absences from ordinary working hours. Increasing utilisation of these compensation systems (which do not fully compensate the lost earnings) would over time result in increasing differences in annual earnings between PwMS and other wage earners, with all other factors equal.[15] Studies of such associations comparing different time points are still very few and the possible differences by the individuals' educational level and the type of work remain largely unexplored. The present study aims at improving our knowledge in this respect.

The aims of this study were three-fold:

1. To examine levels and distributions of earnings of PwMS compared to population-based matched references, during the years prior to and after the year of diagnosis with MS;
2. To explore whether the level of education and the type of occupation correlate with the within group differences in earnings among PwMS; and

For peer review only - http://bmjopen.bmj.com/site/about/guidelines.xhtml

3. To compare the proportions of PwMS and matched references covered by part- or full-time SA and DP benefits.

METHODS

A population-based longitudinal cohort study was conducted using information from Statistics Sweden on all individuals living in Sweden on 31 December 2004. The study population was attained through two steps.

Firstly, we identified all individuals aged 30-54 years, who in 2003-2006 had their first registered MS diagnosis (ICD-9: 340, ICD-10: G35). Information on MS diagnoses was obtained from the following nationwide registers: inpatient hospital healthcare (data from 1987-2006), specialised outpatient healthcare (2001-2006), and outpatient surgery (1997-2000) from the National Patient Register maintained by the National Board of Health and Welfare; and information on SA (2005-2006) and DP (1994-2006) compensated by the Swedish Social Insurance Agency from their Micro-Data for Analysis of the Social Insurance System (MiDAS). Based on the year of their first registered MS code, the PwMS were categorised into four panels: 2003, 2004, 2005, and 2006. Linkages across the registers were conducted by using the unique personal identity number assigned to all residents in Sweden.

Secondly, a reference group was obtained from the same source population as the PwMS. Group-based stratified matching on the joint distribution of sex and age categories was used to form a comparable reference group for each panel in light of the observed differences in the distribution of PwMS by age and sex. Three references were obtained for each MS patient, and drawn without replacement. The reference individuals had no registered MS diagnosis in 1987-2006.

For peer review only - http://bmjopen.bmj.com/site/about/guidelines.xhtml

In total, 2650 PwMS and 7950 reference individuals were included. The year a panel was constituted with newly diagnosed PwMS and reference individuals is, hereafter, referred to as "the year of inclusion" (T_0). Observation spanned from ten years prior (T_{-10}) to the year of inclusion (T_0) until five years after inclusion (T_{+5}).

Socio-demographic and income variables were obtained from the Longitudinal Integration Database for Health Insurance and Labour Market Studies (LISA), maintained by Statistics Sweden, for each of the 16 years studied (T_{-10} until T_{+5}) given residence in Sweden at the end of the respective year.

Outcome variables

Earnings were defined as the annual sum of pre-tax earnings and student allowances. Levels of earnings before tax were measured over the 16 study years (T_{-10} to T_{+5}). Student allowances were included in earnings to limit the risk of underestimating younger individuals' ability to be in paid work in earlier study years. In the Swedish public SA system, the employer usually pays for day 2-14 of a SA spell.[41] This amount could not be disentangled, and was therefore included in our definition of earnings.[42]

All analyses of earnings were performed using 2005 monetary values in Swedish Krona (SEK) inflated by Statistics Sweden's Consumer Price Index. The annual earnings variable was constructed as an untransformed continuous variable.

An additional outcome measure of the annual proportion of individuals on part- or full-time SA and/or DP was constructed and calculated over the follow-up period. The universal Swedish SA insurance system includes two main benefits: SA and DP. From the age of 16, people with income from work or unemployment benefits can be granted SA if disease or injury leads to reduced work capacity. People aged 19-64 can be granted DP if they have long-term or permanently reduced work capacity due to disease or injury. Both benefits can

For peer review only - http://bmjopen.bmj.com/site/about/guidelines.xhtml

be granted at four levels (25, 50, 75, or 100%) of ordinary working hours, but at different replacement rates (SA: approximately 80%, DP: approximately 64% of lost income, up to a ceiling). Regarding SA, after an initial uncompensated ("waiting") day, the employer pays the next 14 days of the SA spell. A physician's certificate is required by day eight. SA spells are from day 15 covered by the Swedish Social Insurance Agency (from day 2 among unemployed).[41]

Explanatory variables

The explanatory variables were: sex (*female, male*), age (categorised in *five-year intervals*), country of birth (*Sweden, outside of Sweden*), educational level (*elementary school*, *high school, university/college*), and type of living area (based on population density: s*maller municipalities, medium-sized municipalities, larger cities*). Further, adopting a definition set by Statistics Sweden (based on annual income and verifications from the workplace) called work status, individuals were categorised as being in work during the year in question (*yes*, *no*).[42 43] Values for variables pertained to the 31 December of the year of inclusion, except for the type of living area and work status variables, which were obtained annually.

Annual information on the individuals' type of occupation was also included. Usually, measures of occupation categorise occupations hierarchically, based on the level of specialised skill.[44] Such categorisation is used by Statistics Sweden with ten mutually exclusive groups.[42] However, we also aimed to differentiate between types of occupations by how physically demanding they were, and to what extent the individuals were able to have control over their working hours (i.e., flexibility at the workplace). Therefore, we collapsed the categories into the following five groups:

For peer review only - http://bmjopen.bmj.com/site/about/guidelines.xhtml

1. **Manager**: Individuals classified as managing a group of workers. Managers performed either office work or manual labour. Therefore, this group was analysed separately.
2. **Office work**: Non-physical (“white collar”) work.
3. **Manual labour**: Physically demanding (“blue collar”) work.
4. **Workplace unknown**: Registered as in work but with no available information on the workplace.
5. **No work**: Not categorised as being in work (i.e., work status: no).

Individuals identified as employed by the armed forces were excluded from the study population (n=18; <0.1%), due to insufficient information to classify occupation and a very small number of observations.

Study population

Individuals with missing values for at least one of the socio-demographic variables in the year of inclusion were excluded using list-wise deletion, with the exception of type of occupation. Individuals who had earnings higher than the 99th percentile (in this study) in at least one year were also excluded to control for extreme outliers (around 64% of those excluded in this step had earnings above the 99th percentile for more than one year). In total, 97 PwMS and 366 references were excluded from the study, due to employment in the armed forces, missing values, or having earnings higher than the 99th percentile in a single year. Accordingly, due to these exclusions, the total number of references does not sum to three times the number of PwMS.

The maximum number of years with information for each of the individuals in the study was 16. Due to migration before/after the year of inclusion or death, not all individuals were in the study population for the entire study period. Nonetheless, 94.4% of PwMS and 93.1% of

For peer review only - http://bmjopen.bmj.com/site/about/guidelines.xhtml

227 matched references were in the study population for all 16 years. Stratified analyses by
228 country of birth showed that 97.2% of PwMS and 97.5% of the references born in Sweden
229 were included for all 16 years. Corresponding proportions for individuals not born in Sweden
230 were 70.7% and 70.5%, respectively. At the end of follow-up (T_{+5}), 2.0% of all PwMS and
231 2.1% of the references were no longer in the study population (due to migration or death).

232 **Patient and public involvement**

233 Due to the use of data from total population registers informed consent was not applicable.

234 **Ethical approval**

235 The project was approved by the Regional Ethical Review Board of Stockholm, Sweden.

236 **Statistical methods**

237 The levels and distributions of earnings were analysed for PwMS and references, stratified by
238 time before and after the year of inclusion (T_0). The four panels were pooled in all analyses.

239 Firstly, annual mean and median earnings were used to describe the central tendencies among
240 PwMS and references. The annual "interquartile range" (IQR); the distance between the 25th
241 and 75th percentile was used to describe potential converging/diverging trends within, and
242 between, the two groups.

243 Secondly, mean earnings before and after T_0, stratified by educational level and type of
244 occupation at T_0 were calculated. Two-sided t-tests tested the differences in mean earnings
245 between PwMS and the references.

246 Thirdly, the proportions of individuals on part- or full-time SA and DP during follow-up,
247 stratified by educational level and type of occupation, were examined for both the PwMS and
248 the references. For individuals with multiple spells of SA or DP in a single year, the spell
249 with the highest grade (25, 50, 75, or 100%) was selected.

For peer review only - http://bmjopen.bmj.com/site/about/guidelines.xhtml

Finally, Tobit regression models were used to control for potential socio-demographic confounding in analyses and to estimate differences in levels of earnings between PwMS and references. Earnings are a non-negative outcome and often exhibit a skewed distribution with clustering at zero (i.e., distinct differences in distributions between those in paid work compared to those not in paid work). Therefore, earnings were used as an observable proxy for individuals' ability to support themselves through their work (i.e., earnings above zero). Tobit models avoid the potential bias of analysing outcomes with "semi-continuous" distributions by ordinary least squares (OLS), which may produce negative predicted values due to assumptions of unlimited linearity in the outcome variable.[45] A Tobit model jointly estimates, using a set of explanatory variables, the probability that the individuals' outcome variable is within a pre-determined bound and the linear relationship between the explanatory variables and the outcome for individuals within the bound.[46] We used the lower bound zero (i.e., no earnings), to observe the actual outcomes, rather than unknown outcomes based on set bound(s), which also means that the observed zeroes were "genuine zeroes".[47]

Separate Tobit regressions were estimated at the time points T_0 and T_{+5} by two different models. The distributions of earnings for each of these time points were investigated and both exhibited clustering at zero earnings as shown in Figure 1. The two models used the same set of explanatory variables (MS (*yes/no*), panel membership, sex, age at inclusion, country of birth, and type of living area), with the exception of level of education and type of occupation, which due to high correlation were analysed separately. Model 1a included the level of education at year of inclusion whereas Model 2a included a time-varying variable for the type of occupation. Furthermore, to estimate the potential additional effects of different levels of education or types of occupation on the relationship with levels of earnings, two Tobit models were estimated including two-way interactions between MS and education (Model 1b, university/college as reference) and MS and occupation (Model 2b, Office work

For peer review only - http://bmjopen.bmj.com/site/about/guidelines.xhtml

as reference), respectively. Statistical tests of whether the estimates deviate enough from zero to reject the assumption of no association were performed at the 95% significance level ($p<0.05$) and presented as beta estimates with 95% confidence intervals (95% CI). Data management and statistical analyses (regression models) were conducted in SAS V.9.2, with the exception of the differences in mean earnings calculations, which were calculated in Microsoft Excel.

RESULTS

We identified 2553 PwMS (Panel: 2003: 724; 2004: 648; 2005: 636; and 2006: 545) and included 7584 matched references. The distributions of the socio-demographics for the pooled panels at the year of inclusion (T_0) are presented in Table 1. The majority of the PwMS were women (70%) and aged 30-45 when diagnosed (62%). In the year of inclusion, the majority had at least some high school education, were born in Sweden (89% among the PwMS compared to 84% in the reference group) and lived in larger cities or medium-sized municipalities. Also, in both groups, most individuals had "office work" or "manual labour" as occupations.

For peer review only

291 **Table 1**: Descriptive statistics for the people with multiple sclerosis (PwMS) group and the stratified
292 matched reference group at the year of inclusion (T_0). [a]

		PwMS N=2553[c]	%	References N=7584[c]	%
**Sex** [b]					
	Female	1796	70	5380	71
	Male	757	30	2204	29
**Age** [b]					
	30-34	494	19	1461	19
	35-39	531	21	1576	21
	40-44	574	22	1701	22
	45-49	492	19	1446	19
	50-54	462	18	1400	18
**Educational level**					
	University/college	945	37	2685	35
	High school	1345	53	3801	50
	Elementary school	263	10	1098	14
**Type of occupation**					
	Managers	86	3	259	3
	Office work	1082	42	3020	40
	Manual labour	916	36	2976	39
	Unknown	130	5	352	5
	No work	339	13	977	13
**Type of living area**					
	Larger cities	960	38	2789	37
	Medium-sized municipalities	902	35	2681	35
	Smaller municipalities	691	27	2114	28
**Country of birth**					
	Sweden	2280	89	6356	84
	Outside of Sweden	273	11	1228	16
**Year of inclusion (T_0)**					
	2003	724	28	2150	28
	2004	648	25	1927	25
	2005	636	25	1890	25
	2006	545	21	1617	21

293 [a] Year of inclusion (T_0) for PwMS: the year of first registered MS diagnosis. Year of inclusion (T_0) for references: year of inclusion to one of
294 the four panels.
295 [b] Variables used for stratified matching (1_{MS}->3_{REF}).

For peer review only

297 There were differences observed between the PwMS and the reference group in the development and intra-year distribution of earnings in the study period (Figure 2). The reference group experienced a steady increase in earnings and a predominantly even IQR across the entire period (T_{-10} to T_{+5}), whereas the PwMS' trajectory can be divided into three periods: T_{-10} to T_{-2}; T_{-1} to T_{+1}; and T_{+2} to T_{+5}. The first period (T_{-10} to T_{-2}) was characterised by increasing earnings for PwMS with an even intra-year distribution (measured by IQR). In the second period (T_{-1} to T_{+1}), PwMS' earnings were decreasing and the IQR was diverging. In the third period (T_{+2} to T_{+5}), the level of earnings was stable but IQR diverging.

When comparing differences in mean levels of earnings among PwMS and references, PwMS overall had lower earnings compared to the references for the study years T_{-1}, T_0 and T_{+5} (Table 2). These differences in mean annual earnings increased with time. In T_0, PwMS' mean earnings were 85% of the references' mean earnings, whereas the corresponding proportion in T_{+5} was 73%.

Earnings stratified by educational level and differences in earnings between the PwMS and the corresponding reference subgroup were measured for the study years T_{-5}, T_{-2}, T_{-1}, T_0, and T_{+5} (Table 2). PwMS with at most elementary or high school education, experienced a decrease in mean earnings from T_{-1} and onwards. For PwMS with university/college education, there was a decrease in mean earnings from T_{-1} to T_0, but an increase from T_0 to T_{+5}. The reference group had increasing trends in mean earnings for all levels of education; which resulted in increasing differences in mean earnings between PwMS and the references. In the last study year (T_{+5}) the largest differences in earnings were found among those with at most elementary school education; where the PwMS' mean earnings were SEK 84,000, corresponding to 53% of the mean earnings of their peers in the reference group. Furthermore, PwMS with high school and university/college education also had lower earnings than their peers in the reference group. The corresponding differences and

For peer review only - http://bmjopen.bmj.com/site/about/guidelines.xhtml

proportions for high school and university/college education were SEK 68,000 (68%) and SEK 63,000 (78%), respectively.

Likewise, differences in earnings between the PwMS and the references at T_{-5}, T_{-2}, T_{-1}, T_{0}, and T_{+5}, were stratified by type of occupation at inclusion (Table 2). In all time periods, for both PwMS and references, the mean earnings were highest among the managers (Table 2). Further, office workers had higher mean earnings compared to those with manual work. These findings reflected those which we obtained for level of education. PwMS in manual work had lower earnings compared to their reference counterparts in the final study year (T_{+5}) (67%). The corresponding figure for office workers was 78%. However, the largest difference in earnings in the final study year was observed among managers, with SEK 113,000 less among PwMS than their peers, which corresponded to 70% of the mean earnings of the reference group managers. Finally, those with no work at inclusion had much lower levels of earnings compared to those with known occupations both prior to and after this time point.

For peer review only

For peer review only - http://bmjopen.bmj.com/site/about/guidelines.xhtml

337 **Table 2**: Mean earnings[a] of people with multiple sclerosis (PwMS) and differences in mean earnings
338 between the PwMS group and matched reference group at different lengths in time from the year of
339 inclusion (T_0), stratified by educational level and type of occupation at T_0.

	T_{-5} PwMS [b] (diff)[c]	T_{-2} PwMS [b] (diff) [c]	T_{-1} PwMS [b] (diff) [c]	T_0 PwMS [b] (diff) [c]	T_{+5} PwMS [b] (diff) [c]
**All**	169 (4)	182 (1)	181 (-6)*	163 (-28)*	166 (-63)*
**Educational level**					
University/college	192 (5)	214 (1)	214 (-6)	203 (-24)*	218 (-63)*
High school	159 (0)	168 (-6)	166 (-12)*	146 (-37)*	145 (-68)*
Elementary school	135 (9)	142 (12)	135 (3)	111 (-25)*	84 (-74)*
**Type of occupation**					
Managers	251 (-12)	308 (-5)	308 (-24)	306 (-27)	258 (-113)*
Office work	207 (2)	230 (-5)	233 (-11)*	219 (-33)*	226 (-65)*
Manual labour	163 (6)	179 (4)	178 (-4)	152 (-36)*	146 (-71)*
Unknown	96 (6)	90 (-5)	99 (-7)	102 (-18)	88 (-69)*
No work	65 (13)*	39 (10)*	20 (6)*	4 (0)	25 (-27)*

340 [a] Earnings: pre-tax earnings including student allowances, presented in 2005 values as 1000 Swedish Krona (SEK).
341 [b] PwMS (n=2553). References (n=7584).
342 [c] Differences in mean earnings (SEK 1000) between PwMS and references in parentheses. Calculated with two-sided t-tests
343 with unequal variance and unequal numbers of observations.
344 * Differences significantly different from zero ($p<0.05$).
345

For peer review only

The proportions of individuals with at least one spell of part- or full-time SA and similarly DP, were larger among the PwMS than in the corresponding group among the references for both T_0 and T_{+5} (Table 3). In the diagnosis year (T_0), 46.7% of the PwMS had at least one full-time SA spell and 10.0% had part-time SA. Among PwMS, the proportion of part- and full-time SA decreased with time, whereas the proportion of DP increased substantially. In the last year of follow-up, 21.0% of the PwMS had part-time DP, and the proportion of full-time DP was 25.1%. The patterns were similar for the reference group, but at lower levels than among the PwMS.

For PwMS, the proportions of part- and full-time SA at T_{+5} were somewhat similar for all levels of education, however, the proportions varied more by type of occupation (Table 3). Among the PwMS, managers and individuals with no work had lower proportions of part- and full-time SA than PwMS in the other types of occupations. For PwMS in the last study year, there was a larger proportion of individuals with part-time DP than full-time DP among those with university/college education and with more qualified types of occupations.

For peer review only - http://bmjopen.bmj.com/site/about/guidelines.xhtml

Table 3: Percentages[a] of the people with multiple sclerosis (PwMS) and matched reference group who had sickness absence[b] and disability pension[b] in the year of inclusion (T_0) and five years later (T_{+5}), stratified by educational level and type of occupation at T_0.

Year	Factor	Category	Sickness absence				Disability pension			
			PwMS[c]		Ref[c]		PwMS[c]		Ref[c]	
			Part-time	Full-time	Part-time	Full-time	Part-time	Full-Time	Part-time	Full-time
**T_0**	**Education**	University/college	11.0	43.0	2.6	10.0	3.6	3.8	1.5	2.0
		High school	9.1	49.5	2.1	15.6	5.7	10.0	2.5	6.4
		Elementary school	11.0	45.6	1.3	16.3	8.4	22.8	3.5	16.9
**T_{+5}**		University/college	7.2	17.7	1.6	8.4	21.6	15.4	2.1	2.8
		High school	5.3	17.1	0.8	11.9	20.9	27.3	3.0	8.1
		Elementary school	6.6	13.2	0.8	10.0	19.7	48.3	4.9	19.6
**T_0**	**Occupation**	Managers	16.3	31.4	0.8	5.8	1.2	0.0	1.2	0.4
		Office work	13.3	43.6	2.9	11.0	5.4	1.9	2.0	0.7
		Manual labour	8.5	55.7	2.1	17.2	5.1	3.0	2.7	1.4
		Unknown	10.0	43.9	2.3	17.1	12.3	13.1	2.3	7.7
		No Work [d]	2.1	37.2	0.3	15.0	3.2	49.3	2.4	39.8
**T_{+5}**		Managers	4.7	8.2	0.4	4.7	24.7	15.3	1.2	0.8
		Office work	8.0	18.7	1.5	8.7	25.5	12.7	2.5	1.5
		Manual labour	5.4	19.1	0.8	13.6	20.2	23.7	3.5	2.9
		Unknown	7.9	13.4	1.7	11.3	24.2	33.6	2.9	10.4
		No Work [d]	1.9	8.5	0.8	6.8	6.3	69.7	3.2	45.5
**T_0**	**All**	-	**10.0**	**46.7**	**2.1**	**13.7**	**5.2**	**9.1**	**2.3**	**6.3**
**T_{+5}**		-	**6.2**	**16.9**	**1.1**	**10.4**	**21.0**	**25.1**	**2.9**	**7.9**

[a] Row percent of all individuals (including those without sickness absence (SA) or disability pension (DP) in each sub-group.

[b] Individuals with a spell of SA or DP with the highest grade (25, 50, 75 or 100%) at T_0 and T_{+5} (i.e., if an individual had both part- and full-time SA in a single year, the individual was categorised as full-time SA. The same procedure was used for individuals on DP).

[c] Numbers at year T_0: PwMS (n=2553) and references (n=7584).

[d] Individuals with unemployment benefits can be granted SA if the disease/injury is severe enough to prevent them from seeking employment. DP can be granted until the age of 65. In most cases, SA refers to compensation for a SA spell >14 days (the first 14 are covered by the employer).

For peer review only

The estimates from the regression Models 1a, including educational level, and 2a, including type of occupation, revealed a negative association between MS and earnings (Table 4). In T_0, the estimates for MS (yes/no) showed similar negative relationships with earnings when comparing the regression that included educational level (T_0: -36.9; 95% CI: -43.0, -30.8) and that which included type of occupation (T_0: -33.3; 95% CI: -38.0, -28.6). The corresponding estimates for five years later (T_{+5}) were also both negative; but with a larger difference between the estimates when including educational level (T_{+5}: -84.4; 95% CI: -91.5, -77.3) compared to type of occupation (T_{+5}: -49.9; 95% CI: -55.6, -44.2).

A hierarchic pattern in the estimates for educational level and type of occupation was observed. Higher levels of education (i.e., university/college) and more qualified occupations (i.e., managers and office workers) were associated with relatively higher levels of earnings compared to those with lower levels of education (i.e., high school and elementary school) or less qualified occupations.

For peer review only

Table 4: Cross-sectional Tobit regressions for earnings [a] for the year of inclusion [b] and five years later.

	Education		Occupation	
	Model 1a		Model 2a	
	Year of inclusion (**T_0**) Estimated difference [c] (95% CI)	Five years later (**T_{+5}**) Estimated difference [c] (95% CI)	Year of inclusion (**T_0**) Estimated difference [c] (95% CI)	Five years later (**T_{+5}**) Estimated difference [c] (95% CI)
Intercept	217.3 (208.3, 226.3)*	285.8 (275.2, 296.4)*	241.7 (234.6, 248.8)*	279.4 (271.2, 287.6)*
MS (ref: No MS) [d]	-36.9 (-43.0, -30.8)*	-84.4 (-91.5, -77.3)*	-33.3 (-38.0, -28.6)*	-49.9 (-55.6, -44.2)*
Male (ref: Female) [d]	68.1 (62.4, 73.8)*	67.8 (61.1, 74.5)*	63.4 (58.9, 67.9)*	64.2 (58.9, 69.5)*
Age (ref: 30-34 years) [d]				
- 35-39	17.5 (9.3, 25.7)*	20.6 (11.0, 30.2)*	9.6 (3.1, 16.1)*	18.2 (10.8, 25.6)*
- 40-44	35.5 (27.5, 43.5)*	25.1 (15.7, 34.5)*	25.7 (19.2, 32.2)*	27.4 (20.0, 34.8)*
- 45-49	33.4 (25.0, 41.8)*	6.1 (-3.9, 16.1)	24.3 (17.6, 31.0)*	16.6 (8.8, 24.4)*
- 50-54	30.0 (21.6, 38.4)*	-9.4 (-19.4, 0.6)	22.3 (15.6, 29.0)*	9.6 (1.6, 17.6)*
Country of birth (ref: Sweden) [d]	-78.4 (-86.0, -70.8)*	-71.7 (-80.7, -62.7)*	-20.3 (-26.6, -14.0)*	-19.9 (-27.3, -12.5)*
Panel (ref: 2006) [d]				
-2003	-5.1 (-12.5, 2.3)	-9.4 (-18.2, -0.6)*	-6.9 (-12.8, -1.0)*	-9.3 (-16.2, -2.4)*
-2004	-5.7 (-13.3, 1.9)	-2.1 (-11.1, 6.9)	-7.3 (-13.4, -1.2)*	-2.5 (-9.6, 4.6)
-2005	-7.8 (-15.4, -0.2)*	-6.7 (-15.7, 2.3)	-6.0 (-12.1, 0.1)	-3.1 (-10.2, 4.0)
Type of living area (ref: Larger cities) [e]				
- Medium-sized municipalities	-19.9 (-26.0, -13.8)*	-23.3 (-30.6, -16.0)*	-20.8 (-25.7, -15.9)*	-21.9 (-27.6, -16.2)*
- Smaller municipalities	-26.1 (-32.8, -19.4)*	-32.7 (-40.5, -24.9)*	-29.5 (-34.8, -24.2)*	-31.6 (-37.9, -25.3)*
Educational level (ref: University/college) [d]				
- High school	-58.1 (-63.8, -52.4)*	-79.7 (-86.4, -73.0)*	-	-
- Elementary school	-113.2 (-121.8, -104.6)*	-148.6 (-158.8, -138.4)*	-	-
Type of occupation (ref: Office work) [e]				
- Managers	-	-	63.5 (52.3, 74.7)*	87.8 (76.8, 98.8)*
- Manual labour	-	-	-65.6 (-70.1, -61.1)*	-72.5 (-77.6, -67.4)*
- Unknown	-	-	-135.9 (-145.5, -126.3)*	-171.6 (-187.3, -155.9)*
- No Work	-	-	-384.3 (-394.9, -373.7)*	-519.7 (-538.9, -500.5)*
Sigma	131.2 (129.6, 132.9)*	152.4 (150.4, 154.4)*	100.7 (99.4, 102.0)*	113.8 (112.3, 115.3)*
N	10,137	9,928	10,137	9,928
N censored at zero	1,260	1,505	1,260	1,505
Bayesian information criterion (BIC)	114,727	111,972	107,869	104,307

[a] Earnings: pre-tax earnings including student allowances. Presented in 2005 values, in SEK 1000.
[b] Year of inclusion (T_0) for people with multiple sclerosis (PwMS): year of first MS diagnosis. Year of inclusion (T_0) for references: year of inclusion to one of four panels. Estimates from Tobit models with lower bound set at zero (i.e., no earnings).
[c] Beta estimate
[d] Time-invariant variables: measured at T_0.
[e] Time-variant variables: measured at T_0 or T_{+5}.
* Differences significantly different from zero (p<0.05).

For peer review only

For peer review only - http://bmjopen.bmj.com/site/about/guidelines.xhtml

399 In the additional Tobit regressions for earnings in the year of inclusion and five years later (Models 1b and 2b), interactions between MS and education or occupation, respectively, were included (Supplementary Table 1). In Model 1b, we observed that the interaction between high school education and MS was negative in T_0 (-16.9; 95% CI: -29.6, -4.2). In T_{+5}, the interaction between elementary school education and MS was also negative (-42.7; 95% CI: -68.0, -17.4). Further, interactions between occupation and MS (Model 2b) found a negative association among managers in T_{+5} (-40.7; 95% CI: -68.5, -12.9). Whereas a non-significant negative association was found among manual labour in both T_0 (-5.0; 95% CI: -15.2, 5.2) and T_{+5} (-5.6; 95% CI: -17.8, 6.6).

For peer review only - http://bmjopen.bmj.com/site/about/guidelines.xhtml

DISCUSSION

In this cohort study we identified 2553 PwMS of working ages and compared them with 7584 references without MS across time points for a period of 16 years. The principal findings were that both groups had similar levels of earnings and annual changes in earnings up until one year before the diagnosis year, thereafter, the growth in earnings levelled off among the PwMS for the remainder of the study period, while earnings for the references continued to increase. These differences remained when we controlled for a number of socio-demographic variables. The largest differences in absolute terms between PwMS and the references were observed for those with elementary school education and for those working as managers. Those with elementary school education also had the largest relative differences. There were also large within-group differences in the development of mean earnings for the PwMS with respect to educational level, and type of occupation. Additionally, there were higher proportions among PwMS of part- and full-time SA and DP after the year of MS diagnosis compared to among the references.

This study has several strengths, including identifying the PwMS from nationwide patient registers.[48] Furthermore, the data from the nationwide registers are of high quality,[49] which made it possible to conduct our analyses with no loss to follow-up and without self-selection bias. One limitation is that the onset of MS always precedes the diagnosis, and this time lag varies considerably depending on the experienced symptoms, the individual's healthcare-seeking behaviour, healthcare practices, and timeliness to confirm diagnosis.[2 50] Therefore, the year of diagnosis used in this study can be seen as a biased proxy (although with known direction) for the year of MS onset. Without additional MS-specific data on clinical progression, we could not evaluate the degree to which this time lag affected the findings. The extent to which the time lag correlates with socio-demographic factors is, to the best of

For peer review only - http://bmjopen.bmj.com/site/about/guidelines.xhtml

our knowledge, not known. Furthermore, educational level and type of occupation were highly correlated, and therefore, analysed separately. Hence, when we analysed one of the variables, the effect of the other variable could, to some degree, be incorporated indirectly. The generalisability of the findings may be limited to countries with a similarly functioning labour market and welfare system. Finally, due to data limitations, our definition of earnings includes shorter spells of SA (days 2-14), which may conceal the initial effects of MS, and underestimate the potential effect of MS on earnings for individuals with repeated short SA spells.

Our overall results are in line with previous studies of PwMS, which have shown non-increasing earnings,[15] and high levels of unemployment or SA/DP after the MS diagnosis.[3 13 14 32 34 38 51-53] We found that differentiating the analyses by either educational level or type of occupation overall gave similar results. Nonetheless, the combination of occupation and annual earnings provided additional information, including information on those who were working compared with those not in paid work. Furthermore, by comparing the PwMS' earnings with that of their reference peers by occupation we showed that PwMS classified as managers had larger differences in earnings than those in office work. However, it is possible that the large aggregates chosen for categorisation of occupation disguised within-group variation in terms of the physical demands of work tasks. Likewise, the lack of differentiation within the university/college educational category could have concealed strong heterogeneity.

We found considerable heterogeneity within the group of PwMS; while a large proportion appeared to participate on the labour market to a high extent, many had low or no earnings at all. There are many possible explanations of this heterogeneity, with the individuals' educational level and type of occupation as two influential factors within this study. This suggests that the PwMS' work situations are important determinants of subsequent labour market outcomes. We also observed that PwMS with university/college education or with

For peer review only - http://bmjopen.bmj.com/site/about/guidelines.xhtml

more qualified types of occupations (i.e., office workers or managers) had higher proportions of part-time DP (rather than full-time DP) than those with lower education levels or less qualified types of work (i.e, manual workers or those with no work). This could potentially reflect the nature of physical work demands among manual occupations or reflect a lower degree of flexibility to adjust one's work situation than among non-manual occupations, which consequently may lead to higher rates of full-time rather than part-time DP. It was not possible to capture this distinction in the present study. However, it is also possible that our variables, educational level and type of occupation, were proxies for less tangible individual factors. One such factor could be healthcare seeking behaviour, where those with a higher educational level or more qualified occupations may receive the MS diagnosis, and thus treatment, earlier in the disease trajectory. Nevertheless, MS affected an individual's work capacity regardless of whether a diagnosis had been set or not. The new generation of disease modifying treatments to slow disease progression, and thus preserve work capacity, were first introduced in 2006, but not administered to the majority of PwMS until years later.[54] Therefore, it is possible that this could partly explain the observed differences in earnings between the groups. Similarly, the wide distribution of earnings among PwMS could reflect the known heterogeneous progression of the disease - irrespective of educational level or type of occupation.

Furthermore, the large differences in earnings among individuals with educational levels less than university/college following MS diagnosis compared to the reference group, provides support for the conclusion that the degree of physical labour and/or flexibility at the work place is of importance for remaining in paid work once diagnosed with MS. However, further research is required to explain the observed differences between PwMS and the references in managerial positions.

For peer review only - http://bmjopen.bmj.com/site/about/guidelines.xhtml

A possible explanation of the observed increasing differences in earnings between PwMS and individuals without MS, in addition to working fewer hours, and situations of pay for performance, could be due to different opportunities for mobility in the labour market. An individual's wage development is generally associated with possibilities for changing employers. This is especially so in Sweden, with largely centrally negotiated wage increases and often limited individual performance variation.[55 56] Thus, labour market mobility provides an opportunity for substantial wage improvement, and an MS diagnosis may deter changing employment.[57] More studies of PwMS' or more generally, on chronically ill individuals' wages and labour market mobility are needed to increase our knowledge about such mechanisms, e.g., by studying transitions between different occupations, types of employment, and unemployment.[58 59]

Levels and development of earnings are known to differ by both age and sex.[60] Accordingly, in the present study we used both of these variables in selecting our matched reference group. Future studies focused on how a diagnosis with MS leads to deviations from the expected earnings trajectory by age and sex are warranted. Trajectories of PwMS' mean earnings stratified by age group and sex in a previous study suggest there are differences in both patterns and levels of earnings among PwMS.[61] However more detailed analyses, including a reference group, are needed. This is especially so given that the progression of MS is to some degree age-dependent.[30]

Lastly, health outcomes research is limited by a lack of measures reflecting the multitude of MS consequences.[62] The traditional MS-specific health outcome measurement tool, Expanded Disability Systems Score (EDSS), typically overemphasises the importance of motor function and poorly reflects cognitive functions and fatigue,[62] two factors strongly influencing work capacity.[18 62-64] Our results suggest that earnings could potentially be a

For peer review only - http://bmjopen.bmj.com/site/about/guidelines.xhtml

promising outcome measure assessing a composite of functions.[18] The underlying assumption is that the reduction of earnings among PwMS to a great extent reflects MS-related disability. The observed impact of MS on earnings among PwMS across occupational types and educational levels indicates strongly that motor function, which is known to deteriorate slowly, at least in younger patients, is not the sole reason for the decrease in earnings. Thus, indicating the possible influence of fatigue and cognition.[18 63] Furthermore, our findings of earnings development reflect the same trends as the pattern of disability development, as assessed by EDSS, suggesting that earnings may serve as a surrogate outcome measure reflecting the broader consequences of MS.[18] Earnings, when available from public registers, such as from tax authorities, have the additional benefits of minimal data loss and reflecting comprehensive time periods, whereas EDSS outcomes reflect single points in time.[18] Further studies are required with data sets containing both earnings and EDSS scores to compare the two in terms of resolution and validity.

CONCLUSIONS

From one year prior to the MS diagnosis and onwards, we observed increasingly higher levels of mean earnings among the reference group compared to among the PwMS. Further, we observed considerable within-group heterogeneity among the PwMS. After five years of being diagnosed, a large percentage of PwMS still participated in the labour market, nevertheless, many had low or no earnings at all. We also found that those with at most elementary or high school levels of education or less qualified types of occupations were less alike their respective peers in the reference group than those with university/college levels of education and more qualified types of occupations. Moreover, part- and full-time SA and DP were more common among the PwMS – a result that could potentially be used to describe the observed heterogeneity of earnings among the PwMS.

For peer review only - http://bmjopen.bmj.com/site/about/guidelines.xhtml

530 ## List of abbreviations used

531 CI: Confidence intervals.

532 DP: Disability pension.

533 EDSS: The expanded disability systems score.

534 IQR: Interquartile range.

535 MS: Multiple sclerosis.

536 OLS: Ordinary least squares.

537 PwMS: People with multiple sclerosis.

538 SA: Sickness absence.

539 SEK: Swedish Krona.

For peer review only

For peer review only - http://bmjopen.bmj.com/site/about/guidelines.xhtml

Declarations

Ethics approval

The project was approved by the Regional Ethical Review Board, Stockholm, Sweden.

Acknowledgements

Not applicable

Authors’ contributions

MW, PT, KA, EP, MS, and EF participated in the design of the study. MW performed the data management and performed all analyses. All authors (MW, CM, PT, KA, EP, JH, MS, and EF) contributed to the interpretations of the results. MW and CM drafted the manuscript, PT, KA, EP, JH, MS, and EF contributed by revising for important intellectual content. All authors have approved the final version of the manuscript.

Competing interests statement

MW, CM, PT and EF were partly funded by Biogen. ED and MS have no competing interests to declare. KA has received unrestricted grants from Biogen. JH received honoraria for serving on advisory boards for Biogen and Novartis and speaker’s fees from Biogen, Merck-Serono, Bayer-Schering, Teva and Sanofi-Aventis. He has served as P.I. for projects sponsored by, or received unrestricted research support from, Biogen, Merck-Serono, TEVA, Novartis, and Bayer-Schering. His MS research is funded by the Swedish Research Council.

Funding

The project was jointly funded by an unrestricted research grant from Biogen and a research grant from the Swedish Research Council for Health, Working Life and Welfare. The design of the study, data collection, analyses, interpretations of data, and writing of manuscript was performed without involvement of the funding bodies. Biogen was given the opportunity to comment on the manuscript before submission.

Data statement

Not applicable. The data cannot be made publically available due to privacy regulations. According to the General Data Protection Regulation, the Swedish, the Swedish Data Protection Act, the Swedish Ethical Review Act, and the Public Access to Information and Secrecy Act, data can only be made available for specific purposes, including research that meets the criteria for access to this type of sensitive and confidential data as determined by a legal review. Readers may contact Professor Kristina Alexanderson (kristina.alexanderson@ki.se) regarding the data.

For peer review only

LEGENDS

Figure One

Figure 1: Distribution of earnings in the total study population, at year of inclusion (n=10,137) and five years later (n=9928), in 1000 Swedish Krona (SEK).

Earnings: Pre-tax earnings including student allowance, calculated in 2005 monetary values. The years of inclusion (T_0) vary between 2003-2006. They are for PwMS the year of the first MS diagnosis and for references the year of inclusion into one of the four panels.

Figure Two

Figure 2: Distribution (box and whisker plots) of earnings by time, for PwMS (n=2553) and matched references (n=7584), in 1000 Swedish Krona (SEK).

Earnings: Pre-tax earnings including student allowance, calculated in 2005 monetary values. The years of inclusion (T_0) vary between 2003-2006. They are for PwMS the year of the first MS diagnosis and for references the year of inclusion into one of the four panels. Observations that were larger than 1.5*IQR (Interquartile Range) are circled. Symbols in the box plot: bar = median earnings, diamond = mean earnings. Line across box-plots = median earnings for the reference group at T_0 (SEK 204,850).

For peer review only - http://bmjopen.bmj.com/site/about/guidelines.xhtml

References

1. Tullman MJ. A review of current and emerging therapeutic strategies in multiple sclerosis. *Am J Manag Care* 2013;19(2 Suppl):S21-7.
2. Brundin L, Kobelt G, Berg J, et al. New insights into the burden and costs of multiple sclerosis in Europe: Results for Sweden. *Mult Scler* 2017;23:179-91. doi: 10.1177/1352458517708682
3. Pearson J, Alla S, Clarke S, et al. Multiple Sclerosis impact on employment and income in New Zealand. *Acta Neurol Scand* 2017;136(3):223–32. doi: 10.1111/ane.12714
4. Krause I, Kern S, Horntrich A, et al. Employment status in multiple sclerosis: impact of disease-specific and non-disease-specific factors. *Mult Scler* 2013;19(13):1792–99. doi: 10.1177/1352458513485655
5. Naci H, Fleurence R, Birt J, et al. Economic burden of multiple sclerosis: a systematic review of the literature. *Pharmacoeconomics* 2010;28(5):363-79. doi: 10.2165/11532230-000000000-00000
6. Pfleger CCH, Flachs EM, Koch-Henriksen N. Social consequences of multiple sclerosis (1): early pension and temporary unemployment—a historical prospective cohort study. *Mult Scler* 2010;16(1):121-26.
7. Pugliatti M, Rosati G, Carton H, et al. The epidemiology of multiple sclerosis in Europe. *Eur J Neurol* 2006;13(7):700-22.
8. Chruzander C, Tinghög P, Ytterberg C, et al. Longitudinal changes in sickness absence and disability pension, and associations between disability pension and disease-specific and contextual factors and functioning, in people with multiple sclerosis. *J Neurol Sci* 2016
9. Ernstsson O, Gyllensten H, Alexanderson K, et al. Cost of Illness of Multiple Sclerosis-A Systematic Review. *PLoS One* 2016;11(7):e0159129.
10. Gyllensten H, Wiberg M, Alexanderson K, et al. Costs of illness of multiple sclerosis in Sweden: a population-based register study of people of working age. *Eur J Health Econ* 2018;19(3):435-46. doi: 10.1007/s10198-017-0894-6 [published Online First: 2017 May 9]
11. Gyllensten H, Wiberg M, Alexanderson K, et al. Comparing costs of illness of multiple sclerosis in three different years: A population-based study. *Mult Scler* 2018;24(4):520-28. doi: 10.1177/1352458517702549 [published Online First: 2017 Apr 3]
12. Green G, Todd J, Pevalin D. Biographical disruption associated with multiple sclerosis: using propensity scoring to assess the impact. *Soc Sci Med* 2007;65(3):524-35. doi: 10.1016/j.socscimed.2007.03.007
13. Wiberg M, Friberg E, Stenbeck M, et al. Sources and level of income among individuals with multiple sclerosis compared to the general population: A nationwide population-based study. *Multiple Sclerosis Journal* 2015;21(13):1730-41. doi: 10.1177/1352458515570767
14. Jennum P, Wanscher B, Frederiksen J, et al. The socioeconomic consequences of multiple sclerosis: a controlled national study. *Eur Neuropsychopharmacol* 2012;22(1):36-43. doi: 10.1016/j.euroneuro.2011.05.001
15. Landfeldt E, Castelo-Branco A, Svedbom A, et al. Personal Income Before and After Diagnosis of Multiple Sclerosis. *Value Health* 2018;21(5):590-95. doi: https://doi.org/10.1016/j.jval.2017.09.021 [published Online First: 2017 Nov 15]
16. Murley C, Mogard O, Wiberg M, et al. Trajectories of disposable income among people of working ages diagnosed with multiple sclerosis: a nationwide register-based cohort study in Sweden 7 years before to 4 years after diagnosis with a population-based reference group. *BMJ Open* 2018;8(5):e020392. doi: 10.1136/bmjopen-2017-020392 [published Online First: 2018/05/11]
17. Thormann A, Sørensen P, Koch-Henriksen N, et al. Chronic comorbidity in multiple sclerosis is associated with lower incomes and dissolved intimate relationships. *Eur J Neurol* 2017;24(6):825-34. doi: 10.1111/ene.13297

For peer review only - http://bmjopen.bmj.com/site/about/guidelines.xhtml

18. Kavaliunas A, Wiberg M, Tinghog P, et al. Earnings and Financial Compensation from Social Security Systems Correlate Strongly with Disability for Multiple Sclerosis Patients. *PLoS One* 2015;10(12):e0145435. doi: ARTN e014543510.1371/journal.pone.0145435
19. Abegunde DO, Stanciole AE. The economic impact of chronic diseases: how do households respond to shocks? Evidence from Russia. *Soc Sci Med* 2008;66(11):2296-307.
20. Carlsson KS, Landin-Olsson M, Nyström L, et al. Long-term detrimental consequences of the onset of type 1 diabetes on annual earnings—evidence from annual registry data in 1990–2005. *Diabetologia* 2010;53(6):1084-92.
21. Løkke A, Hilberg O, Kjellberg J, et al. Economic and Health Consequences of COPD Patients and Their Spouses in Denmark-1998-2010. *COPD* 2014;11(3):237-46.
22. Mitchell JM, Burkhauser RV, Pincus T. The importance of age, education, and comorbidity in the substantial earnings losses of individuals with symmetric polyarthritis. *Arthritis Rheum* 1988;31(3):348-57.
23. Shanahan EM, Smith MD, Roberts-Thomson L, et al. The effect of rheumatoid arthritis on personal income in Australia. *Intern Med J* 2008;38(7):575-79.
24. Ward MM, Javitz HS, Smith WM, et al. Lost income and work limitations in persons with chronic respiratory disorders. *J Clin Epidemiol* 2002;55(3):260-68.
25. Wolfe F, Michaud K, Choi HK, et al. Household income and earnings losses among 6,396 persons with rheumatoid arthritis. *J Rheumatol* 2005;32(10):1875-83.
26. Confavreux C, Vukusic S, Adeleine P. Early clinical predictors and progression of irreversible disability in multiple sclerosis: an amnesic process. *Brain* 2003;126(Pt 4):770-82.
27. Ruggieri R, Palermo R, Vitello G, et al. Cognitive impairment in patients suffering from relapsing-remitting multiple sclerosis with EDSS≤ 3.5. *Acta Neurol Scand* 2003;108(5):323-26.
28. Degenhardt A, Ramagopalan SV, Scalfari A, et al. Clinical prognostic factors in multiple sclerosis: a natural history review. *Nat Rev Neurol* 2009;5(12):672-82.
29. Langer-Gould A, Popat RA, Huang SM, et al. Clinical and demographic predictors of long-term disability in patients with relapsing-remitting multiple sclerosis: a systematic review. *Arch Neurol* 2006;63(12):1686-91.
30. Confavreux C, Vukusic S. Age at disability milestones in multiple sclerosis. *Brain* 2006;129(Pt 3):595-605. doi: 10.1093/brain/awh714
31. Beatty WW, Blanco CR, Wilbanks SL, et al. Demographic, clinical, and cognitive characteristics of multiple sclerosis patients who continue to work. *Neurorehabil Neural Repair* 1995;9(3):167-73.
32. Busche K, Fisk J, Murray T, et al. Short Term Predictors of Unemployment in Multiple Sclerosis Patients. *Can J Neurol Sci* 2003;30(2):137-42. doi: doi.org/10.1017/S0317167100053403
33. Grønning M, Hannisdal E, Mellgren SI. Multivariate analyses of factors associated with unemployment in people with multiple sclerosis. *J Neurol Neurosurg Psychiatry* 1990;53(5):388-90.
34. Lunde HMB, Telstad W, Grytten N, et al. Employment among Patients with Multiple Sclerosis-A Population Study. *PLoS One* 2014;9(7):e103317.
35. Patti F, Pozzilli C, Montanari E, et al. Effects of education level and employment status on HRQoL in early relapsing-remitting multiple sclerosis. *Mult Scler* 2007;13(6):783-91.
36. Roessler RT, Rumrill PD, Fitzgerald SM. Predictors of employment status for people with multiple sclerosis. *Rehabil Couns Bull* 2004;47(2):96-103.
37. Smith MM, Arnett PA. Factors related to employment status changes in individuals with multiple sclerosis. *Mult Scler* 2005;11(5):602-09.
38. Glad SB, Nyland H, Aarseth JH, et al. How long can you keep working with benign multiple sclerosis? *J Neurol Neurosurg Psychiatry* 2011;82(1):78-82. doi: 10.1136/jnnp.2010.210732 [published Online First: 2010/08/31]
39. Moore P, Harding KE, Clarkson H, et al. Demographic and clinical factors associated with changes in employment in multiple sclerosis. *Mult Scler* 2013:1352458513481396.

For peer review only - http://bmjopen.bmj.com/site/about/guidelines.xhtml

40. Findling O, Baltisberger M, Jung S, et al. Variables Related to Working Capability among Swiss Patients with Multiple Sclerosis—A Cohort Study. *PLoS One* 2015
41. Social Insurance in Figures 2014. Stockholm, Sweden, 2014.
42. Background Facts, Labour and Education Statistics 2011:4, Integrated database for labour market research. Stockholm: Statistics Sweden, 2011.
43. Rikets indelningar: årsbok över regionala indelningar med koder, postadresser, telefonnummer [Country classifications: yearbook of regional classifications with codes, postal addresses, phone numbers 2003]. Stockholm: Statistics Sweden, 2003.
44. SSYK 96: Standard för svensk yrkesklassificering 1996 [SSYK 96: Standard for Swedish occupation classification 1996]. Swedish Standard Classification of Occupations. Örebro: Statistics Sweden, 1998.
45. Wooldridge JM. Corner solution outocmes and censored regression models. Econometric analysis of cross section and panel data. Cambridge, Massachusetts: MIT Press 2002.
46. Tobin J. Estimation of relationships for limited dependent variables. *Econometrica* 1958;26(1):24-36.
47. Humphreys BR. Dealing with zeros in economic data. University of Alberta, Alberta: Department of Economics, 2013.
48. Ludvigsson JF, Andersson E, Ekbom A, et al. External review and validation of the Swedish national inpatient register. *BMC Public Health* 2011;11(1):450.
49. Ludvigsson JF, Almqvist C, Bonamy A-KE, et al. Registers of the Swedish total population and their use in medical research. *Eur J Epidemiol* 2016;31(2):125-36.
50. Thompson AJ, Banwell BL, Barkhof F, et al. Diagnosis of multiple sclerosis: 2017 revisions of the McDonald criteria. *Lancet Neurol* 2018;17(2):162-73. doi: 10.1016/S1474-4422(17)30470-2
51. Glad S, Aarseth J, Nyland H, et al. Benign multiple sclerosis: a need for a consensus. *Acta Neurol Scand* 2010;122(s190):44-50.
52. Julian LJ, Vella L, Vollmer T, et al. Employment in multiple sclerosis. *J Neurol* 2008;255(9):1354-60.
53. Simmons RD, Tribe KL, McDonald EA. Living with multiple sclerosis: longitudinal changes in employment and the importance of symptom management. *J Neurol* 2010;257(6):926-36.
54. Eriksson I, Komen J, Piehl F, et al. The changing multiple sclerosis treatment landscape: impact of new drugs and treatment recommendations. *Eur J Clin Pharmacol* 2018;74(5):663-70. doi: 10.1007/s00228-018-2429-1 [published Online First: 2018/02/13]
55. Nyberg A, Magnusson Hanson LL, Leineweber C, et al. Do Predictors of Career Success Differ between Swedish Women and Men? Data from the Swedish Longitudinal Occupational Survey of Health (SLOSH). *PLoS One* 2015;10(10):e0140516. doi: 10.1371/journal.pone.0140516 [published Online First: 2015/10/27]
56. Muffels R, Luijkx R. Labour market mobility and employment security of male employees in Europe:trade-off'orflexicurity'? *Work, employment and Society* 2008;22(2):221-42.
57. McAllister A, Nylen L, Backhans M, et al. Do 'flexicurity' Policies Work for People With Low Education and Health Problems? A Comparison of Labour Market Policies and Employment Rates in Denmark, The Netherlands, Sweden, and the United Kingdom 1990-2010. *Int J Health Serv* 2015;45(4):679-705. doi: 10.1177/0020731415600408 [published Online First: 2015/08/15]
58. Ekberg K, Wahlin C, Persson J, et al. Is mobility in the labor market a solution to sustainable return to work for some sick listed persons? *Journal of occupational rehabilitation* 2011;21(3):355-65. doi: 10.1007/s10926-011-9322-4 [published Online First: 2011/07/26]
59. Nordstrom K, Ekberg K, Hemmingsson T, et al. Sick leave and the impact of job-to-job mobility on the likelihood of remaining on the labour market--a longitudinal Swedish register study. *BMC Public Health* 2014;14:305. doi: 10.1186/1471-2458-14-305 [published Online First: 2014/04/04]
60. Women and men in Sweden 2018. Örebro, Sweden: Statistics Sweden, 2018.

For peer review only

For peer review only - http://bmjopen.bmj.com/site/about/guidelines.xhtml

61. Murley C, Yang F, Gyllensten H, et al. Disposable income trajectories of working-aged individuals with diagnosed multiple sclerosis. *Acta Neurol Scand* 2018;138(6):490-99. doi: 10.1111/ane.13001 [published Online First: 2018/07/26]

62. van Munster CEP, Uitdehaag BMJ. Outcome Measures in Clinical Trials for Multiple Sclerosis. *CNS drugs* 2017;31(3):217-36. doi: 10.1007/s40263-017-0412-5

63. Kavaliunas A, Karrenbauer VD, Tinghog P, et al. Cognitive function predicts work disability among multiple sclerosis patients. *Mult Scler* 2017;23:479-80.

64. Kavaliunas A, Karrenbauer VD, Gyllensten H, et al. Cognitive function is a major determinant of income among multiple sclerosis patients in Sweden acting independently from physical disability. *Mult Scler* 2017:1352458517740212. doi: 10.1177/1352458517740212

For peer review only

For peer review only - http://bmjopen.bmj.com/site/about/guidelines.xhtml

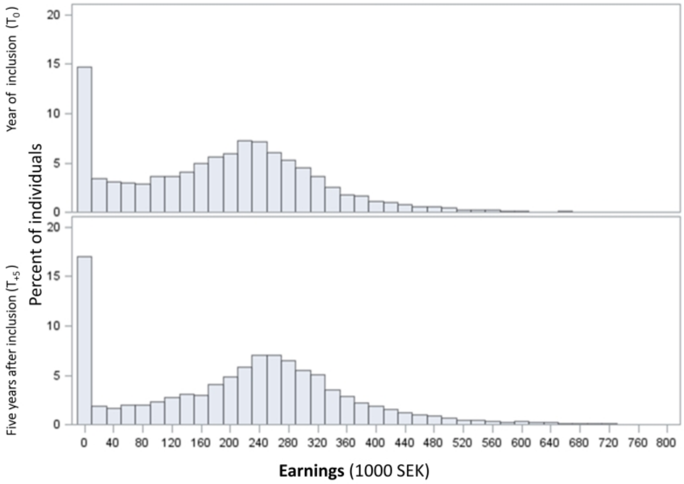

Figure 1: Distribution of earnings in the total study population, at year of inclusion (n=10,137) and five years later (n=9928), in 1000 Swedish Krona (SEK). Earnings: Pre-tax earnings including student allowance, calculated in 2005 monetary values. The years of inclusion (T_0) vary between 2003-2006. They are for PwMS the year of the first MS diagnosis and for references the year of inclusion into one of the four panels.

177x99mm (600 x 600 DPI)

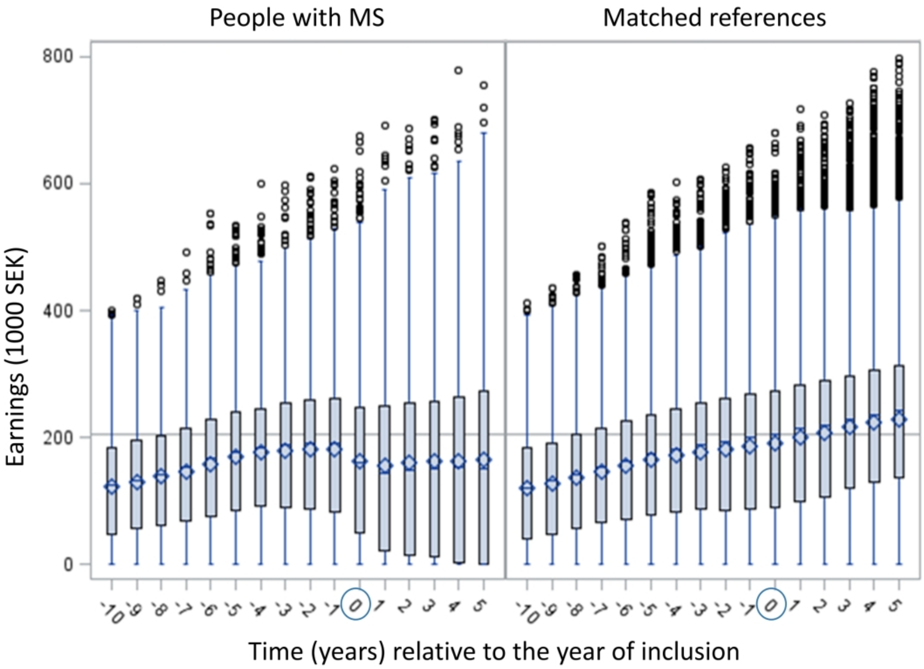

Figure 2: Distribution (box and whisker plots) of earnings by time, for PwMS (n=2553) and matched references (n=7584), in 1000 Swedish Krona (SEK). Earnings: Pre-tax earnings including student allowance, calculated in 2005 monetary values. The years of inclusion (T_0) vary between 2003-2006. They are for PwMS the year of the first MS diagnosis and for references the year of inclusion into one of the four panels. Observations that were larger than 1.5*IQR (Interquartile Range) are circled. Symbols in the box plot: bar = median earnings, diamond = mean earnings. Line across box-plots = median earnings for the reference group at T_0 (SEK 204,850).

127x92mm (600 x 600 DPI)

Supplementary Table 1: Cross-sectional Tobit regressions for earnings [a] for the year of inclusion [b] and five years later including interactions between MS and education or occupation, respectively.

	**Education** **Model 1b**		**Occupation** **Model 2b**	
	Year of inclusion (**T_0**) Estimated difference [c] (CI 95%)	Five years later (**T_{+5}**) Estimated difference [c] (CI 95%)	Year of inclusion (**T_0**) Estimated difference [c] (CI 95%)	Five years later (**T_{+5}**) Estimated difference [c] (CI 95%)
Intercept	214.7 (205.5, 223.9)*	282.8 (271.8, 293.8)*	241.8 (234.5, 249.1)*	278.2 (270.0, 286.4)*
MS (ref: No MS)[d]	-26.9 (-36.7, -17.1)*	-72.3 (-83.9, -60.7)*	-33.3 (-40.4, -26.2)*	-45.5 (-53.5, -37.5)*
Male (ref: Female)[d]	68.2 (62.5, 73.9)*	67.9 (61.2, 74.6)*	63.4 (58.9, 67.9)*	64.4 (59.1, 69.7)*
Age (ref: 30-34)[d]				
- 35-39	17.5 (9.3, 25.7)*	20.6 (11.0, 30.2)*	9.4 (2.9, 15.9)*	18.4 (11.0, 25.8)*
- 40-44	35.5 (27.5, 43.5)*	25.1 (15.7, 34.5)*	25.6 (19.3, 31.9)*	27.4 (20.0, 34.8)*
- 45-49	33.5 (25.1, 41.9)*	6.2 (-3.8, 16.2)	24.2 (17.5, 30.9)*	16.6 (8.8, 24.4)*
- 50-54	30.0 (21.6, 38.4)*	-9.5 (-19.5, 0.5)	22.2 (15.5, 28.9)*	9.7 (1.7, 17.7)*
Country of birth (ref: Sweden)[d]	-78.5 (-86.1, -70.9)*	-72.4 (-81.4, -63.4)*	-20.1 (-26.4, -13.8)*	-19.9 (-27.3, -12.5)*
Panel (ref: 2006)[d]				
-2003	-5.3 (-12.7, 2.1)	-9.5 (-18.3, -0.7)*	-6.9 (-12.8, -1.0)*	-9.4 (-16.3, -2.5)*
-2004	-5.9 (-13.5, 1.7)	-2.2 (-11.2, 6.8)	-7.3 (-13.4, -1.2)*	-2.5 (-9.6, 4.6)
-2005	-7.9 (-15.5, -0.3)*	-6.8 (-15.8, 2.2)	-6.0 (-12.1, 0.1)	-3.3 (-10.4, 3.8)
Size of living region (ref: Larger cities)[e]				
- Medium-sized municipalities	-19.7 (-25.8, -13.6)*	-23.1 (-30.4, -15.8)*	-20.8 (-25.7, -15.9)*	-21.7 (-27.4, -16.0)*
- Smaller municipalities	-26.0 (-32.7, -19.3)*	-32.7 (-40.5, -24.9)*	-29.5 (-34.8, -24.2)*	-31.5 (-37.8, -25.2)*
Education (ref: University/college)[d]				
- High school	-53.7 (-60.4, -47.0)*	-75.9 (-83.5, -68.3)*	-	-
- Elementary school	-110.3 (-119.9, -100.7)*	-139.9 (-151.3, -128.5)*	-	-
- MS* High school	**-16.9 (-29.6, -4.2)***	**-14.9 (-30.0, 0.2)**	-	-
- MS* Elementary school	**-11.6 (-32.4, 9.2)**	**-42.7 (-68.0, -17.4)***	-	-
Type of Occupation (ref: Office work)[e]				
- Managers	-	-	61.2 (48.3, 74.1)*	95.7 (83.5, 107.9)*
- Manual labour	-	-	-64.5 (-69.6, -59.4)*	-71.2 (-77.1, -65.3)*
- Unknown	-	-	-138.4 (-149.6, -127.2)*	-165.2 (-183.4, -147.0)*
- No Work	-	-	-390.5 (-402.5, -378.5)*	-523.7 (-546.4, -501.0)*
- MS*Managers	-	-	**8.9 (-16.6, 34.4)**	**-40.7 (-68.5, -12.9)***
- MS* Manual labour	-	-	**-5.0 (-15.2, 5.2)**	**-5.6 (-17.8, 6.6)**
- MS* Unknown	-	-	**8.9 (-12.7, 30.5)**	**-24.7 (-60.2, 10.8)**
- MS* No Work	-	-	**27.0 (3.7, 50.3)***	**14.8 (-26.4, 56.0)**
Sigma	131.1 (129.1, 133.1)*	152.3 (149.9, 154.7)*	100.6 (99.0, 102.2)*	113.7 (111.9, 115.5)*
N	10,137	9,928	10,138	9,928
N censored at zero	1,260	1,505	1,260	1,505
Bayesian information criterion (BIC)	114,739	111,979	107,898	104,333

[a] Earnings: pre-tax earnings including student allowances. Presented in 2005 values, in SEK 1000.
[b] Year of inclusion (T_0) for people with multiple sclerosis (PwMS): year of first MS diagnosis. Year of inclusion (T_0) for references: year of inclusion to one of four panels. Estimates from Tobit models with lower bound set at zero (i.e., no earnings).
[c] Beta estimate
[d] Time-invariant variables: measured at T_0.
[e] Time-variant variables: measured at T_0 or T_{+5}.
* Differences significantly different from zero ($p<0.05$).

For peer review only

STROBE 2007 (v4) checklist of items to be included in reports of observational studies in epidemiology*

Checklist for cohort, case-control, and cross-sectional studies (combined)

Section/Topic	Item #	Recommendation	Reported on page #
**Title and abstract**	1	(*a*) Indicate the study's design with a commonly used term in the title or the abstract	1 (title)<br>2 (abstract)
		(*b*) Provide in the abstract an informative and balanced summary of what was done and what was found	2-3
**Introduction**			
Background/rationale	2	Explain the scientific background and rationale for the investigation being reported	5-6
Objectives	3	State specific objectives, including any pre-specified hypotheses	6-7 (in the form of study aims)
**Methods**			
Study design	4	Present key elements of study design early in the paper	7 first paragraph of Methods
Setting	5	Describe the setting, locations, and relevant dates, including periods of recruitment, exposure, follow-up, and data collection	7-8
Participants	6	(*a*) *Cohort study*—Give the eligibility criteria, and the sources and methods of selection of participants. Describe methods of follow-up<br>*Case-control study*—Give the eligibility criteria, and the sources and methods of case ascertainment and control selection. Give the rationale for the choice of cases and controls<br>*Cross-sectional study*—Give the eligibility criteria, and the sources and methods of selection of participants	Cohort study<br>7-10.
		(*b*) *Cohort study*—For matched studies, give matching criteria and number of exposed and unexposed<br>*Case-control study*—For matched studies, give matching criteria and the number of controls per case	
Variables	7	Clearly define all outcomes, exposures, predictors, potential confounders, and effect modifiers. Give diagnostic criteria, if applicable	8- outcome variable<br>8-9 explanatory variables
Data sources/ measurement	8*	For each variable of interest, give sources of data and details of methods of assessment (measurement). Describe comparability of assessment methods if there is more than one group	8- source<br>8-9 measurement and classification of variables
Bias	9	Describe any efforts to address potential sources of bias	12
Study size	10	Explain how the study size was arrived at	7-8

For peer review only

Quantitative variables	11	Explain how quantitative variables were handled in the analyses. If applicable, describe which groupings were chosen and why	11-12
Statistical methods	12	(*a*) Describe all statistical methods, including those used to control for confounding	11-12
		(*b*) Describe any methods used to examine subgroups and interactions	12 – by education and occupation
		(*c*) Explain how missing data were addressed	10
		(*d*) *Cohort study*—If applicable, explain how loss to follow-up was addressed<br>*Case-control study*—If applicable, explain how matching of cases and controls was addressed<br>*Cross-sectional study*—If applicable, describe analytical methods taking account of sampling strategy	10
		(*e*) Describe any sensitivity analyses	NA
**Results**			
Participants	13*	(a) Report numbers of individuals at each stage of study—eg numbers potentially eligible, examined for eligibility, confirmed eligible, included in the study, completing follow-up, and analysed	10
		(b) Give reasons for non-participation at each stage	10
		(c) Consider use of a flow diagram	NA
Descriptive data	14*	(a) Give characteristics of study participants (eg demographic, clinical, social) and information on exposures and potential confounders	13 and Table 1
		(b) Indicate number of participants with missing data for each variable of interest	10/ NA
		(c) *Cohort study*—Summarise follow-up time (eg, average and total amount)	-
Outcome data	15*	*Cohort study*—Report numbers of outcome events or summary measures over time	15-19
		Case-control study—Report numbers in each exposure category, or summary measures of exposure	
		Cross-sectional study—Report numbers of outcome events or summary measures	
Main results	16	(*a*) Give unadjusted estimates and, if applicable, confounder-adjusted estimates and their precision (eg, 95% confidence interval). Make clear which confounders were adjusted for and why they were included	20-21
		(*b*) Report category boundaries when continuous variables were categorized	NA
		(*c*) If relevant, consider translating estimates of relative risk into absolute risk for a meaningful time period	
Other analyses	17	Report other analyses done—eg analyses of subgroups and interactions, and sensitivity analyses	
**Discussion**			
Key results	18	Summarise key results with reference to study objectives	22
Limitations	19	Discuss limitations of the study, taking into account sources of potential bias or imprecision. Discuss both direction and magnitude of any potential bias	22-23
Interpretation	20	Give a cautious overall interpretation of results considering objectives, limitations, multiplicity of analyses, results from similar studies, and other relevant evidence	23-25

For peer review only - http://bmjopen.bmj.com/site/about/guidelines.xhtml

Generalisability	21	Discuss the generalisability (external validity) of the study results	23
**Other information**			
Funding	22	Give the source of funding and the role of the funders for the present study and, if applicable, for the original study on which the present article is based	28

*Give information separately for cases and controls in case-control studies and, if applicable, for exposed and unexposed groups in cohort and cross-sectional studies.
Note: An Explanation and Elaboration article discusses each checklist item and gives methodological background and published examples of transparent reporting. The STROBE checklist is best used in conjunction with this article (freely available on the Web sites of PLoS Medicine at http://www.plosmedicine.org/, Annals of Internal Medicine at http://www.annals.org/, and Epidemiology at http://www.epidem.com/). Information on the STROBE Initiative is available at www.strobe-statement.org.

For peer review only
